# Effect of a Clinical Decision Support System on Team‐Based Clinical Quiz Performance Among Japanese Medical Students: A Pre–Post Pilot Study

**DOI:** 10.1002/jgf2.70142

**Published:** 2026-06-09

**Authors:** Sho Isoda, Tadayuki Hashimoto, Toshinori Nishizawa, Satoru Sekiya, Shinya Yamanaka, Miwa Misawa, Takashi Watari, Eiichiro Ueda, Tomio Suzuki, Yasuharu Tokuda

**Affiliations:** ^1^ Department of General Medicine Osaka Medical and Pharmaceutical University Hospital Osaka Japan; ^2^ Department of Emergency Medicine Brigham and Women's Hospital Boston Massachusetts USA; ^3^ Medical Quality Management Office St. Luke's International Hospital Chūō Japan; ^4^ Department of Anesthesiology St. Luke's International Hospital Chūō Japan; ^5^ Department of Hematology St. Luke's International Hospital Chūō Japan; ^6^ Integrated Clinical Education Center Kyoto University Hospital Kyoto Japan; ^7^ Department of Hospital Quality and Safety Management Osaka Medical and Pharmaceutical University Hospital Osaka Japan; ^8^ Muribushi Okinawa Center for Teaching Hospitals Urasoe Japan

**Keywords:** clinical decision support, evidence‐based digital tool, evidence‐based medicine, medical students, quasi‐experimental study

## Abstract

**Background:**

Educational impact of point‐of‐care clinical decision support system (CDSS) on medical students remains insufficiently studied. Our aim was to evaluate the effect of a CDSS on team‐based clinical quiz performance and confidence in using medical information resources.

**Methods:**

We conducted a nationwide, online, pre–post quasi‐experimental pilot study on November 24, 2024. Eighteen teams of Japanese medical students (team size ranging from one to three members) completed ten multiple‐choice questions based on two clinical cases. Teams first answered questions using any resources except UpToDate (Pre phase), then re‐answered the same questions using UpToDate only (Post phase) under stricter time constraints. The primary outcome was team‐based quiz score. Secondary outcomes were team‐based confidence in using UpToDate and other resources. Paired *t*‐tests and Wilcoxon signed‐rank tests were performed. Sensitivity analyses addressed missing post‐intervention confidence data.

**Results:**

Seventeen teams (43 individuals) were included in the analysis. Mean quiz scores increased from 4.00 (SD 1.70) to 4.76 (SD 2.17) (paired *t*‐test *p* = 0.01; Wilcoxon *p* = 0.026). Confidence in using UpToDate improved from 1.38 (SD 0.77) to 2.54 (SD 0.97) (paired t‐test *p* = 0.0021), while confidence in other resources showed no significant change. Sensitivity analyses supported the confidence improvement under neutral and best‐case assumptions but not under the extreme worst‐case scenario.

**Conclusions:**

CDSS use may be associated with modest improvements in team‐based quiz performance and confidence among medical students in this volunteer‐based sample; generalizability to broader populations requires further investigation. Future studies should examine learning outcomes, clinical applicability, and curricular integration.

## 1 Introduction

Early exposure to evidence‐based medicine (EBM) is essential for fostering clinical practice and lifelong learning skills among future physicians. The earlier students develop the ability to search for, appraise, and apply evidence, the more likely they are to integrate EBM into their future clinical practice [1]. In the United States, numerous initiatives have been implemented to promote evidence‐based practice (EBP) among physicians, and their effectiveness has been systematically examined within a growing body of educational research [2, 3, 4]. Globally, the promotion and implementation of EBP have accelerated across healthcare systems, with evidence suggesting improvements in patient outcomes across a wide range of clinical settings [5]. A critical component of EBM competency is the ability to efficiently access and apply high‐quality clinical information at the point of care [6]—a skill that can be supported through the use of clinical decision support systems (CDSSs) [7].

The Model Core Curriculum for Medical Education in Japan, originally formulated in 2016 and revised in 2022, incorporates EBM education as an essential component of clinical training [8]. CDSSs such as UpToDate provide synthesized, expert‐reviewed clinical guidance at the point of care, potentially supporting the information‐access dimension of EBM practice. A retrospective study at a Japanese community hospital demonstrated that access to UpToDate was associated with a significant reduction in diagnostic errors in outpatient care among physicians, with error rates of 2% versus 24% (odds ratio 15.21; 95% CI: 1.86–124.36, *p* = 0.01) [9]. Whether similar benefits extend to medical students in an educational setting—particularly in terms of clinical quiz performance and confidence in using medical information resources—remains unknown.

To address this knowledge gap, we aimed to examine the effect of access to a CDSS on team‐based clinical quiz performance and confidence in using medical information resources among Japanese medical students. Through a quiz‐based intervention simulating clinical scenarios, we evaluated performance and confidence before and after the introduction of a CDSS.

## 2 Methods

This pre–post quasi‐experimental study included Japanese medical students and non‐licensed medical graduates (i.e., individuals who had completed medical school but had not yet passed the national medical licensure examination) from institutions nationwide. The online quiz‐based intervention was conducted on November 24, 2024, in collaboration with Osaka Medical and Pharmaceutical University and St. Luke's International Hospital.

**FIGURE 1:**
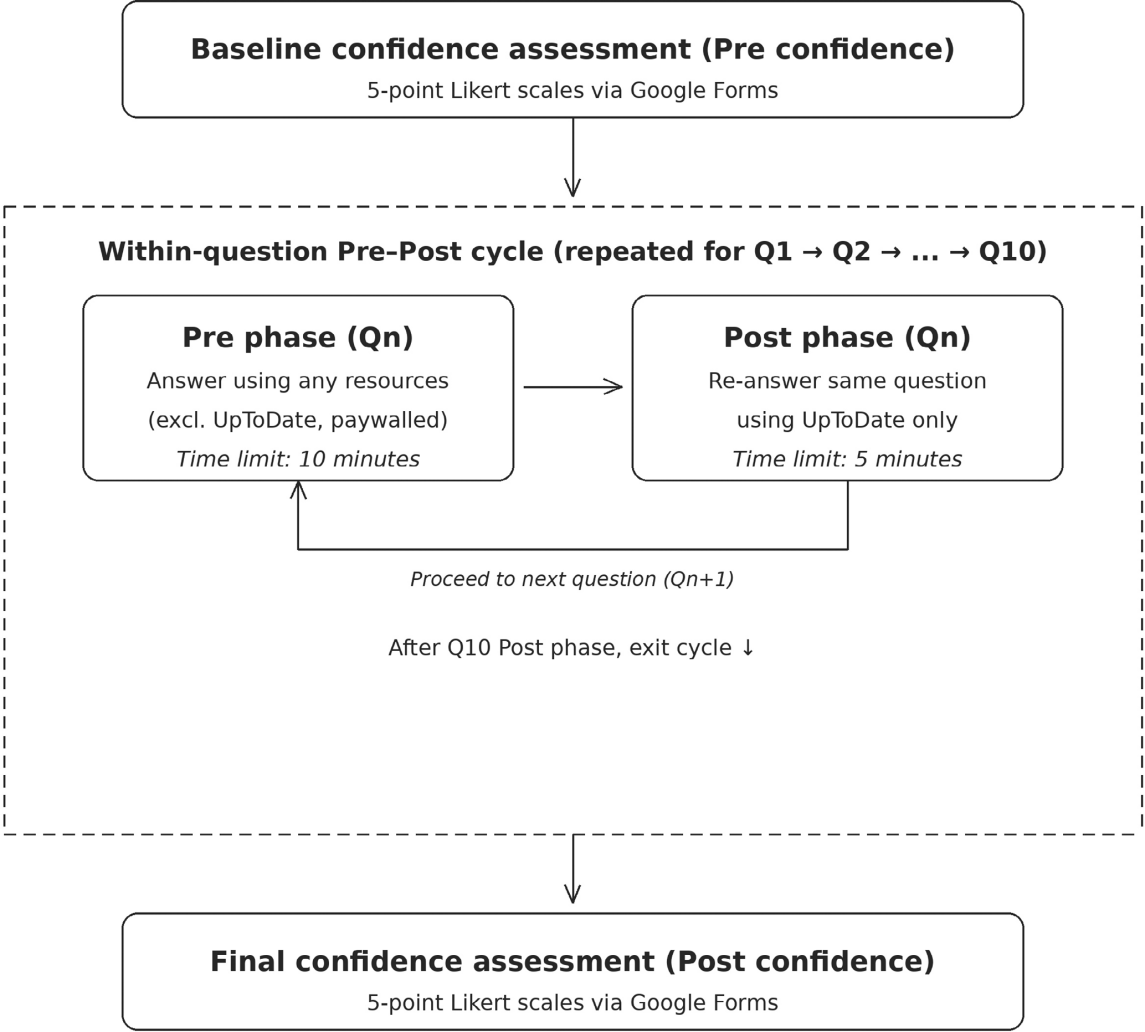
Procedural outline of the pre‐post quiz event. Each team answered Q1 through Q10 in immediate Pre‐Post sequence, confidence was assessed only at baseline and after Q10.

**FIGURE 2:**
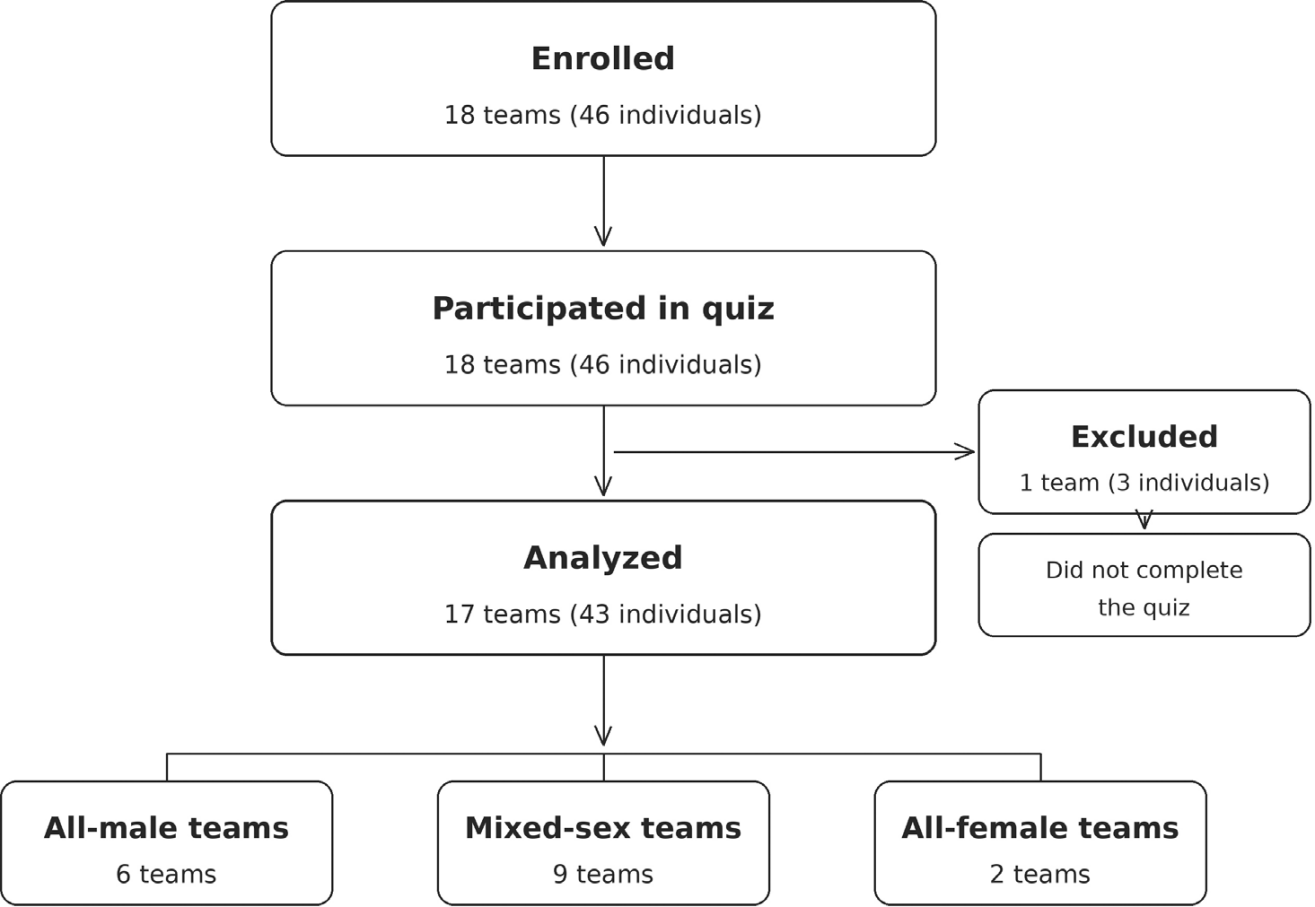
Participant flow diagram.

Participants were recruited nationwide; all Japanese medical students and non‐licensed medical graduates expressing interest were eligible. Individuals lacking access to both a personal computer and a smartphone were excluded. Potential participants were recruited via public social media announcements (Facebook) inviting Japanese medical students and non‐licensed medical graduates nationwide to participate in a voluntary educational event (“EBM Grand Prix”). No institutional mailing lists or closed channels were used; the announcement was openly accessible to any user of the relevant social media platforms. Interested individuals registered voluntarily through an online application form, with full awareness that the activity was a team‐based educational quiz event. As recruitment relied on self‐selection from social‐media‐based outreach, the resulting sample likely over‐represents individuals with pre‐existing interest in EBM and online learning. Participants formed self‐selected teams of three and answered quiz questions collaboratively (Figure 1). A total of 46 individuals (18 teams) participated. While the target team size was three members, team sizes varied. Of the 18 teams that participated, 11 comprised three members, 6 comprised two members, and 1 comprised one member, yielding a total of 46 individuals. One three‐member team did not complete the quiz and was excluded, leaving 17 teams (43 individuals) for analysis (Figure 2).

Prior to the event, a brief instructional guide for navigating UpToDate was distributed to all participants to ensure familiarity with the tool before the Post phase. Each team answered 10 multiple‐choice questions across two clinical cases—one on pancreatitis and one on heart failure—each comprising five questions in a select‐all‐that‐apply format. The clinical questions and case vignettes are provided in Appendix S1. Each question was scored using an all‐or‐nothing criterion: a team received one point only if they selected all correct options and no incorrect options. For each question, teams first answered using any freely available resources, including generative AI tools and personal textbooks, but excluding UpToDate and paywalled literature (“Pre” phase), then immediately re‐answered using UpToDate only (“Post” phase). This within‐question Pre–Post cycle was repeated sequentially from Q1 to Q10 (i.e., Q1 Pre → Q1 Post → Q2 Pre → Q2 Post → … → Q10 Pre → Q10 Post). Pre responses were limited to 10 min each, and Post responses to 5 min. This design was not adopted on the basis of established evidence that shorter Post durations reduce learning effects. As the event was conducted entirely online, compliance with resource restrictions relied on participants' self‐reported adherence and good‐faith compliance. Participants were instructed not to use UpToDate during the Pre phase and to use only UpToDate during the Post phase; however, no technical enforcement mechanisms were in place.

The primary outcome was team‐based quiz performance, defined as the number of correct answers per team (maximum score: 10 points). Secondary outcomes were team‐based confidence in using medical resources other than UpToDate and confidence in using UpToDate. Confidence was assessed using five‐point Likert scales (1 = “not confident at all”; 5 = “very confident”) administered via Google Forms at two time points: once at baseline prior to the Pre phase of Q1 (“Pre confidence”), and once after completion of the entire quiz, following the Post phase of Q10 (“Post confidence”). Accordingly, each team contributed a single Pre and a single Post confidence score. The use of immediate within‐question Pre–Post sequencing and shortened Post response times was a pragmatic design choice for this pilot study; we acknowledge that it may not have effectively minimized learning effects or recall bias (see Limitations).

### 2.1 Statistical Analyses

Quiz scores and confidence ratings were analyzed level. As a pilot study, no formal sample size calculation was performed. For the primary outcome (quiz score; range 0–10), Pre and Post performance were compared using two‐sided Wilcoxon signed‐rank tests as the primary analysis (reporting exact *p*‐values), given the bounded, discrete nature of scores and small sample size. Paired *t*‐tests were performed as secondary analyses to provide point estimates and 95% confidence intervals. Ties and zero differences used standard corrections. Questionnaire data used complete‐case analysis (missing data excluded). The primary analysis for confidence outcomes used complete‐case analysis, excluding teams with missing post‐intervention confidence data. As pre‐specified sensitivity analyses, we imputed missing post‐intervention values under three scenarios: worst case (Post = 1), neutral case (Post = Pre‐intervention value), and best case (Post = 5). These are scenario‐based bounds intended to characterize the range of plausible effects under different assumptions about missing data; they do not represent a principled imputation method for potentially informative missingness. All analyses were performed using Stata (version 18; StataCorp, College Station, TX, USA).

The “EBM Grand Prix” was originally organized and conducted as a voluntary educational event, not as a research study. Following completion of the event, the organizers developed a research protocol for retrospective analysis of the resulting team‐level data. The Ethics Review Board of Osaka Medical and Pharmaceutical University determined that formal ethical review was not required for this protocol, as it involved retrospective analysis of fully anonymized, team‐level data from a previously concluded educational activity, with no individually identifiable information used in the analysis. As the data were generated during an educational event rather than within a research framework, prospective written informed consent specifically for research participation was not obtained, and an opt‐out procedure for research use was not implemented.

## 3 Results

A total of 18 teams (46 individuals) participated. One team (3 individuals) did not complete the quiz and was excluded, leaving data from 17 teams (43 individuals) for analysis. Team sex composition included 6 all‐male teams, 2 all‐female teams, and 9 mixed‐sex teams. Of the 43 individuals included in the analysis, all were currently enrolled medical students (academic year not recorded); no non‐licensed medical graduates were included. Among the 43 participants, 27 were male and 16 were female.

The primary outcome was the number of correct answers per team. In the Pre phase (using resources other than UpToDate), the mean score was 4.00 (SD 1.70; 95% CI: 3.13–4.87). In the Post phase (using UpToDate only), the mean score increased to 4.76 (SD 2.17; 95% CI: 3.65–5.88), with a medium standardized effect size (Cohen's d_z = 0.67; 95% CI: 0.15–1.18). This improvement was statistically significant (paired *t*‐test, *p* = 0.01; Wilcoxon signed‐rank test, *z* = −2.299, exact *p* = 0.026) (Table 1). Most teams improved, while a few had decreased or unchanged scores.

**TABLE 1:** Comparison of Team‐Based Quiz Scores and Confidence Levels Before and After Using UpToDate.

Outcome	Mean (SD)	95% CI	*p*
Quiz Score (Pre)	4.00 (1.70)	3.13–4.87	
Quiz Score (Post)	4.76 (2.17)	3.65–5.88	0.01
Confidence in UpToDate (Pre)	1.38 (0.77)	0.92–1.85	
Confidence in UpToDate (Post)	2.54 (0.97)	1.95–3.12	< 0.01
Confidence in Other Resources (Pre)	2.08 (1.12)	1.40–2.75	
Confidence in Other Resources (Post)	2.46 (1.05)	1.83–3.10	0.30

*Note:* Primary analyses were performed with paired *t*‐tests.

For secondary outcomes, team‐based confidence in using UpToDate increased from 1.38 (SD 0.77) to 2.54 (SD 0.97) (paired *t*‐test, *p* = 0.0021; Wilcoxon signed‐rank test, exact *p* = 0.0049). Confidence in other resources showed no significant change (paired *t*‐test, *p* = 0.293; Wilcoxon test, *p* = 0.406).

### 3.1 Sensitivity Analyses

Four teams had missing Post confidence data. These teams had higher baseline UpToDate confidence (Pre mean 3.00 vs. 1.38; *t*‐test *p* = 0.013; Wilcoxon test *p* = 0.038), suggesting missingness was not completely at random. We imputed missing Post values under 3 scenarios (*N* = 17 teams):

Worst case (Post = 1): UpToDate confidence change (Post–Pre) mean = 0.41 (paired *t*‐test *p* = 0.361; Wilcoxon *p* = 0.325); non‐significant. Neutral case (Post = Pre): mean = 0.88 (paired *t*‐test *p* = 0.0033; Wilcoxon *p* = 0.0049); significant. Best case (Post = 5): mean = 1.35 (paired *t*‐test *p* = 0.0003; Wilcoxon *p* = 0.0006); significant.

Across all scenarios, confidence in non‐CDSS resources remained non‐significant (paired *t*‐test *p* values: 0.448, 0.289, 0.086; Wilcoxon *p* values: 0.495, 0.406, 0.125). Wilcoxon tests confirmed primary findings: quiz score improvement (exact *p* = 0.026), UpToDate confidence increase (exact *p* = 0.0049), and no change in other resources (*p* = 0.406).

## 4 Discussion

This pilot study found that access to a structured CDSS was associated with improved quiz performance and increased confidence among teams of Japanese medical students in a simulated clinical setting. Specifically, use of UpToDate was linked to better performance and greater confidence in the tool itself, while confidence in other resources remained largely unchanged. These findings suggest that access to a CDSS may be associated with improvements in team‐based quiz performance and perceived competence in using medical information resources among students with limited clinical experience. However, several alternative explanations cannot be excluded. First, the Post phase afforded additional deliberation time regardless of whether UpToDate was used effectively. Second, team discussion during the Post phase may have contributed independently to performance improvements. Third, as the unit of analysis was the team rather than the individual, these results cannot be extrapolated to individual‐level performance.

Our results aligned with previous studies showing the utility of computer‐based decision support tools in reducing diagnostic errors and improving clinical decision‐making. For example, research involving Japanese physicians found that access to a CDSS like UpToDate was associated with fewer diagnostic errors in outpatient care settings [9]. A computerized tool improved referral appropriateness and increased clinician confidence, indicating that evidence‐based practices improve patient outcomes [5]. To our knowledge, this is the first study to demonstrate these effects specifically among Japanese medical students, suggesting that early integration of CDSS may foster EBM competencies before graduation. Prior qualitative research identified gaps in EBM training and a lack of structured educational frameworks for Japanese medical students [10], underscoring the importance of targeted interventions such as ours. The observed improvement in quiz performance may be attributed to the intuitive interface, concise content summaries, and high perceived credibility of CDSS like UpToDate, which collectively enable rapid access to clinically relevant evidence [11].

In recent years, artificial intelligence (AI)‐based clinical decision support tools have rapidly advanced, attracting attention for their ability to automate the search for and summary of medical information [12]. The role of AI‐based clinical decision support tools in medical education is evolving rapidly, and their comparative utility relative to CDSSs such as UpToDate remains an important area for future empirical investigation. As our study did not include a direct comparison between these modalities, we refrain from making comparative recommendations at this time. It is also worth noting that CDSSs such as UpToDate, like AI‐based tools, require users to exercise independent clinical judgment; clinicians remain accountable for patient outcomes regardless of which reference tool they consult.

These findings have several implications for future studies. These preliminary findings suggest that access to CDSS may be worth exploring as a component of medical education for Japanese medical students. Given the volunteer‐based sampling and limited sample size, however, these results should not be generalized beyond this specific cohort without further investigation. This study offers a practical framework for evaluating EBM interventions through team‐based, quiz‐driven experimental designs [13].

This study had several limitations. First, the small sample size limits both precision and statistical power, and a planned subgroup analysis by team sex composition was therefore not performed. Second, as nationwide volunteers, participants likely had higher baseline interest in EBM, introducing selection bias and limiting generalizability. To address selection bias and missing data, we examined baseline comparability and performed sensitivity analyses for the four teams with missing post‐intervention confidence. Teams that failed to submit the post‐questionnaire had higher baseline confidence in CDSS, suggesting missingness was unlikely to be completely at random. We bounded the plausible effect by imputing absent post‐scores under three scenarios. The observed increase in CDSS confidence remained significant under neutral (Post = Pre) and best‐case (Post = 5) scenarios but lost significance under the extreme worst‐case scenario (Post = 1). Confidence in non‐CDSS resources remained non‐significant across all scenarios. These results indicate that the confidence effect is directionally consistent and statistically sensitive only to an implausibly pessimistic bound. Nevertheless, we interpret the effect size cautiously given volunteer sampling and limited sample size.

Third, the quasi‐experimental design and lack of a control group prevent conclusively isolating the CDSS's effect from alternative explanations. Importantly, the within‐question pre–post sequencing introduced a carry‐over effect: Post‐phase responses reflected cumulative knowledge acquired during both the Pre phase and the Post phase, rather than representing the isolated efficacy of the CDSS alone. Consequently, the observed score improvement is best interpreted as the “added value” or “corrective benefit” of using the CDSS following an initial information search, rather than as evidence of its standalone efficacy. Additionally, while immediate within‐question Pre–Post sequencing was originally intended to minimize recall bias, we acknowledge that this design may have paradoxically amplified the influence of recall, as participants answered the same questions twice in immediate succession. The shortened Post‐phase response time may also have introduced time–pressure effects that could have confounded the confidence measure. These testing and recall effects represent potential sources of bias that cannot be excluded in the present design. Additionally, as the study was conducted entirely online, compliance with resource restrictions could not be technically enforced and relied solely on participants' self‐reported adherence. It is possible that some participants did not fully adhere to the prescribed resource restrictions.

Fourth, quiz performance does not necessarily translate to real‐world clinical behavior. While the mean improvement of 0.76 points (out of 10) was statistically significant, its practical educational value warrants critical consideration. An absolute gain of 0.76 points on a 10‐point scale represents a modest effect, and educators should carefully weigh this benefit against the feasibility, time, and institutional costs of integrating CDSS into undergraduate curricula. In this pilot setting, the intervention was conducted online within a single session, suggesting relatively low implementation burden; however, whether such modest performance gains justify broader curricular adoption remains uncertain and requires evaluation in larger, more rigorous studies. Nevertheless, even modest gains in early‐stage learners may serve as a scaffolding function, building familiarity with evidence‐based resources that could compound over time with repeated use. Our findings alone are insufficient to justify causal claims that use of a CDSS enhances actual clinical outcomes.

The data analyzed in this study were generated during a voluntary educational event rather than within a prospective research framework. Although participants registered for the event with full awareness of its educational and quiz‐based nature, prospective written informed consent specifically for research use of the data was not obtained, nor was a formal opt‐out procedure implemented. While the institutional ethics board determined that formal review and consent procedures were not required for this retrospective analysis of fully anonymized, team‐level data, we acknowledge that this approach may not fully align with international norms for prospective research consent. Future studies of this kind should ideally be planned as research from the outset, with appropriate prospective consent procedures in place.

In conclusion, this pilot study suggests that access to a CDSS such as UpToDate may be associated with modest improvements in team‐based clinical quiz performance and confidence in using medical information resources among Japanese medical students. Incorporating such tools into undergraduate medical education is a promising area warranting further investigation. Further research should explore larger and more diverse cohorts, assess long‐term educational and clinical outcomes, evaluate applicability in real‐world settings, and determine the most effective approaches for integrating a CDSS within clinical education curricula.

## Author Contributions

**Tadayuki Hashimoto:** writing – review and editing. **Eiichiro Ueda:** supervision, conceptualization, writing – review and editing. **Tomio Suzuki:** supervision, resources, writing – review and editing. **Satoru Sekiya:** investigation, data curation, writing – review and editing. **Sho Isoda:** writing – original draft. **Yasuharu Tokuda:** project administration, writing – review and editing, conceptualization, supervision, methodology. **Takashi Watari:** supervision, writing – review and editing. **Miwa Misawa:** supervision, writing – review and editing. **Shinya Yamanaka:** methodology, writing – review and editing. **Toshinori Nishizawa:** formal analysis, writing – review and editing.

## Ethics Statement

The “EBM Grand Prix” was originally conducted as a voluntary educational event, not as a prospective research study. A research protocol for retrospective analysis of the resulting anonymized, team‐level data was subsequently submitted to the Ethics Review Board of Osaka Medical and Pharmaceutical University, which determined that formal ethical review was not required. As the data were generated during an educational event rather than within a research framework, prospective written informed consent for research participation was not obtained. All data analyzed were fully anonymized at the team level, with no individually identifiable information used.

## Conflicts of Interest

Torao Yoshida (UpToDate, Wolters Kluwer) provided administrative and technical support related to access arrangements for this study. Although the sponsor played no role in the study design, data collection, analysis, interpretation, or manuscript preparation, this relationship represents a potential conflict of interest and is hereby disclosed. All other authors declare no competing interests.

## Supporting information


**Appendix S1:** jgf270142‐sup‐0001‐AppendixS1.pdf.

## Data Availability

The data that support the findings of this study are available from the corresponding author upon reasonable request.
