# Supplementary material for: Effect of a Clinical Decision Support System on Team‐Based Clinical Quiz Performance Among Japanese Medical Students: A Pre–Post Pilot Study
Source: J Gen Fam Med. 2026 Jun 9;27(4):e70142. doi: 10.1002/jgf2.70142 (PMC13250386; doi:10.1002/jgf2.70142)
Supplement: Supplementary file 1 — Appendix S1: jgf270142‐sup‐0001‐AppendixS1.pdf. [file JGF2-27-e70142-s001.pdf]

全国医学生EBMグランプリ
オンライン大会

2024年11月24日 13時開幕

ルール説明

- 1症例につき5つの質問で構成されます。
- 1つの質問ごとに，
  前半10分(UpToDate使用なし)，後半5分(UpToDate使用あり)，
  解説の順番で進んでいきます。(口頭で回答締め切りの1分前，30秒前，
  終了のアナウンスを行います)
- 回答は1チーム代表者1人のみで，前半・後半それぞれで回答してください
  ※二重の回答は無効になります
  ※質問ごとに1つのgoogle formで回答してください
  ※Google formより登録するので，可能ならGoogleアカウントで
  回答をしてください
- 今回はブレイクアウトルームを使用しないので，チームごとに別のデバイスで議論をしてください

スケジュール

13:00 – 15:00	Case 1 提示→5問<br>(それぞれ前半10分、後半5分、解説)
15:00 – 15:10	休憩
15:10 – 17:10	Case 2 提示→5問<br>(それぞれ前半10分、後半5分、解説)
17:30	優勝チーム発表, 終了

Case 1

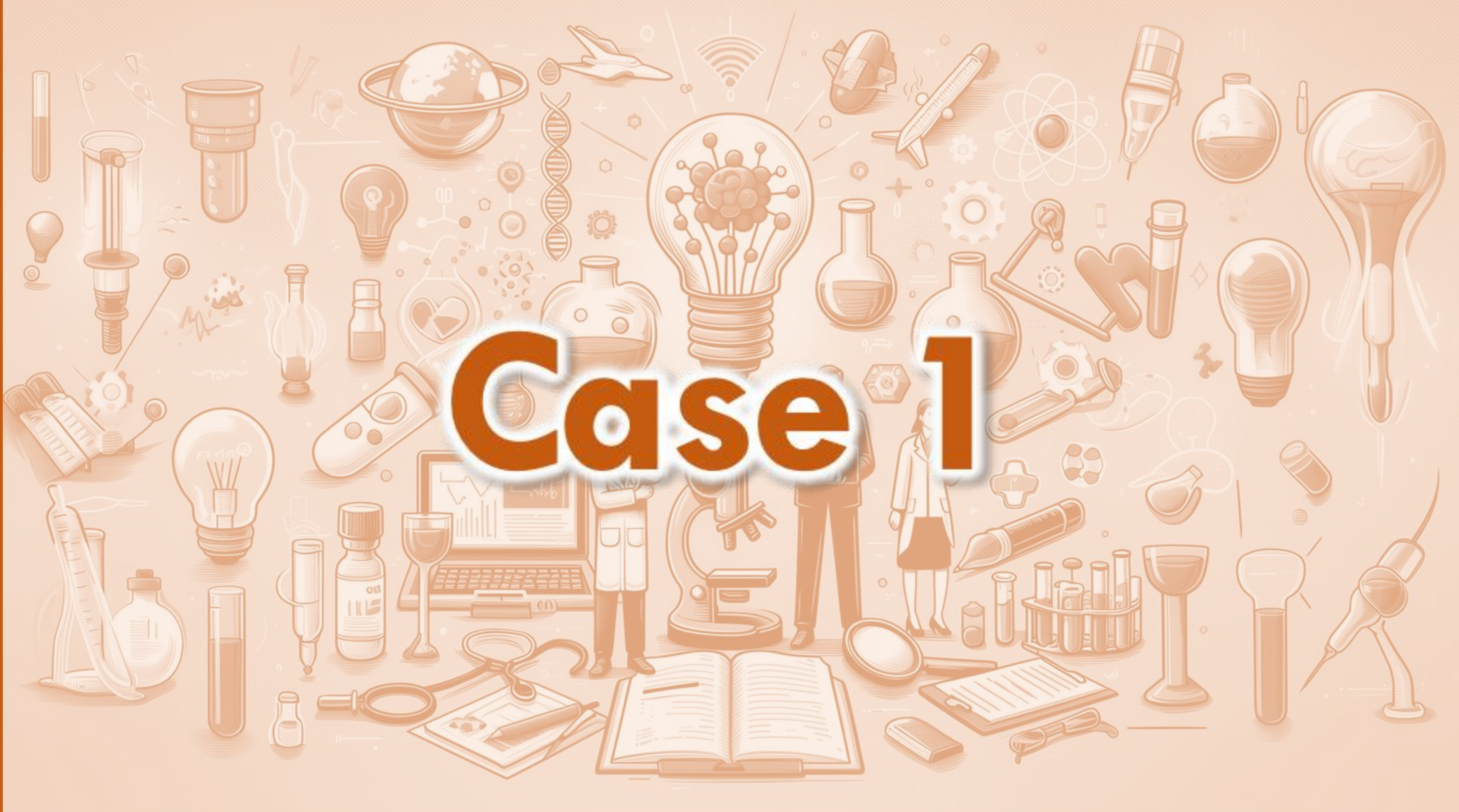

Case 1

【症例】42歳女性
【主訴】腹痛

【現病歴】2023年6月X日, 18時頃までは症状がなかった。
18時半に食事を摂取，19時頃より心窩部痛を自覚し，
食物残渣様の嘔吐が2度あった。症状の改善に乏しく，
友人に連れられて，救急外来をwalk-inで受診した。

【既往歴】特記事項なし
【生活歴】喫煙:1日5本 10年,飲酒:ビール350mL 2本 焼酎1合/日
【常用薬】低用量ピル

Case 1

【月経周期】 28日周期 整, 妊娠の可能性はなし
【摂食歴】 生もの, 刺し身, 生焼け肉などなし
【Sick Contact】 なし

【Vital Signs】 E4V5M6, 体温37.3度、血圧102/65mmHg
心拍数110bpm 整, 呼吸数 28回/分,
SpO_2 95%(RA)

【身体診察】 皮膚に明らかな色調変化を認めない
腸蠕動音低下, 腹部正中から右上腹部に圧痛あり
反跳痛なし, Murphy徴候陰性,
McBurney点圧痛なし，CVA叩打痛陰性

Case 1

血算			γ-GTP	23	IU/L	Cr	1.49	mg/dL		
WBC	12,000	/μL	T-Bil	0.4		CK	890	IU/L		
Hb	14.4	g/dL	D-Bil	0.2		CK-MB	6	IU/L		
Plt	5.3	10^4/μL	Na	133	mEq/L	AMY	1200			
凝固			K	4.2	mEq/L	P-AMY	680		血液ガス	
INR	1.03		Cl	98	mEq/L	T-Chol	128		FiO2	0.24
APTT	34	sec	IP	1.9		LDL-Chol	117		pH	7.31
生化学			Mg	1.4		HDL-Chol	45		PaO_2	84
AST	73	IU/L	Ca	8.8		CRP	14.54	mg/dL	PCO_2	39
ALT	23	IU/L	TP	6.5	g/dL	Glucose	120	mg/dL	HCO_3	19.4
LDH	650	IU/L	Alb	3.6	g/dL	HbA1c	6.3	mg/dL	BE	-6.6
ALP	684	IU/L	BUN	27.3	mg/dL	Troponin T	<0.02		Lac	3.3

Case 1

血算			γ-GTP	23	IU/L	Cr	1.49	mg/dL		
WBC	12,000	/μL	T-Bil	0.4		CK	890	IU/L		
Hb	14.4	g/dL	D-Bil	0.2		CK-MB	6	IU/L		
Plt	5.3	10^4/μL	Na	133	mEq/L	AMY	1200			
凝固			K	4.2	mEq/L	P-AMY	680		血液ガス	
INR	1.03		Cl	98	mEq/L	T-Chol	128		FiO2	0.24
APTT	34	sec	IP	1.9		LDL-Chol	117		pH	7.31
生化学			Mg	1.4		HDL-Chol	45		PaO_2	84
AST	73	IU/L	Ca	8.8		CRP	14.54	mg/dL	PCO_2	39
ALT	23	IU/L	TP	6.5	g/dL	Glucose	120	mg/dL	HCO_3	19.4
LDH	650	IU/L	Alb	3.6	g/dL	HbA1c	6.3	mg/dL	BE	-6.6
ALP	684	IU/L	BUN	27.3	mg/dL	Troponin T	<0.02		Lac	3.3

炎症反応上昇, 膵酵素上昇, 腎機能低下

心電図

HR:120 10.00mm/mV 25.0mm/s H50 d 35Hz 波形連続型:6chx2 10.00mm/mV 25.0mm/s H50 d 35Hz

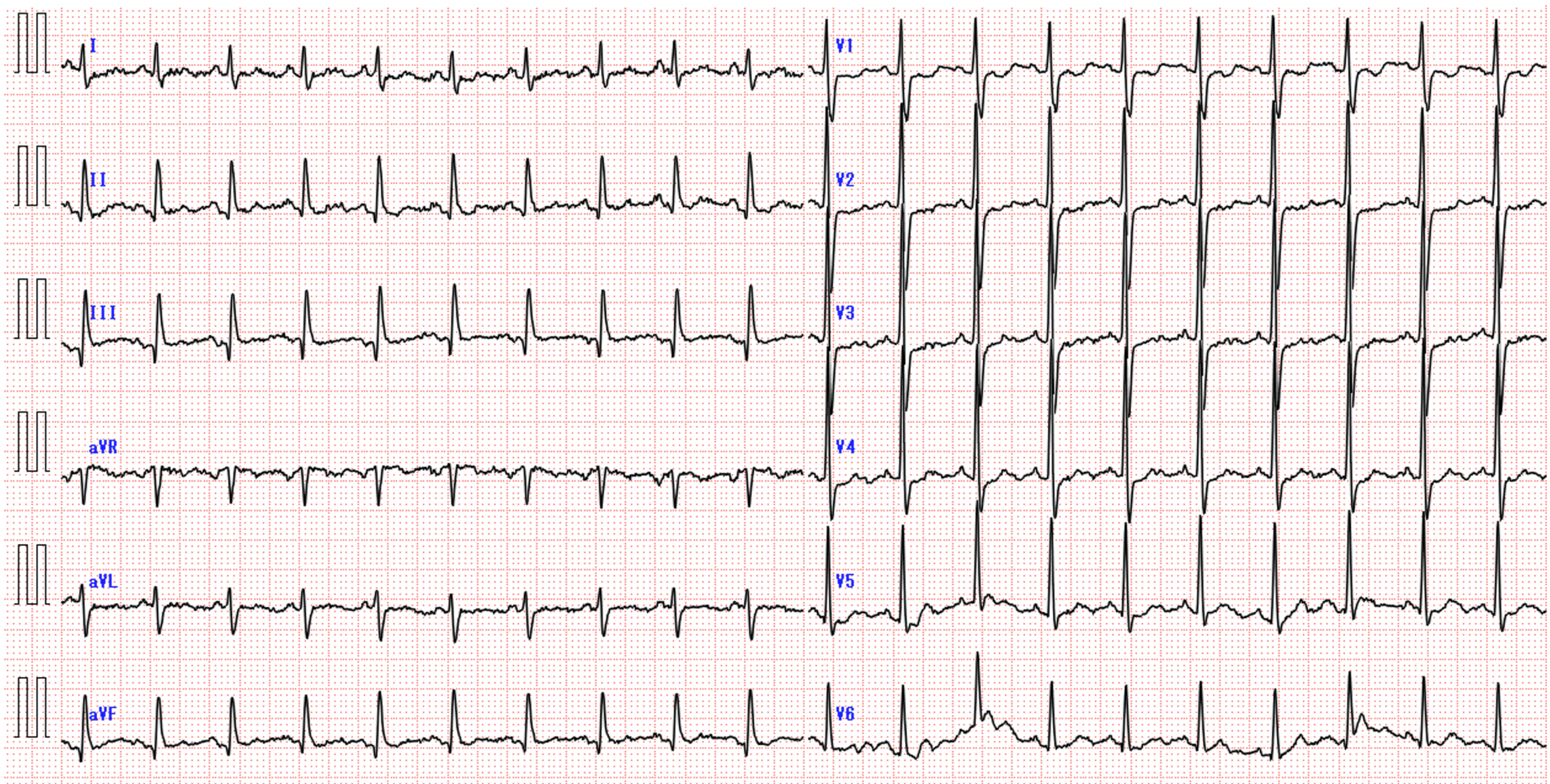

腹部造影CT検査

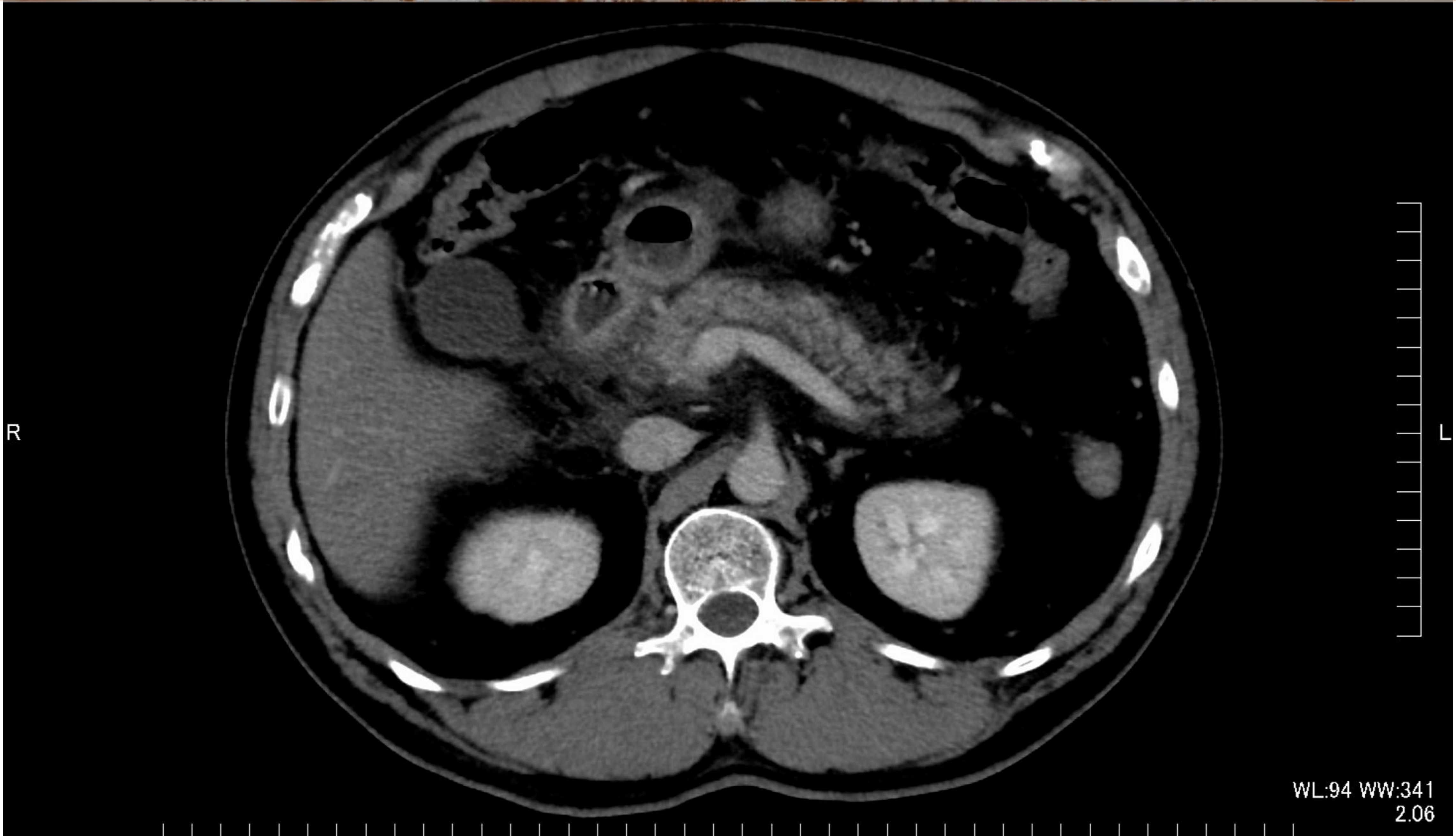

腹部造影CT検査

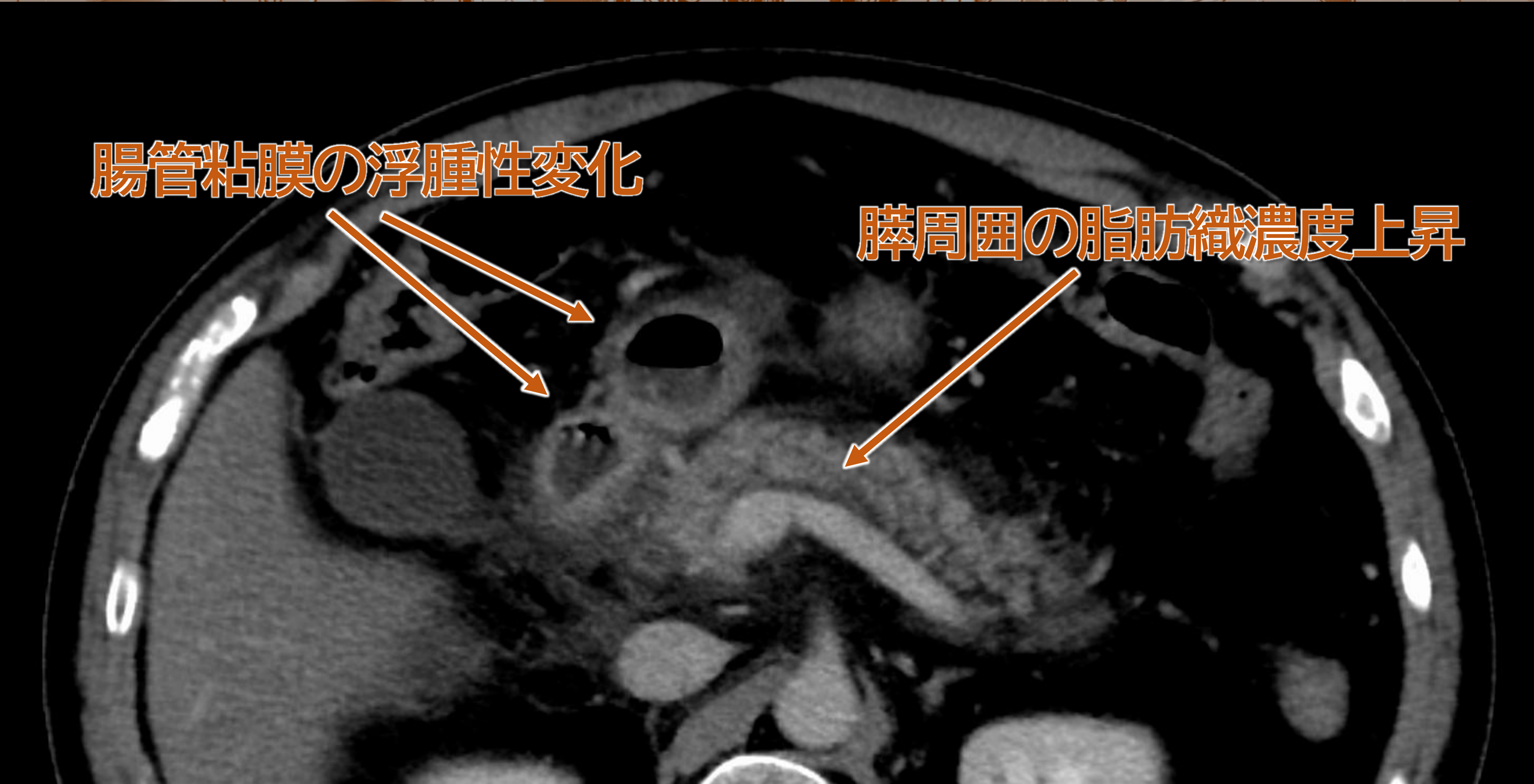

その他 各種検査

【心臓超音波検査】EF 70%, 明らかな弁膜症を認めない
壁運動低下なし, 心嚢液貯留なし

【腹部超音波検査】胆嚢軽度腫大あり、胆石や胆管内結石なし
腹水貯留なし, 両側腎盂拡張なし
膵周囲は描出不良で判然としない

【妊娠判定検査】陰性

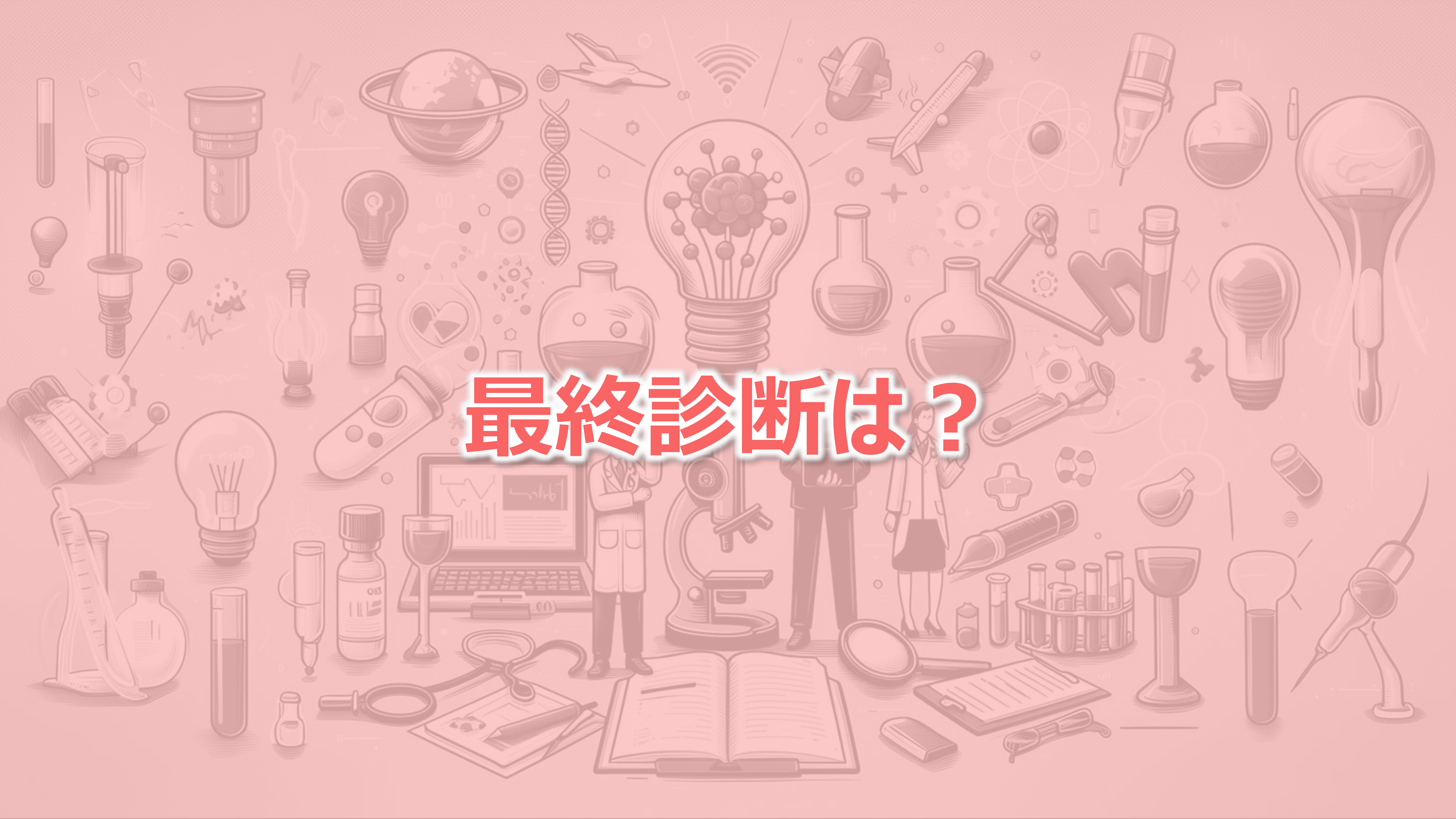

最終診断は？

【診断】
急性膵炎

膵臓の生理学

- **外分泌機能(消化酵素の分泌)：**

  膵臓は消化酵素を分泌し，膵液として十二指腸に分泌する．
  膵液には、以下の酵素が含まれています：
  - **アミラーゼ**：炭水化物を分解します。
  - **リパーゼ**：脂肪を分解します。
  - **トリプシン, キモトリプシン**：タンパク質を分解します。

- **内分泌機能（ホルモンの分泌）：**

  この機能は、膵臓の中にある「ランゲルハンス島」で行われます．
  主に分泌されるホルモンは次の2つです：
  - **インスリン**：血糖値を下げる働きがあり、体内の細胞が血液中の糖を取り込むのを助けます。
  - **グルカゴン**：インスリンとは逆に、血糖値を上げる働きがあり、肝臓からグルコースを放出させます。

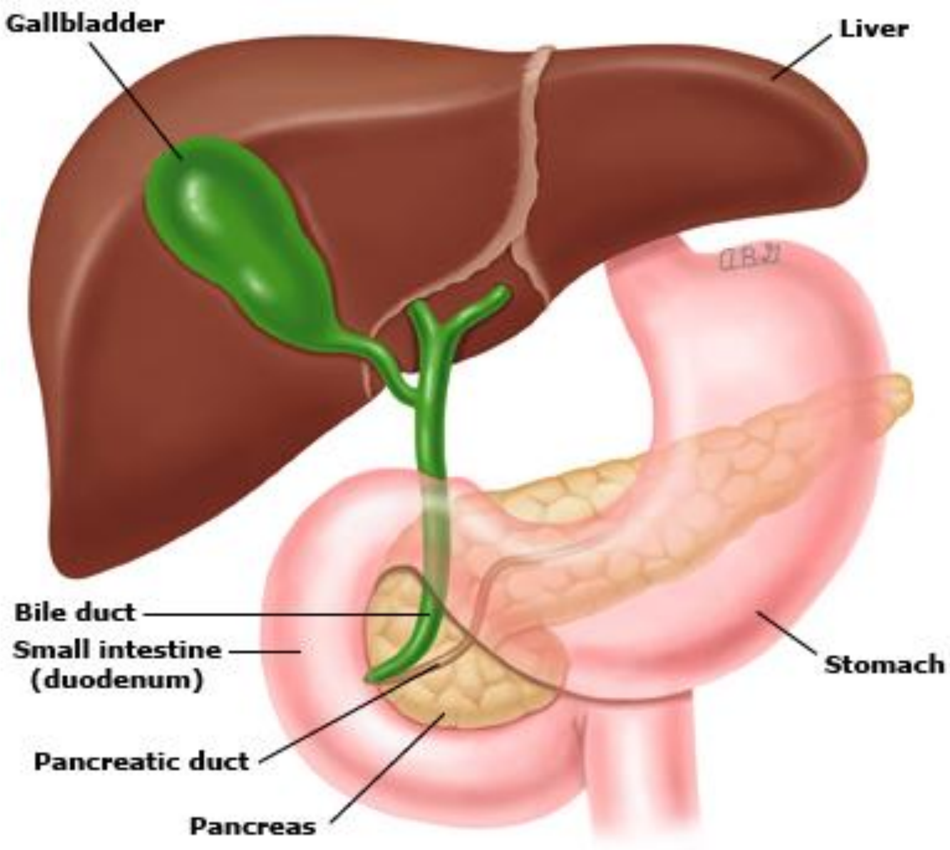

膵炎の発症機序

1. 膵臓内での消化酵素の早期活性化

膵臓内で早期にトリプシンなどの酵素が活性化され、膵臓自身を消化する自己消化が進行。
膵臓内カルシウム濃度の増加や、細胞内pHの低下が早期のトリプシン活性化を促進する。

↓

2. 膵臓の壊死と膵臓周囲の損傷

活性化されたトリプシンが膵臓内で他の酵素（ホスホリパーゼ、キモトリプシン、エラスターゼなど）を活性化し、膵臓の細胞を破壊していく。

↓

3. 炎症反応と全身への影響

膵臓から放出されたサイトカインや酵素が血液を介して全身に影響を与え、**ショック**や**腎不全**などの合併症を引き起こす

EBMグランプリ開始

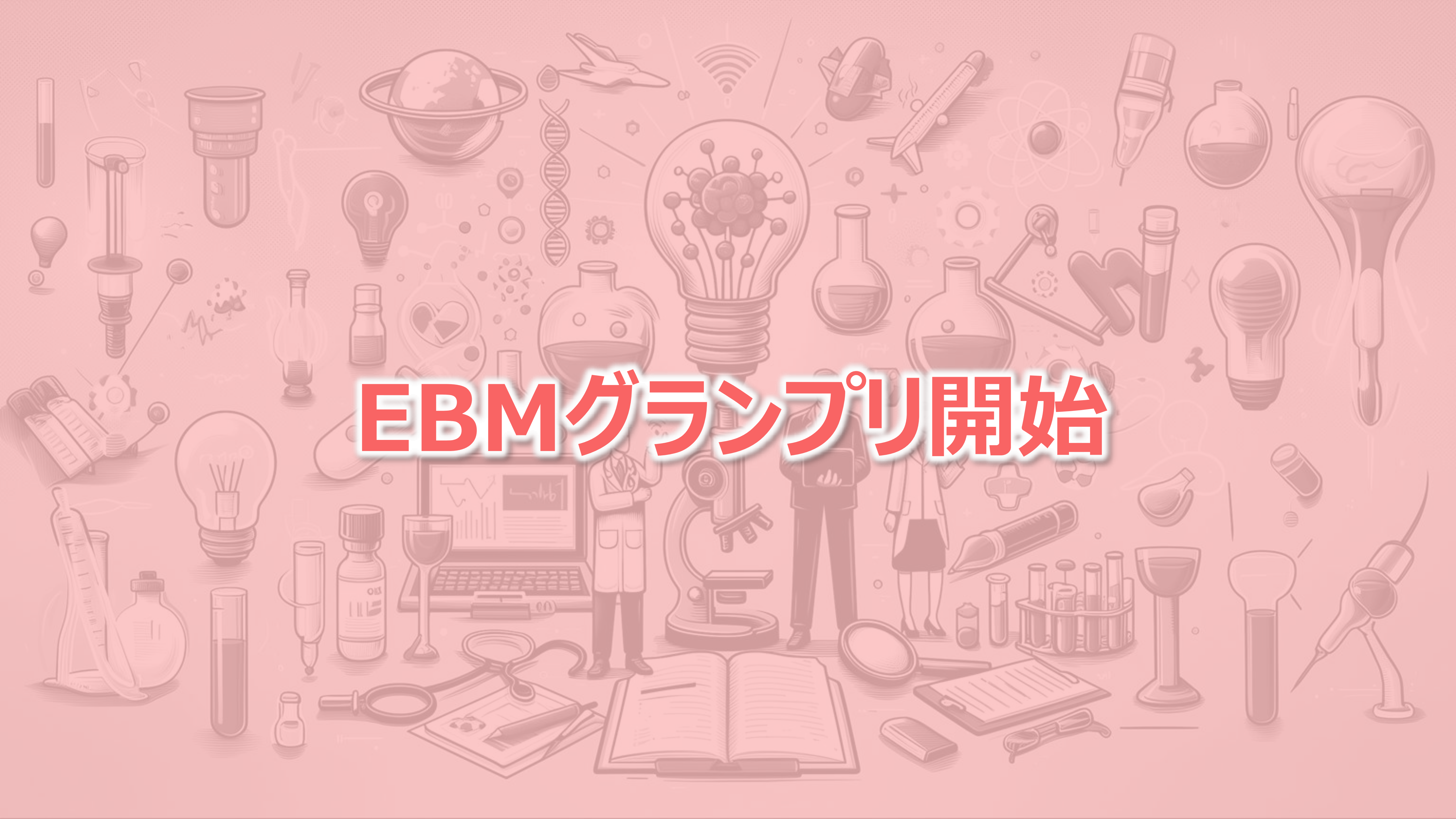

Q1 問題

Case 1 Q1 前半10 分

あなたは、この患者に対し、ERから入院後の管理を考えている。
エビデンスに基づいた輸液のプランについて, 下記の選択肢の中から
正しいものを全て選びなさい

a. 来院後4時間以内は、1.0L以上輸液する
b. 来院後24時間以内は、6.0L/日輸液する
c. 来院後24～48時間は、6.0L/日輸液する
d. 来院後48時間経過後は、6.0L/日輸液する
e. 大量輸液の合併症を考慮し乳酸リンゲル液ではなく
生理食塩水を選択する

Case 1 Q1 後半5 分

あなたは、この患者に対し、ERから入院後の管理を考えている。
エビデンスに基づいた輸液のプランについて，下記の選択肢の中から
正しいものを全て選びなさい

a.　来院後4時間以内は、1.0L以上輸液する
b.　来院後24時間以内は、6.0L/日輸液する
c.　来院後24～48時間は、6.0L/日輸液する
d.　来院後48時間経過後は、6.0L/日輸液する
e.　大量輸液の合併症を考慮し乳酸リンゲル液ではなく生理食塩水を選択する

Q1 解説

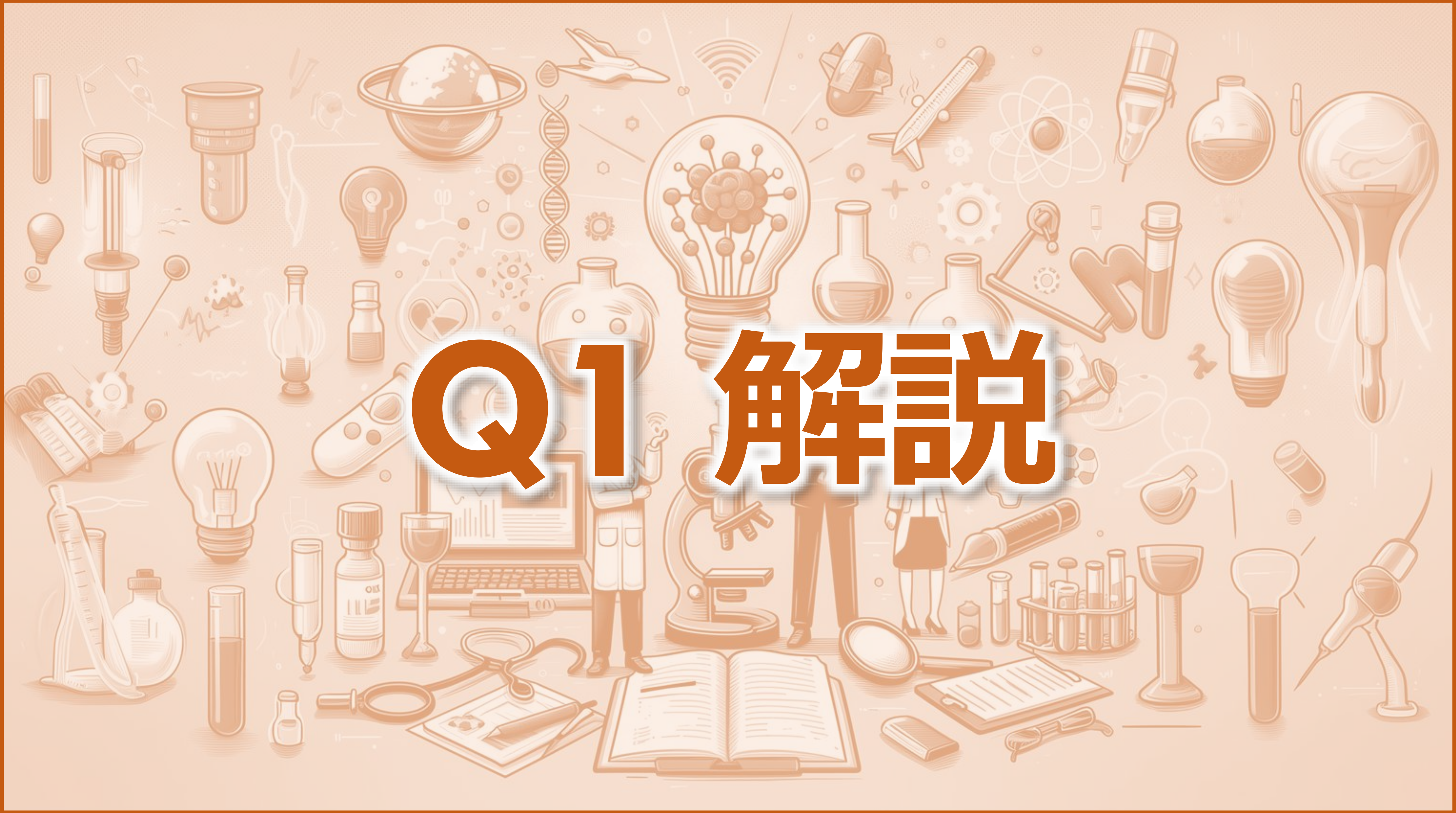

急性膵炎 診療ガイドライン 2021

急性膵炎診療ガイドライン 2021 改訂出版委員会 編

第5版

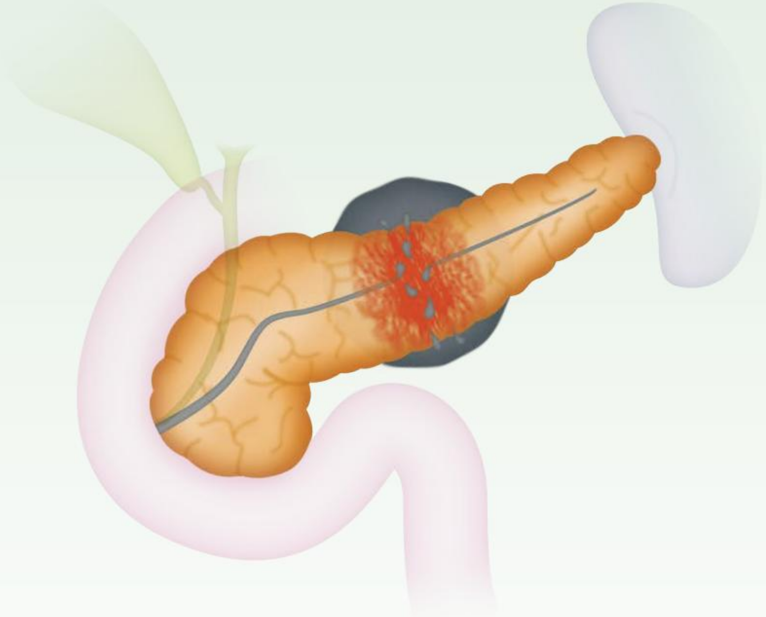

さらに近年，初期輸液の治療戦略において，来院後 4～6 時間以内の超早期の初期輸液がより重要であるとする意見があり，これは急性膵炎患者 1,000 例以上を対象とした観察研究で，来院後の初期 4 時間以内に 1 L 以上積極的に初期輸液を行うと，以降の集中治療介入の必要性が低くなり，24 時間以上（4.3 L 以上）積極的に初期輸液投与を行うと，局所合併症のリスクが高くなることに基づいている（OS）[9]。このような病院受診時の極初期から積極的輸液を開始する治療戦略について，今後さらなる検討が望まれる。

A. 致命率

Study or Subgroup	Rapid Events	Rapid Total	Standard Events	Standard Total	Weight	Risk Ratio M-H, Random, 95% CI	Risk of Bias A B C D E F G
Buxbaum2017	0	27	1	33	4.2%	0.40 [0.02, 9.55]	+ + - + + + +
Cuéllar-Monterrubio 2020	0	43	0	45		Not estimable	+ - - + ? ? +
Mao2009	11	36	4	40	23.6%	3.06 [1.07, 8.75]	- - - + ? ? ?
Mao2010	19	56	9	59	34.7%	2.22 [1.10, 4.50]	- - - + ? ? ?
Wang 2013	14	64	16	68	37.5%	0.93 [0.49, 1.75]	- - - + ? ? ?
Wu2011	0	19	0	21		Not estimable	+ + - + + + +
**Total (95% CI)**		**245**		**266**	**100.0%**	**1.61 [0.82, 3.15]**	
Total events	44		30				

Heterogeneity: Tau² = 0.21; Chi² = 5.83, df = 3 (P = 0.12); I² = 49%
Test for overall effect: Z = 1.39 (P = 0.17)

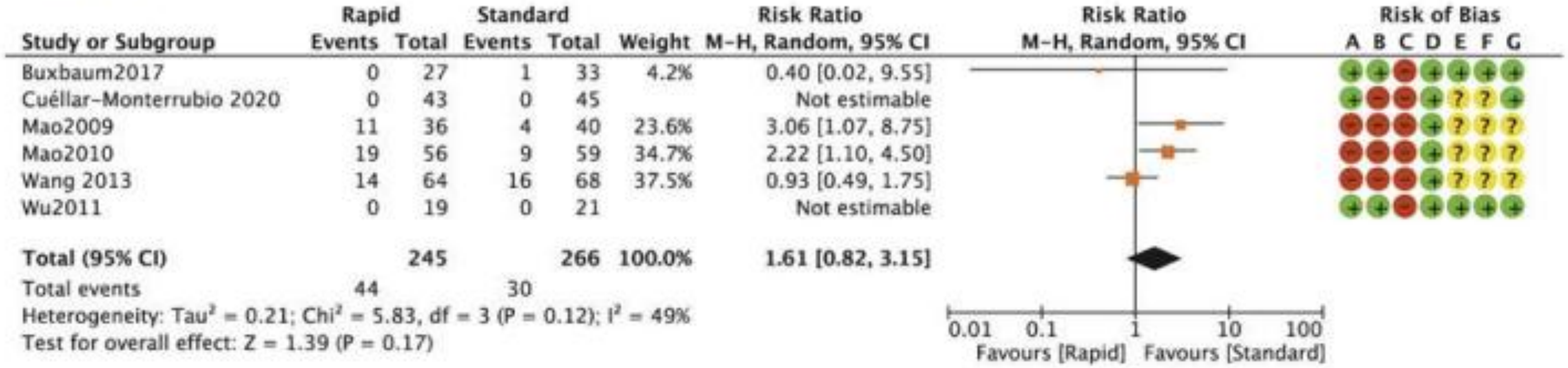

Risk of bias legend
(A) Random sequence generation (selection bias)
(B) Allocation concealment (selection bias)
(C) Blinding of participants and personnel (performance bias)
(D) Blinding of outcome assessment (detection bias): Mortality
(E) Incomplete outcome data (attrition bias)
(F) Selective reporting (reporting bias)
(G) Other bias

B. 膵壊死・感染性膵壊死・膵膿瘍発生率

Study or Subgroup	Rapid Events	Rapid Total	Standard Events	Standard Total	Weight	Risk Ratio M-H, Random, 95% CI	Risk of Bias A B C D E F G
Cuéllar-Monterrubio 2020	4	43	3	45	82.7%	1.40 [0.33, 5.87]	+ - - ? ? ? +
Wu2011	1	19	0	21	17.3%	3.30 [0.14, 76.46]	+ + - - + + +
**Total (95% CI)**		**62**		**66**	**100.0%**	**1.62 [0.44, 5.98]**	
Total events	5		3				

Heterogeneity: Tau² = 0.00; Chi² = 0.24, df = 1 (P = 0.62); I² = 0%
Test for overall effect: Z = 0.72 (P = 0.47)

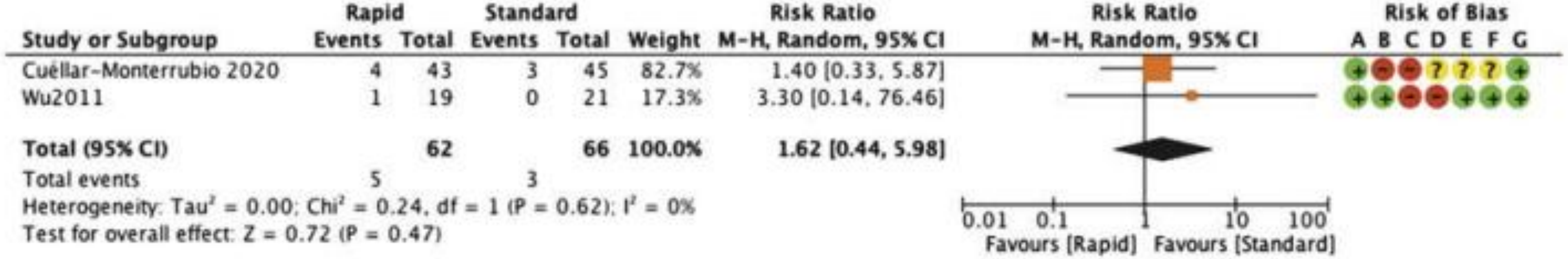

UNITED EUROPEAN GASTROENTEROLOGY
ueg journal

Original Article

United European Gastroenterology Journal
2017, Vol. 5(4) 491–498
© Author(s) 2016
Reprints and permissions:
sagepub.co.uk/journalsPermissions.nav
DOI: 10.1177/2050640616671077
journals.sagepub.com/home/ueg
SAGE

An international multicenter study of early intravenous fluid administration and outcome in acute pancreatitis

P：（Population, 患者群）

急性膵炎と診断された成人患者（初回または再発の急性膵炎）

I：(Intervention, 介入)

救急外来到着から4時間以内の早期輸液
（中等度の輸液療法: 500～1000mlまたはから積極的な輸液療法>1000ml）

C：（Comparison, 比較群）

非積極的輸液療法（<500mlの輸液量）

O：（Outcome, 結果）

■ 主なアウトカム：
- 局所合併症の発生率
- 侵襲的治療の必要性
- 死亡率

■ 副次的アウトカム：
- 入院期間の短縮

救急外来での輸液量（<500 ml、500–1000 ml、>1000 ml）による患者のアウトカム

FVER	Local complications	Persistent organ failure	Invasive treatment	Death
<500 ml				
Cases	51/269 (19%)	19/269 (7.1%)	13/269 (4.8%)	8/269 (3%)
OR (95% CI)	*1*	*1*	*1*	*1*
ORa (95% CI)	*1*	*1*	*1*	*1*
500–1000 ml				
Cases	48/427 (11.2%)	19/427 (4.4%)	7/427 (1.6%)	7/427 (1.6%)
OR (95% CI)	0.54 (0.35–0.83)[a]	0.61 (0.32–1.18)	0.33 (0.13–0.83)[a]	0.54 (0.2–1.52)
ORa (95% CI)	0.67 (0.43–1.05)	0.56 (0.28–1.14)	0.37 (0.14–0.98)[b]	0.46 (0.15–1.38)
>1000 ml				
Cases	50/314 (15.9%)	15/314 (4.8%)	5/314 (1.6%)	8/314 (2.5%)
OR (95% CI)	0.81 (0.53–1.24)	0.66 (0.33–1.33)	0.32 (0.11–0.91)[b]	0.85 (0.32–2.3)
ORa (95% CI)	1.15 (0.71–1.86)	0.5 (0.22–1.12)	0.21 (0.05–0.84)[a]	0.64 (0.20–2)

1000mL以上の輸液で侵襲的治療のリスクが最も低かった, 臓器障害や死亡率で有意差はなし

入院後24時間以内の輸液量（<3200 ml、3200–4300 ml、>4300 ml）による患者アウトカム

FV24	Local complications	Persistent organ failure	Invasive treatment	Death
<3200 ml				
Cases	30/260 (11.5%)	11/260 (4.2%)	2/260 (0.8%)	4/260 (1.5%)
OR (95% CI)	*1*	*1*	*1*	*1*
ORa (95% CI)	*1*	*1*	*1*	*1*
3200–4300 ml				
Cases	41/249 (16.5%)	9/249 (3.6%)	6/249 (2.4%)	3/249 (1.2%)
OR (95% CI)	1.51 (0.91–2.51)	0.85 (0.35–2.09)	3.19 (0.64–15.93)	0.78 (0.17–3.52)
ORa (95% CI)	1.28 (0.76–2.16)	0.85 (0.34–2.15)	2.62 (0.52–13.36)	0.83 (0.18–3.92)
>4300 ml				
Cases	65/256 (25.4%)	16/256 (6.2%)	13/256 (5.1%)	5/256 (2%)
OR (95% CI)	2.61 (1.63–4.19)[a]	1.51 (0.69–3.32)	6.9 (1.54–30.9)[a]	1.28 (0.338–4.8)
ORa (95% CI)	1.91 (1.17–3.14)[a]	1.57 (0.67–3.72)	4.5 (0.99–21.07)	1.78 (0.43–7.46)

4300mL以上の輸液で局所合併症のリスクが高い, 臓器障害や死亡率で有意差はなし

RESEARCH SUMMARY

Aggressive or Moderate Fluid Resuscitation in Acute Pancreatitis

- 対象（P, Population）：急性膵炎患者
- 介入（I, Intervention）：積極的輸液療法：（20mL/kgのボーラス投与後、3mL/kg/時の維持投与）
- 比較（C, Comparison）：中等度輸液療法：Volume正常時はボーラスなし
  Volume低下時は10mL/kgのボーラス→1.5mL/kg/時の維持投与
- 結果（O, Outcome）：主要評価項目：入院中に中等度または重症の急性膵炎に進行した割合。
  安全性評価項目：輸液過剰（fluid overload）の発生率。
  副次的評価項目：入院期間、局所合併症、臓器不全、栄養サポート、ICU入室。

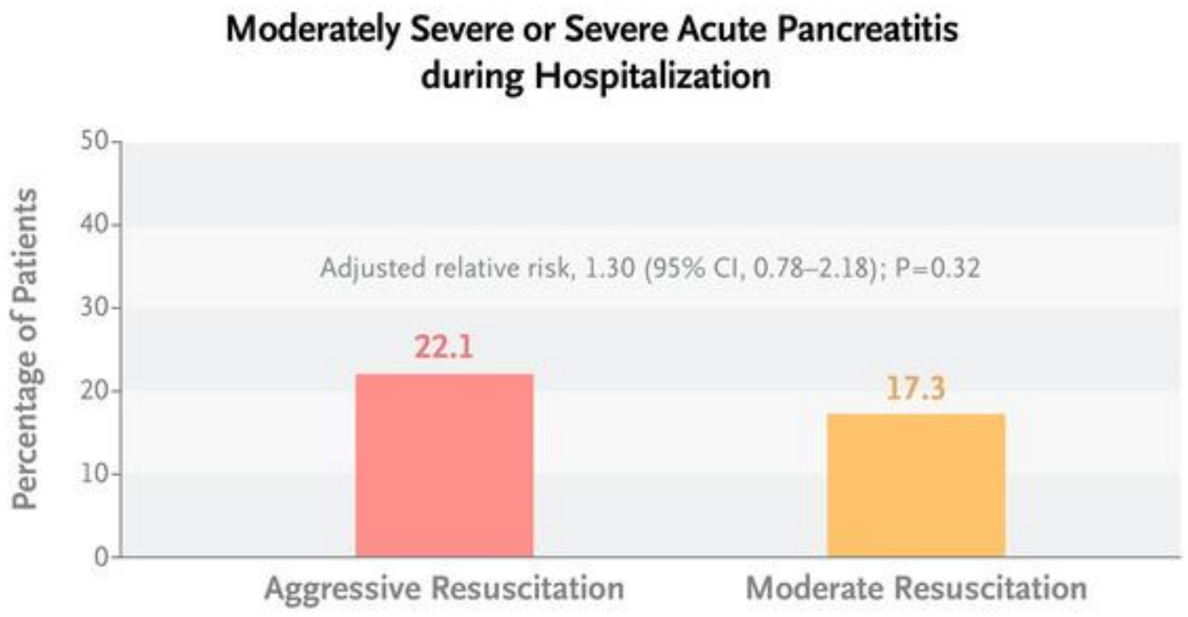

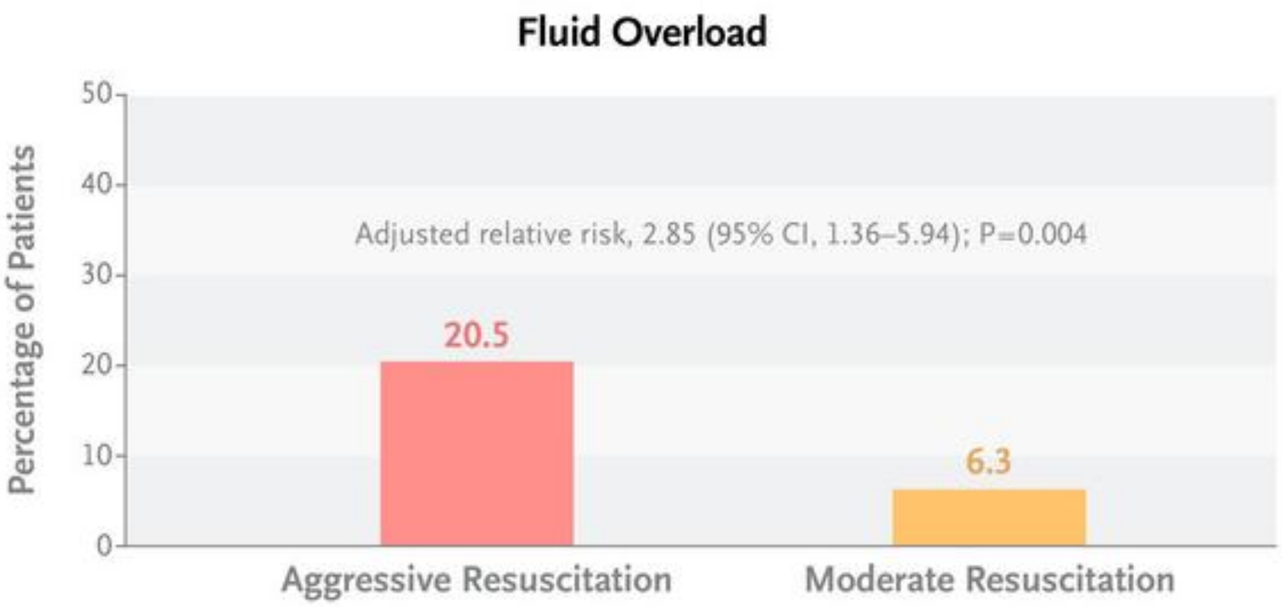

Management of acute pancreatitis

Despite numerous studies that have evaluated intravenous hydration, the timing, the rate, the type of fluid, the duration of treatment, and the appropriate goal to direct such intravenous hydration is unclear [15]. While previous guidelines have recommended 250 to 500 mL/hour of normal saline during the first 24 to 48 hours (5 to 10 mL per kilogram of body weight per hour), evidence from randomized controlled trials suggest that a lower volume may be used in patients with mild acute pancreatitis [1,2,16,17]. In a randomized trial, 249 patients with acute pancreatitis were randomly assigned to aggressive fluid resuscitation (lactated Ringer's solution bolus of 20 mL/kg followed by 3 mL/kg/hour) versus moderate resuscitation (1.5 mL/kg/hour with a 10 mL/kg bolus only in patients with hypovolemia) [17]. In both groups, initial physical assessment was performed at three hours to evaluate for fluid overload, and goal-directed resuscitation was adjusted 12, 24, 48, and 72 hours based on volume status. Although there was no difference in the incidence of moderately severe or severe pancreatitis or duration of hospitalization between the two groups, the trial was terminated early due to higher rates of fluid overload in the aggressive resuscitation group (20 versus 6 percent).

While previous guidelines have recommended 250 to 500 mL/hour of normal saline during the first 24 to 48 hours (5 to 10 mL per kilogram of body weight per hour), evidence from randomized controlled trials suggest that a lower volume may be used in patients with mild acute pancreatitis.

[illegible]

Fluid resuscitation with lactated Ringer's solution can reduce the incidence of systemic inflammatory response syndrome (SIRS) as compared with normal saline [5,18,21-23]. Use of lactated Ringer's solution in patients with acute pancreatitis may reduce hospital stay and intensive care unit admissions, however, further studies are needed [23]. In rare patients with acute pancreatitis due to hypercalcemia, lactated Ringer's is contraindicated because it contains 3 mEq/L calcium. In these patients, normal saline should be used for volume resuscitation. Several other approaches to fluid replacement have also been reported in patients with acute pancreatitis [24,25]. The use of hydroxyethyl starch containing fluids should be avoided given the absence of demonstrable mortality benefit and possible risk of multiple organ failure [3,26]. (See "Intraoperative fluid management", section on 'Hydroxyethyl starches'.)

Fluid resuscitation is important in the first 24 to 48 hours after onset of the disease. Continued aggressive fluid resuscitation after 48 hours may not be advisable as overly vigorous fluid resuscitation is associated with an increased need for intubation and increased risk of abdominal compartment syndrome

Fluid resuscitation with lactated Ringer's solution can reduce the incidence of systemic inflammatory response syndrome (SIRS) as compared with normal saline. Use of lactated Ringer's solution in patients with acute pancreatitis may reduce hospital stay and intensive care unit admissions, however, further studies are needed.

Case 1 Q1

あなたは、この患者に対し、ERから入院後の管理を考えている。
エビデンスに基づいた輸液のプランについて，下記の選択肢の中から正しいものを全て選びなさい

a. 来院後4時間以内は、1.0L以上輸液する
b. 来院後24時間以内は、6.0L/日輸液する
c. 来院後24～48時間は、6.0L/日輸液する
d. 来院後48時間経過後は、6.0L/日輸液する
e. 大量輸液の合併症を考慮し乳酸リンゲル液ではなく生理食塩水を選択する

Q2 問題

Case 1 Q2 前半10分

あなたは、この患者に対し、ERから入院後の管理を考えている。
エビデンスに基づいた抗菌薬・抗真菌薬のプランについて
下記の選択肢の中から正しいものを全て選びなさい

a. 抗菌薬は投与しない
b. メロペネムを投与する
c. セフメタゾールを投与する
d. フルコナゾールを投与する
e. 膵周囲に感染を疑う液体貯留があれば、
速やかにドレナージを行う

Case 1 Q2 後半5分

あなたは、この患者に対し、ERから入院後の管理を考えている。
エビデンスに基づいた抗菌薬・抗真菌薬のプランについて
下記の選択肢の中から正しいものを全て選びなさい

a. 抗菌薬は投与しない
b. メロペネムを投与する
c. セフメタゾールを投与する
d. フルコナゾールを投与する
e. 膵周囲に感染を疑う液体貯留があれば、
速やかにドレナージを行う

Q2 解説

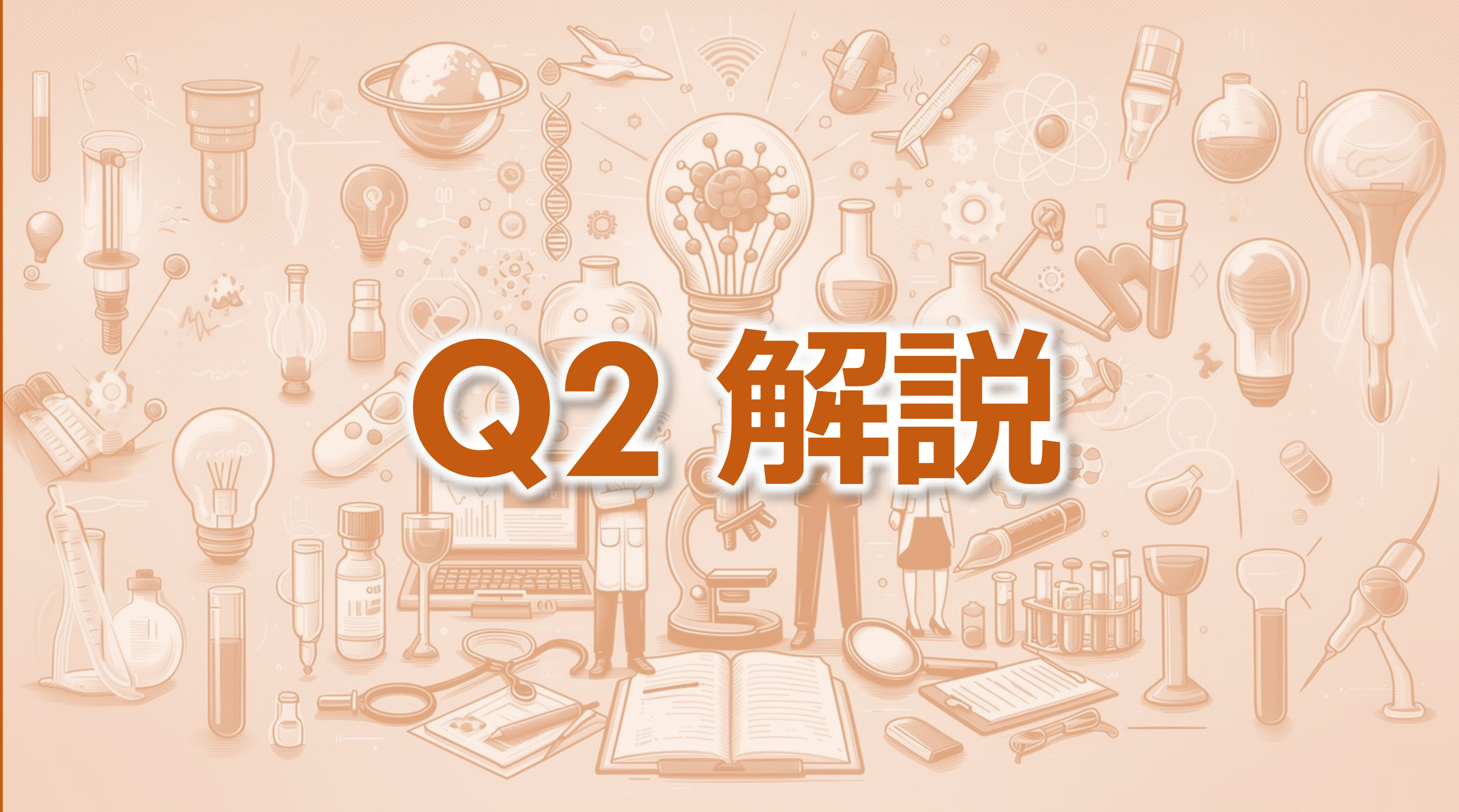

Up to 20 percent of patients with acute pancreatitis develop an extra-pancreatic infection .

Prophylactic antibiotics are not recommended in patients with acute pancreatitis, regardless of the type or disease severity.

In patients with predicted severe AP and necrotizing pancreatitis, the AGA suggests against the use of prophylactic antibiotics.

AGA Guideline on initial managemet of Acute Pancreatitis

軽症の急性膵炎に対して予防的抗菌薬の投与は行わない事を推奨する。重症急性膵炎または壊死性膵炎に対する予防的抗菌薬投与の生命予後に対する明らかな改善効果は証明されていない

急性膵炎診療ガイドライン 2021

In stable patients with infected necrosis, we attempt to delay drainage/necrosectomy by continuing antibiotics for at least four weeks .

感染性膵壊死に対しては，保存的治療で全身状態 が保たれていれば,被包化が起こる時期(通常発症4週以降)に内視鏡的もしくは経皮的ドレナージを行う。

急性膵炎診療ガイドライン 2021

Administration of prophylactic antifungal therapy (eg, fluconazole) along with prophylactic or therapeutic antibiotics is not recommended.

Case 1 Q2

あなたは、この患者に対し、ERから入院後の管理を考えている。
エビデンスに基づいた抗菌薬・抗真菌薬のプランについて
下記の選択肢の中から正しいものを全て選びなさい

a. 抗菌薬は投与しない
b. メロペネムを投与する
c. セフメタゾールを投与する
d. フルコナゾールを投与する
e. 膵周囲に感染を疑う液体貯留があれば、
   速やかにドレナージを行う

Q3 問題

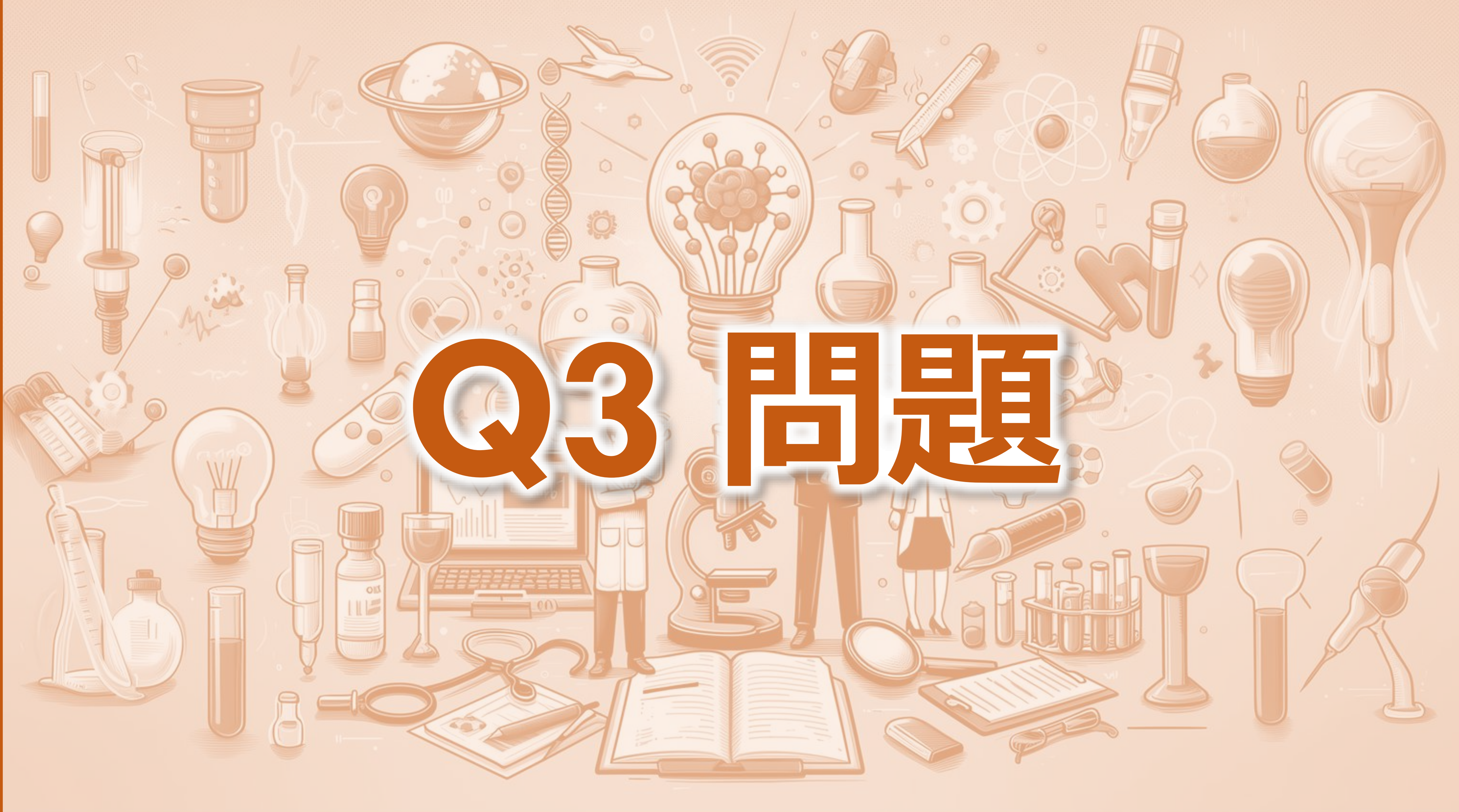

Case 1 Q3 前半10 分

あなたは、この患者に対し、ERから入院後の管理を考えている。エビデンスに基づいた治療のプランについて下記の選択肢の中から正しいものを全て選びなさい

a. フェンタニルを投与する
b. オクトレオチドを投与する
c. ナファモスタットを投与する
d. エソメプラゾールを投与する
e. ペントキシフィリンを投与する

Case 1 Q3 後半5分

あなたは、この患者に対し、ERから入院後の管理を考えている。
エビデンスに基づいた治療のプランについて下記の選択肢の中から正しいものを全て選びなさい

a. フェンタニルを投与する
b. オクトレオチドを投与する
c. ナファモスタットを投与する
d. エソメプラゾールを投与する
e. ペントキシフィリンを投与する

Q3 解説

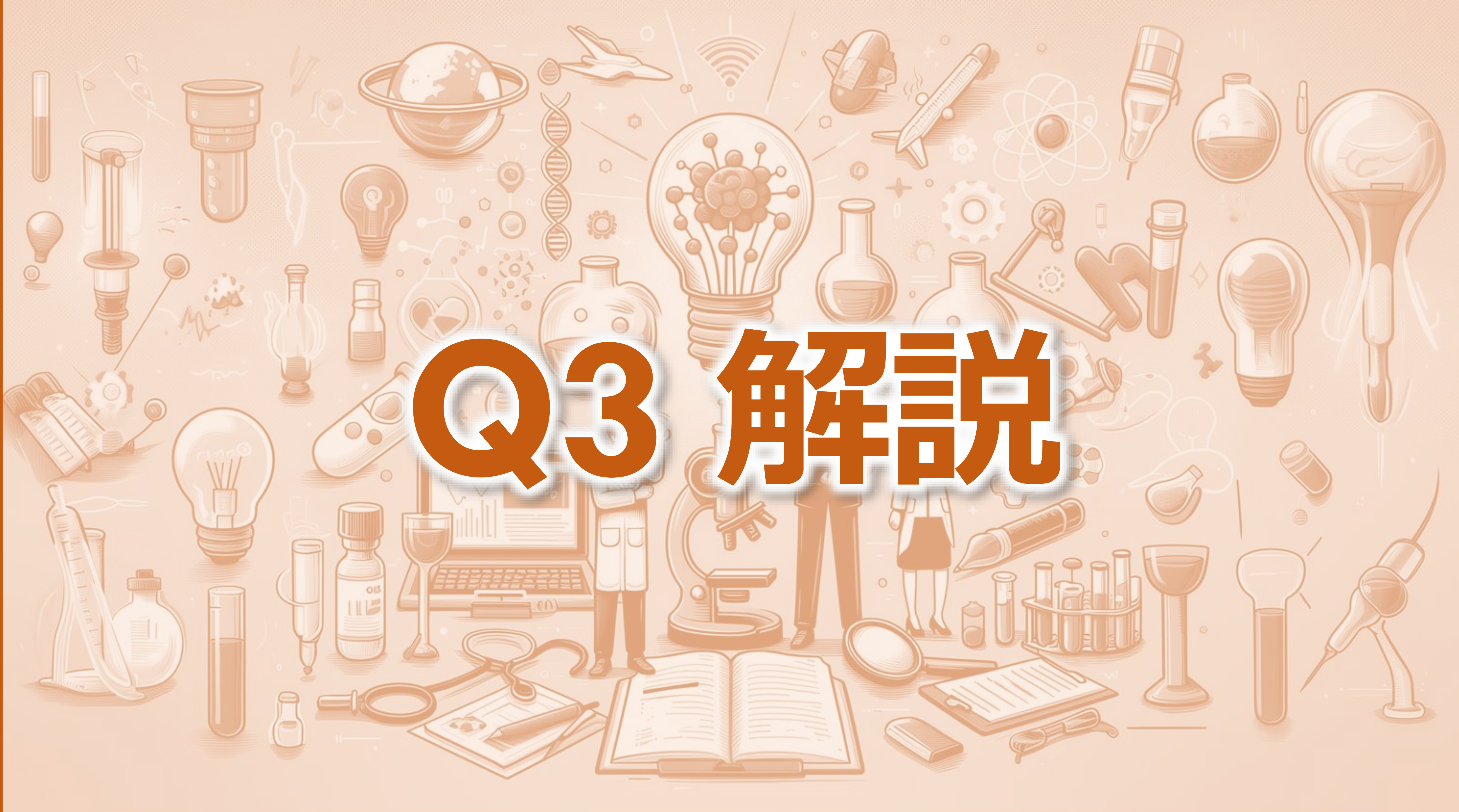

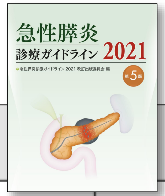

4. 薬物療法				
BQ15　急性膵炎に対する鎮痛薬はどのように使用するのか？	急性膵炎に対しては迅速に鎮痛薬を使用する。アセトアミノフェン，NSAIDs，ペンタゾシンなどの非オピオイドの投与を行い，その後，疼痛の程度に応じてオピオイドの使用も考慮する。	—	—	93

Opioids are safe and effective at providing pain control in patients with acute pancreatitis.

Other therapies with no role

- Pentoxifylline
- Antifungals
- Protease inhibitors

**4. 薬物療法**				
**BQ15　急性膵炎に対する鎮痛薬はどのように使用するのか？**	急性膵炎に対しては迅速に鎮痛薬を使用する。アセトアミノフェン，NSAIDs，ペンタゾシンなどの非オピオイドの投与を行い，その後，疼痛の程度に応じてオピオイドの使用も考慮する。	―	―	93
**CQ13　予防的抗菌薬は急性膵炎の予後改善に有用か？**	軽症の急性膵炎に対して予防的抗菌薬の投与は行わないことを推奨する。	強い推奨	高	94
	重症急性膵炎または壊死性膵炎に対する予防的抗菌薬投与の，生命予後や感染性膵合併症発生に対する明らかな改善効果は証明されていない。	なし	中	
**CQ14　蛋白分解酵素阻害薬は急性膵炎の病態改善に有用か？**	急性膵炎において蛋白分解酵素阻害薬の生命予後や合併症発生に対する明らかな改善効果は証明されていない。	なし	中	97
**CQ15　急性膵炎において胃酸分泌抑制薬の投与は有用か？**	急性膵炎において胃酸分泌抑制薬の投与を行わないことを提案する。	弱い推奨	低	98
**4. ERCP 後膵炎の予防**				
**CQ35　一時的膵管ステント留置はERCP 後膵炎の予防に有用か？**	ERCP 後膵炎高危険群に対する一時的膵管ステント留置はERCP 後膵炎予防に有用である。	弱い推奨	高	175
**CQ36　直腸内NSAIDs投与はERCP後膵炎の発症抑制に有用か？**	ERCP 後膵炎のリスクを有する場合，禁忌事項のない限りNSAIDs（インドメタシンもしくはジクロフェナク）をERCP 前もしくは後に直腸内投与することはERCP 後膵炎の発症抑制に有用である。	弱い推奨	高	178

Meta-Analysis > *Sci Rep* (IF: 4.38; Q1). 2023 Feb 16;13(1):2791.

No evidence for the benefit of PPIs in the treatment of acute pancreatitis: a systematic review and meta-analysis

1. 膵仮性嚢胞の形成率:
   - PPI群では形成率が61%低下
   (OR 0.39; 95%CI 0.18–0.87)
2. ARDS（急性呼吸窮迫症候群）:
   - 両群間で有意差なし
   (OR 0.56; 95%CI 0.04–8.59)
3. 消化管出血リスク:
   - PPI群でリスクが増加
   (OR 1.81; 95%CI 1.41–2.33)
   - 背景因子（既往歴や併用薬）による可能性あり。
4. 入院期間:
   - 両群間で統計的有意差なし
   (MD -3.47日; 95%CI -12.32～5.39)
5. 7日間死亡率:
   - 両群間で有意差なし
   (OR 0.77; 95%CI 0.05–10.65)

Case 1 Q3

あなたは、この患者に対しERから入院後の管理を考えている。
エビデンスに基づいた他の治療のプランについて
下記の選択肢の中から正しいものを全て選びなさい

a. フェンタニルを投与する
b. オクトレオチドを投与する
c. ナファモスタットを投与する
d. エソメプラゾールを投与する
e. ペントキシフィリンを投与する

Q4 問題

Case 1 Q4 前半10 分

あなたは、この患者に対しERから入院後の管理を考えている。
エビデンスに基づいた食事のプランについて
下記の選択肢の中から正しいものを全て選びなさい

a. 入院48時間以内に経腸栄養を行う
b. 入院48時間以内に中心静脈栄養を行う
c. 経口摂取開始時に低カロリー食を選択する
d. 経口摂取開始時に低蛋白・低脂肪食を選択する
e. 腹痛や膵アミラーゼが改善するまで
腸管安静(絶食)とする

Case 1 Q4 後半5分

あなたは、この患者に対しERから入院後の管理を考えている。
エビデンスに基づいた食事のプランについて
下記の選択肢の中から正しいものを全て選びなさい

a. 入院48時間以内に経腸栄養を行う
b. 入院48時間以内に中心静脈栄養を行う
c. 経口摂取開始時に低カロリー食を選択する
d. 経口摂取開始時に低蛋白・低脂肪食を選択する
e. 腹痛や膵アミラーゼが改善するまで
腸管安静(絶食)とする

Q4 解説

急性膵炎Bunddle

急性膵炎診断時

□予後因子スコアを用いて重症度を繰り返し評価する
□(発症48時間以内)重症度に応じたモニタリングを実施する
□初期には積極的な輸液療法を実施する
□適切な期間：疼痛のコントロールを行う
□軽症急性膵炎では、予防的抗菌薬は使用しない

診断から3時間以内

□病歴、検査所見から膵炎の成因を鑑別する
□造影CT Gradeで重症度判定
□重症急性膵炎では、適切な施設への搬送を検討

診断から24時間以内

□予後因子スコアを用いて重症度を評価
□胆石性膵炎：胆管炎合併、横断など胆道通過障害があれば、早期のERCP+ESTを検討

診断から48時間以内

□予後因子スコアを用いて重症度を評価
□重症急性膵炎では、診断後48時間以内に経腸栄養を少量から開始する

診断から24～48時間以内

□予後因子スコアを用いて重症度を繰り返し評価

診断から48時間以内の画像評価は
必須ではないが，重症度評価は
繰り返し行うことが望ましい。

BQ/CQ/FRQ	推奨文	推奨度	エビデンスの確実性	頁
5. 栄養療法				
BQ16 急性膵炎における栄養の意義と至適投与経路は何か？	重症例における栄養は，全身性炎症反応により必要量が増加したエネルギーを補給する意味に加えて，経腸栄養は感染予防策として重要であり，重篤な腸管合併症のない重症例には経腸栄養を行う。	―	―	99
CQ16 重症急性膵炎に対する経腸栄養の至適開始時期はいつか？	経腸栄養は発症早期に開始すれば，合併症発生率を低下させ生存率の向上に寄与するので，入院後48時間以内に少量からでも開始する。	強い推奨	高	102
CQ17 経腸栄養ではどこから何を投与するか？	経腸栄養の経路としては，空腸に限らず十二指腸や胃に栄養剤を投与してもよい。	弱い推奨	中	104
CQ18 軽症膵炎ではどのように食事を再開するか？	軽症膵炎では腸蠕動が回復すれば，経口摂取を再開することができる。	弱い推奨	高	107

早期経口摂取は急性膵炎患者において入院期間を短縮(SMD: -1.01, P<0.001)し、医療費削減（SMD: -0.83, P<0.001）にも効果がある一方で、死亡率、痛みの再発率、膵炎の進行率については統計的に有意な差が認められなかった.

Pancreatology Vol.22, Issue 2, March 2022, 175-184

Early refeeding (≤48 after hospitalization), as compared with delayed refeeding, did not increase adverse effects or exacerbate symptoms .

Parenteral nutrition should be initiated only in patients who do not tolerate enteral feeding or if the target rate of enteral feeding is not achieved within 48 to 72 hours, as the use of parenteral nutrition as an adjunct to enteral feeding may be harmful .

Immediate Oral Refeeding in Patients With Mild and Moderate Acute Pancreatitis　A Multicenter, Randomized Controlled Trial (PADI trial)

Ann Surg. 2021 Aug 1;274(2):255-263.

- P: 軽度および中等度の急性膵炎の患者。
- I: 入院後すぐに低脂肪の固形食を開始する即時経口再栄養（IORF）
- C: 臨床, 検査値の改善後に徐々に食事を再開する従来の経口再栄養（CORF）
- O: 入院期間、副次的アウトカム：痛みの再発、食事の不耐性、合併症、入院費用

Outcomes	IORF Group n = 71	CORF Group n = 60	*P* value
Length of hospital stay, days, mean (SD)	3.4 (1.7)	8.8 (7.9)	<0.001
Days from admission to refeeding, days, mean (SD)	0	2.8 (1.7)	<0.001
Days from refeeding to discharge, days, mean (SD)	3.4 (1.7)	5.4 (4.8)	<0.001
Need for opioids or analgesia infusion	0	5 (8.3)	<0.001
Intolerance diet n (%)	1 (1.4)	13 (21.6)	<0.001
Reasons for intolerance			
Relapse of pain, n (%)	0	10 (16.7)	<0.001
Nausea and vomiting, n (%)	1 (1.4)	2 (3.3)	0.37
Anorexy, n (%)	0	1 (1.6)	0.44
Progression of acute pancreatitis, n (%)	0	6 (10.0)	<0.006
Complications, n (%)	3 (4.2)	11 (18.3)	<0.009

軽度および中等度の急性膵炎患者における即時経口再栄養が、入院期間と入院費用を有意に減少させ、合併症のリスクを増加させることなく実施可能

Case 1 Q4

あなたは、この患者に対しERから入院後の管理を考えている。
エビデンスに基づいた食事のプランについて
下記の選択肢の中から正しいものを全て選びなさい

a. 入院48時間以内に経腸栄養を行う
b. 入院48時間以内に中心静脈栄養を行う
c. 経口摂取開始時に低カロリー食を選択する
d. 経口摂取開始時に低蛋白・低脂肪食を選択する
e. 腹痛や膵アミラーゼが改善するまで
 腸管安静(絶食)とする

Q5 問題

Case 1 Q5 前半10分

あなたは、この患者に対しERから入院後の管理を考えている。
エビデンスに基づいた入院後のルーチンの検査について
下記の選択肢の中から正しいものを全て選びなさい

a. 尿量を測定する
b. 膀胱内圧を測定する
c. 24時間以内に血液検査を行う
d. 24〜48時間以内に血液検査を行う
e. 48時間以内に造影CTを再撮影する

Case 1 Q5 後半5分

あなたは、この患者に対しERから入院後の管理を考えている。
エビデンスに基づいた入院後のルーチンの検査について
下記の選択肢の中から正しいものを全て選びなさい

a.　尿量を測定する
b.　膀胱内圧を測定する
c.　24時間以内に血液検査を行う
d.　24～48時間以内に血液検査を行う
e.　48時間以内に造影CTを再撮影する

Q5 解説

急性膵炎Bunddle

急性膵炎診断時

□予後因子スコアを用いて重症度を繰り返し評価する
□(発症48時間以内)重症度に応じたモニタリングを実施する
□初期には積極的な輸液療法を実施する
□適切な期間：疼痛のコントロールを行う
□軽症急性膵炎では、予防的抗菌薬は使用しない

診断から3時間以内

□病歴、検査所見から膵炎の成因を鑑別する
□造影CT Gradeで重症度判定
□重症急性膵炎では、適切な施設への搬送を検討

診断から24時間以内

□予後因子スコアを用いて重症度を評価
□胆石性膵炎：胆管炎合併、横断など胆道通過障害があれば、早期のERCP+ESTを検討

診断から48時間以内

□予後因子スコアを用いて重症度を評価
□重症急性膵炎では、診断後48時間以内に経腸栄養を少量から開始する

診断から24～48時間以内

□予後因子スコアを用いて重症度を繰り返し評価

診断から48時間以内の画像評価は
必須ではないが, 重症度評価は
繰り返し行うことが望ましい。

★POINT★ 急性膵炎の予後因子(各1点)

・予後因子3点未満で死亡率1.4%,3点以上で18%.

①	Base Excess≦-3 mEq/Lまたはショック(sBP≦80 mmHg)
②	PaO2≦60 mmHg(RA)または呼吸不全(人工呼吸器管理)
③	BUN≧40 mg/dL(またはCr≧2 mg/dL)または乏尿(輸液後も尿量400mL/day以下)
④	LDH≧基準値上限の2倍
⑤	Plt≦10万/mm^3
⑥	総Ca≦7.5 mg/dL
⑦	CRP≧15 mg/dL
⑧	SIRS項目陽性数≧3: ①体温＞38℃ or ＜36℃ ②HR>90 ③RR>20 or PaCO2<32 Torr<br>④WBC>12,000/mm^3 or <4,000/mm^3 or 10%幼若球出現
⑨	年齢≧70歳

★POINT★ 造影CTによるGrade分類

①炎症の膵外進展度

0点	**前腎傍腔**	前腎筋膜・後腎筋膜の肥厚, 前腎傍腔の液体貯留, 周囲脂肪織濃度上昇
1点	**結腸間膜根部**	膵臓腹側の結腸間膜根部を超えて炎症が横行結腸間膜に波及
2点	**腎下極以遠**	腎臓の鼻息で脂肪織濃度上昇, 液体貯留, 筋膜肥厚

②膵の造影不良域: 頭部, 体部, 尾部に分けて判定

0点	各区域に限局または膵の周辺のみ
1点	2つの区域にかかる
2点	2つの区域全体を占める, またはそれ以上

①+②の合計点数によってCT Gradeが決まる.
(Grade1: 1点以下, Grade2: 2点, Grade3: 3点以上)

CT Grade分類		①膵外進展度		
		前腎傍腔	結腸間膜根部	腎下極以遠
②膵造影不良域	＜1/3	Grade 1	Grade 1	**Grade 2**
	1/3-1/2	Grade 1	**Grade 2**	**Grade 3**
	1/2＜	**Grade 2**	**Grade 3**	**Grade 3**

Case 1 Q5

あなたは、この患者に対しERから入院後の管理を考えている。
エビデンスに基づいた入院後のルーチンの検査について
下記の選択肢の中から正しいものを全て選びなさい

a. 尿量を測定する
b. 膀胱内圧を測定する
c. 24時間以内に血液検査を行う
d. 24～48時間以内に血液検査を行う
e. 48時間以内に造影CTを再撮影する

Case 2

Case 2

【症例】74歳男性
【主訴】動悸、呼吸困難、倦怠感

【現病歴】 2023年7月X日夕より倦怠感が出現、体温を測定すると37.8℃であったが病院を受診せず自宅で経過を見ていた。その後、夜にかけて39℃まで体温が上昇し動悸、呼吸困難が出現するも, 病院嫌いから受診はしなかった。翌7月X+1日になっても症状の改善に乏しく呼吸困難の増悪を認めた事から同居の妻が救急要請、当院に搬送となった.

【既往歴】 血圧が高いと言われた事がある, 数年前から労作時の呼吸困難を自覚
【生活歴】 喫煙：past smoker、飲酒：チューハイ２本/日
【常用薬】 なし

【Vital Signs】 E1V2M5, 体温39.2度、血圧190/108mmHg、心拍数 140回/分 不整、呼吸数 30回/分、SpO2 92% (リザーバー付マスク12L/分)
【身体診察】聴診で呼吸音減弱、crackles聴取なし、右下腿に腫脹・発赤・疼痛あり

身体所見

右足首に発赤,腫脹・圧痛・熱感あり→ 蜂窩織炎 と診断した

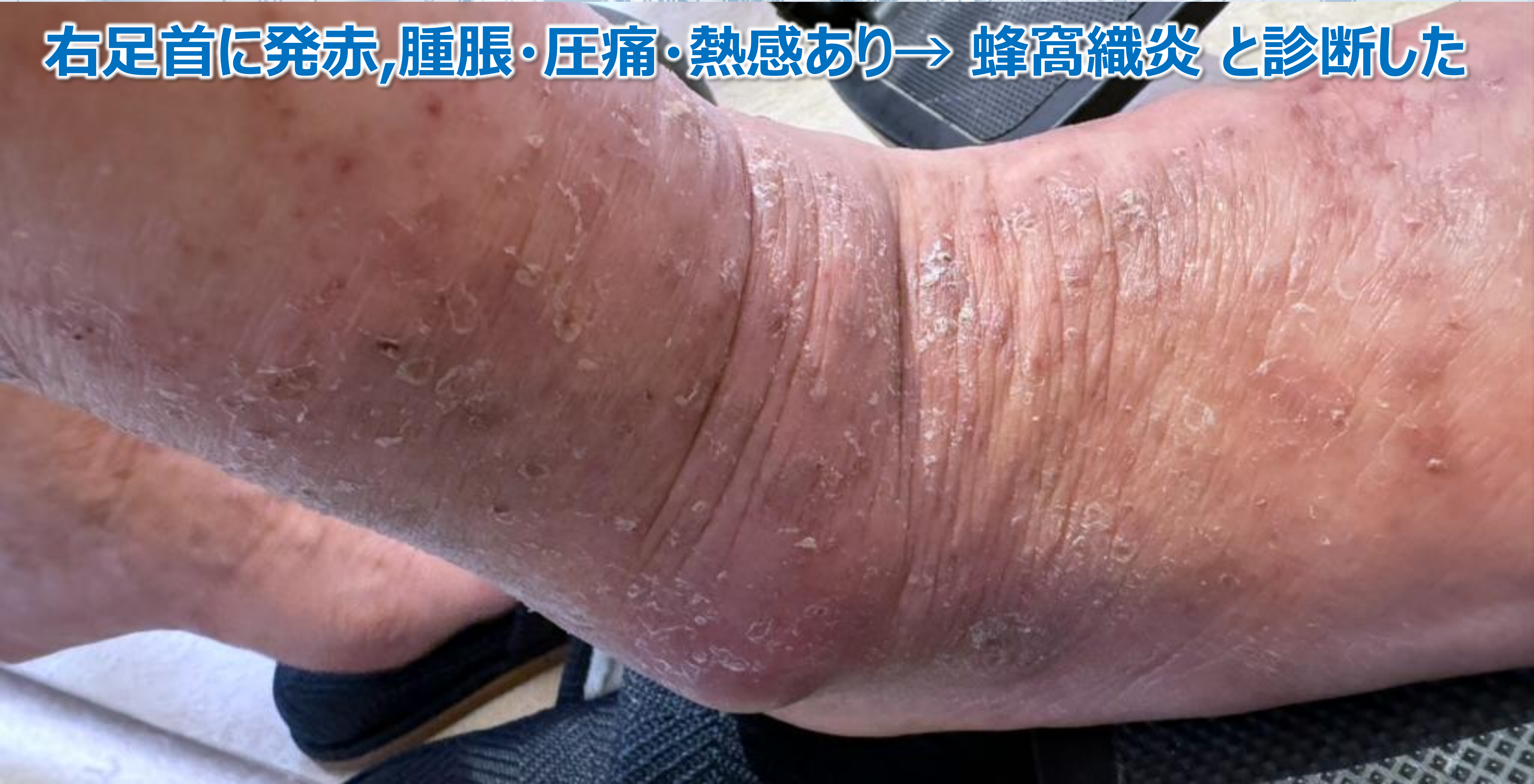

血液・尿検査

**血算**			ALP	450	IU/L	CRP	28.24	mg/dL	蛋白	-
WBC	19,000	/μL	γ-GTP	23	IU/L	NT-pro BNP	1240	pg/mL	糖	-
Hb	12.4	g/dL	T-Bil	1.0		Troponin T	0.02	ng/mL	ケトン体	-
Plt	8.4	10^4/μL	Na	121	mEq/L	PCT	8.4	mg/dL	細菌	-
**凝固**			K	4.8	mEq/L	Glucose	120	mg/dL	**血液ガス**	
INR	1.36		Cl	90	mEq/L	HbA1c	6.3	mg/dL	FiO2	0.24
APTT	45.1	sec	TP	6.5	g/dL	**尿検査**			pH	7.31
D-dimer	0.7	μg/mL	Alb	3.6	g/dL	外観	clear		PaO_2	84
**生化学**			BUN	27.3	mg/dL	比重	1.012		$PaCO_2$	39
AST	83	IU/L	Cr	1.49	mg/dL	pH	6.5		HCO_3	19.4
ALT	77	IU/L	CK	890	IU/L	赤血球	-		BE	-6.6
LDH	470	IU/L	CK-MB	6	IU/L	白血球	-		Lac	3.3

血液・尿検査

**血算**			ALP	450	IU/L	CRP	28.24	mg/dL	蛋白	-
WBC	19,000	/μL	γ-GTP	23	IU/L	NT-pro BNP	1240	pg/mL	糖	-
Hb	12.4	g/dL	T-Bil	1.0		Troponin T	0.02	ng/mL	ケトン体	-
Plt	8.4	10^4/μL	Na	121	mEq/L	PCT	8.4	mg/dL	細菌	-
**凝固**			K	4.8	mEq/L	Glucose	120	mg/dL	**血液ガス**	
INR	1.36		Cl	90	mEq/L	HbA1c	6.3	mg/dL	FiO2	0.24
APTT	45.1	sec	TP	6.5	g/dL	**尿検査**			pH	7.31
D-dimer	0.7	μg/mL	Alb	3.6	g/dL	外観	clear		PaO_2	84
**生化学**			BUN	27.3	mg/dL	比重	1.012		$PaCO_2$	39
AST	83	IU/L	Cr	1.49	mg/dL	pH	6.5		HCO_3	19.4
ALT	77	IU/L	CK	890	IU/L	赤血球	-		BE	-6.6
LDH	470	IU/L	CK-MB	6	IU/L	白血球	-		Lac	3.3

心電図

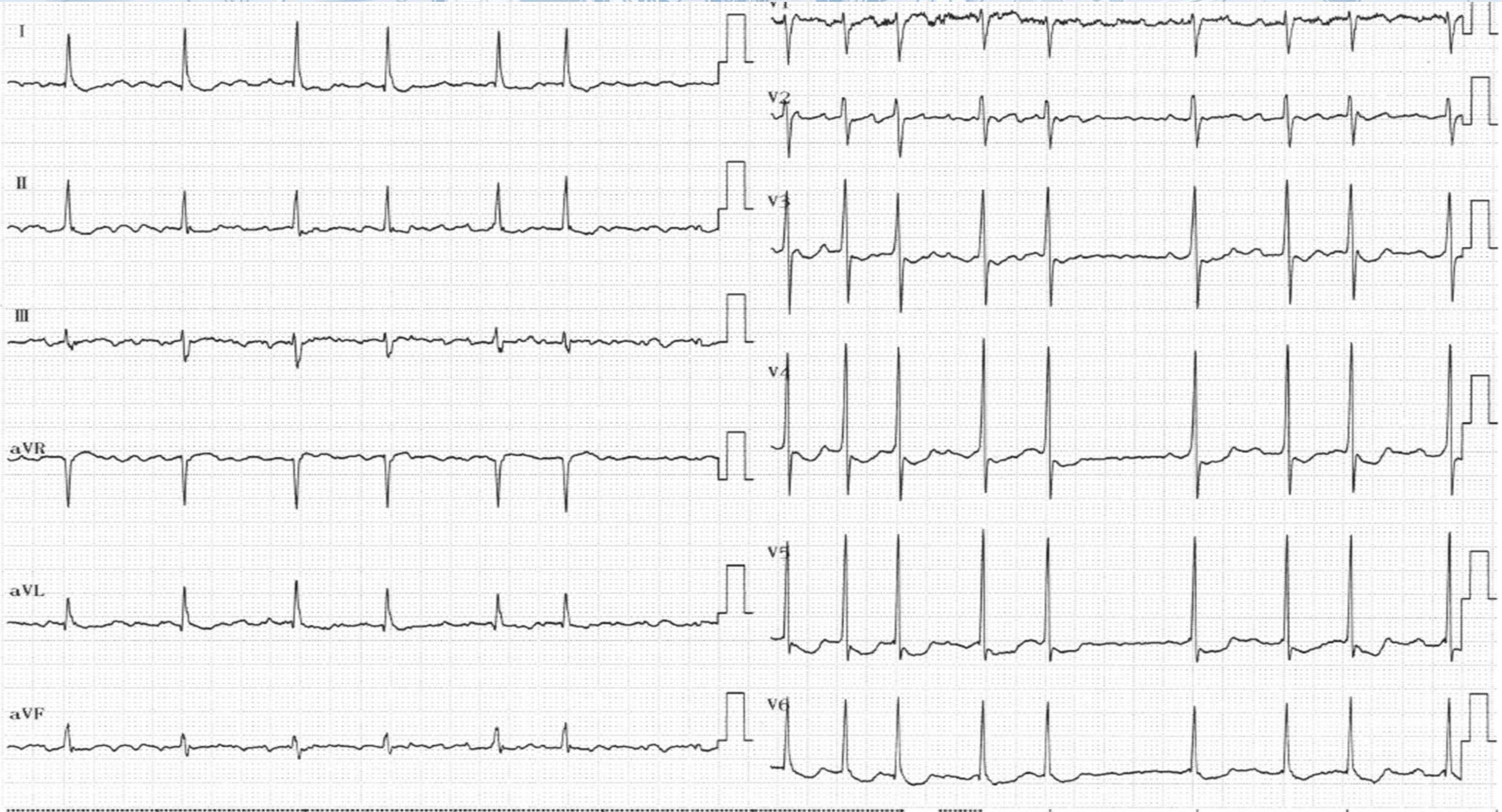

胸部単純CT検査

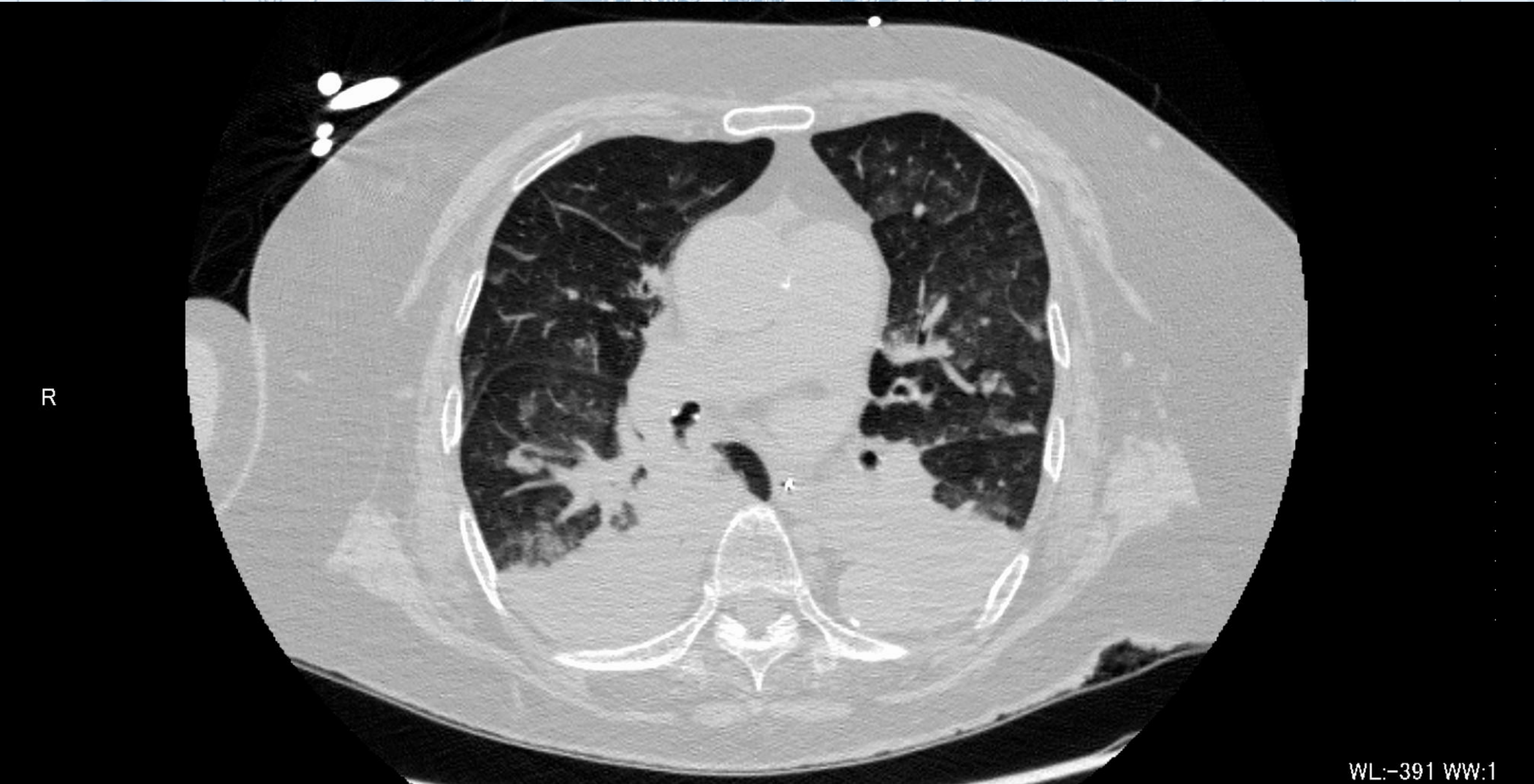

胸部単純CT検査

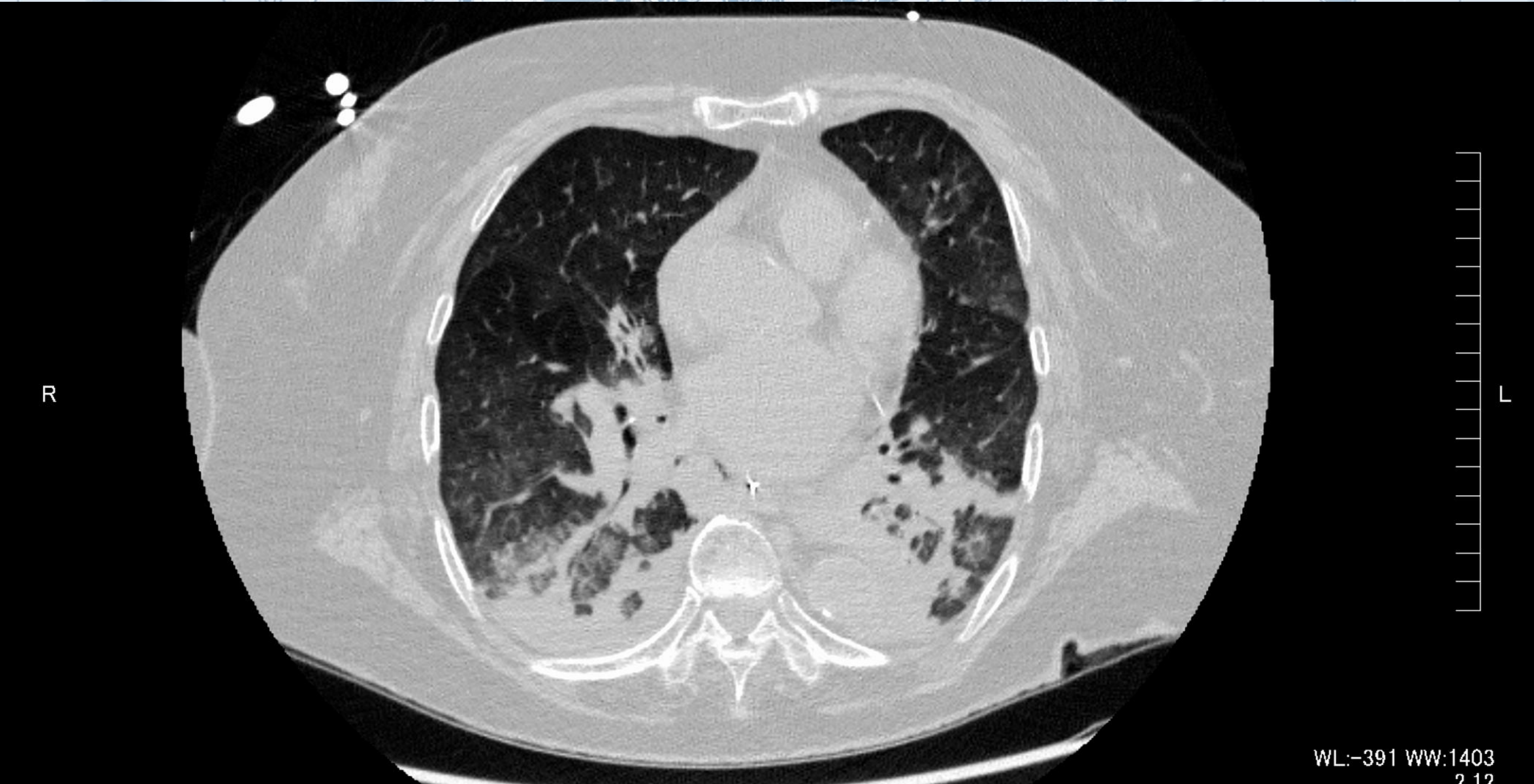

胸部単純CT検査

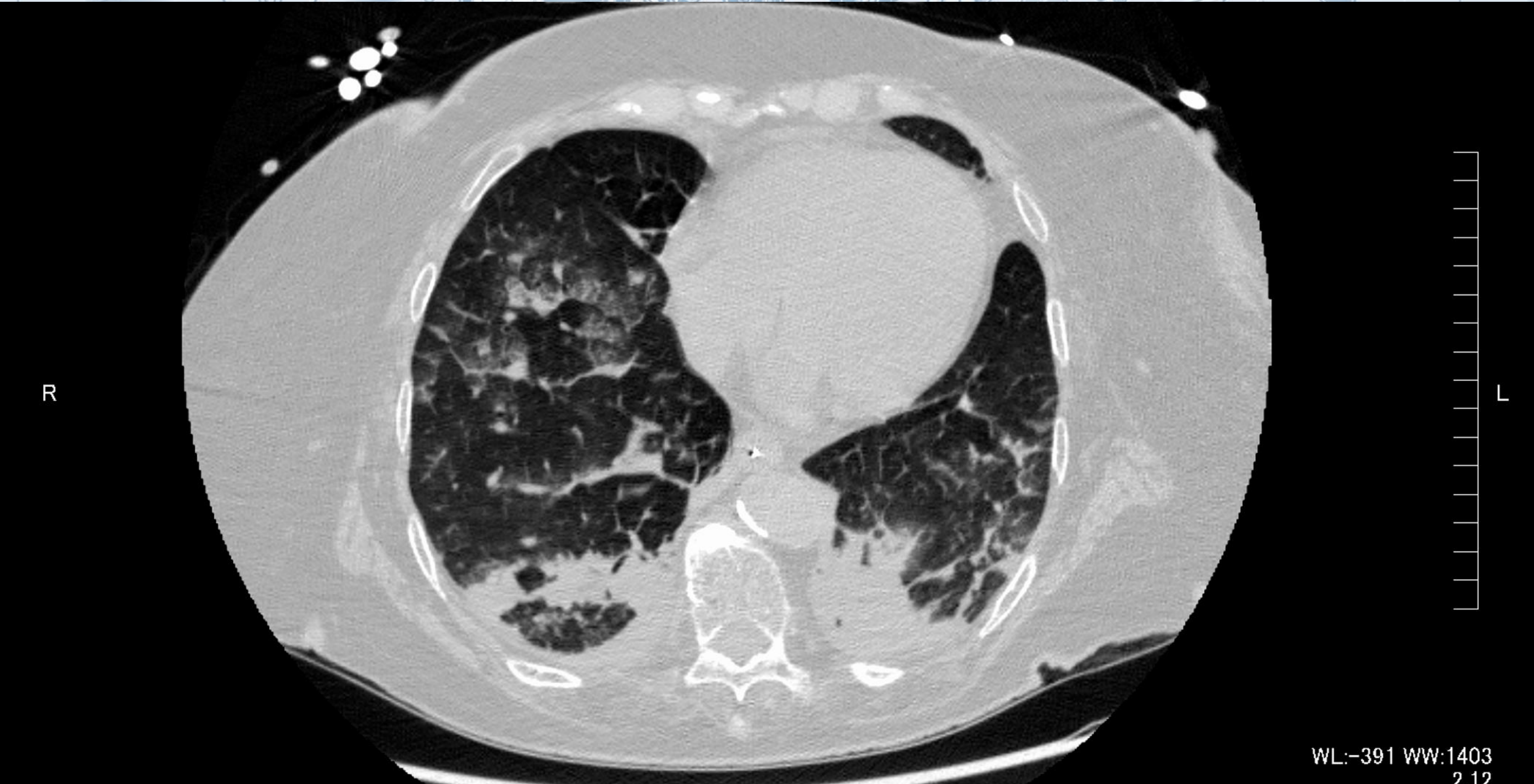

胸部単純CT検査

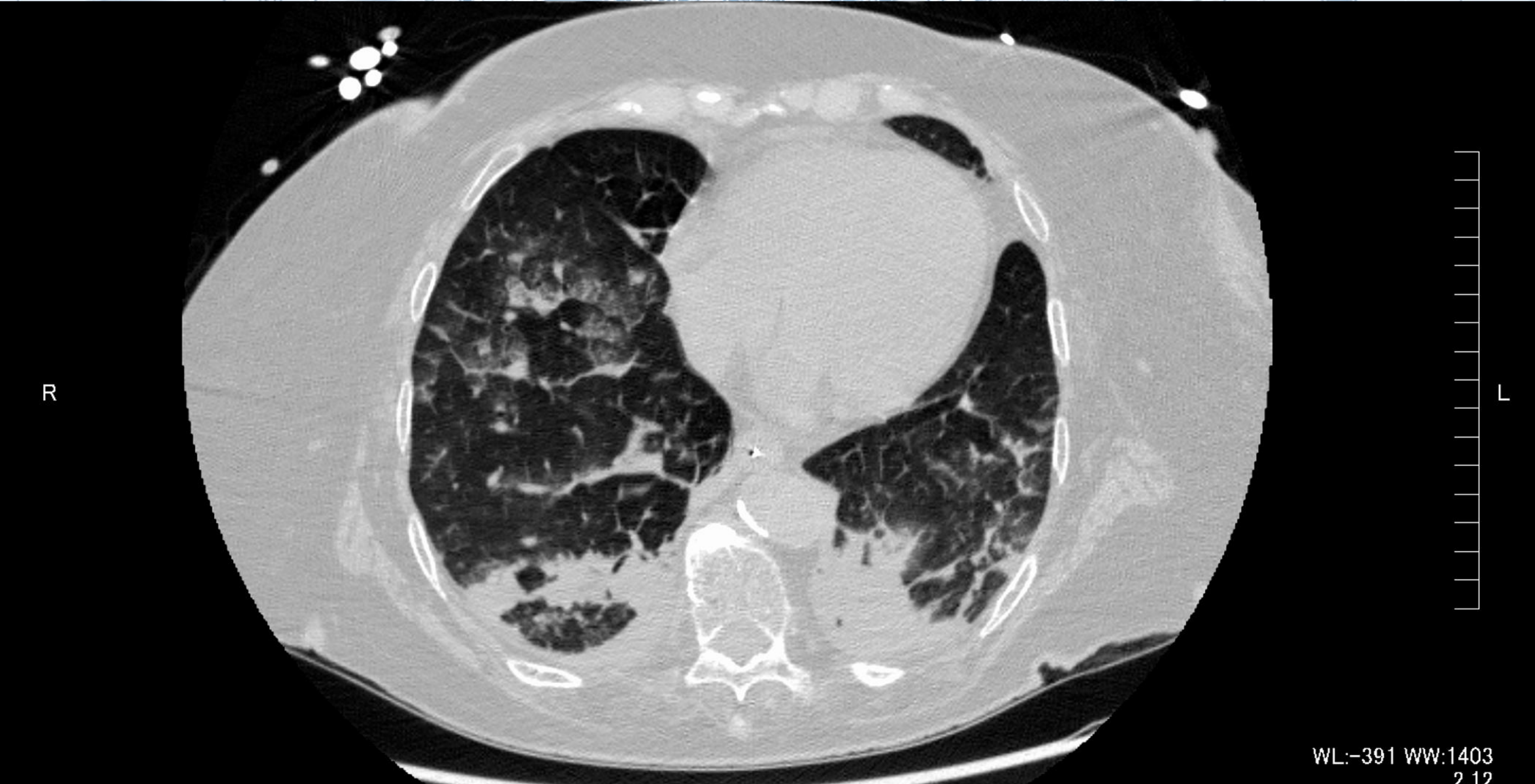

心臓超音波検査

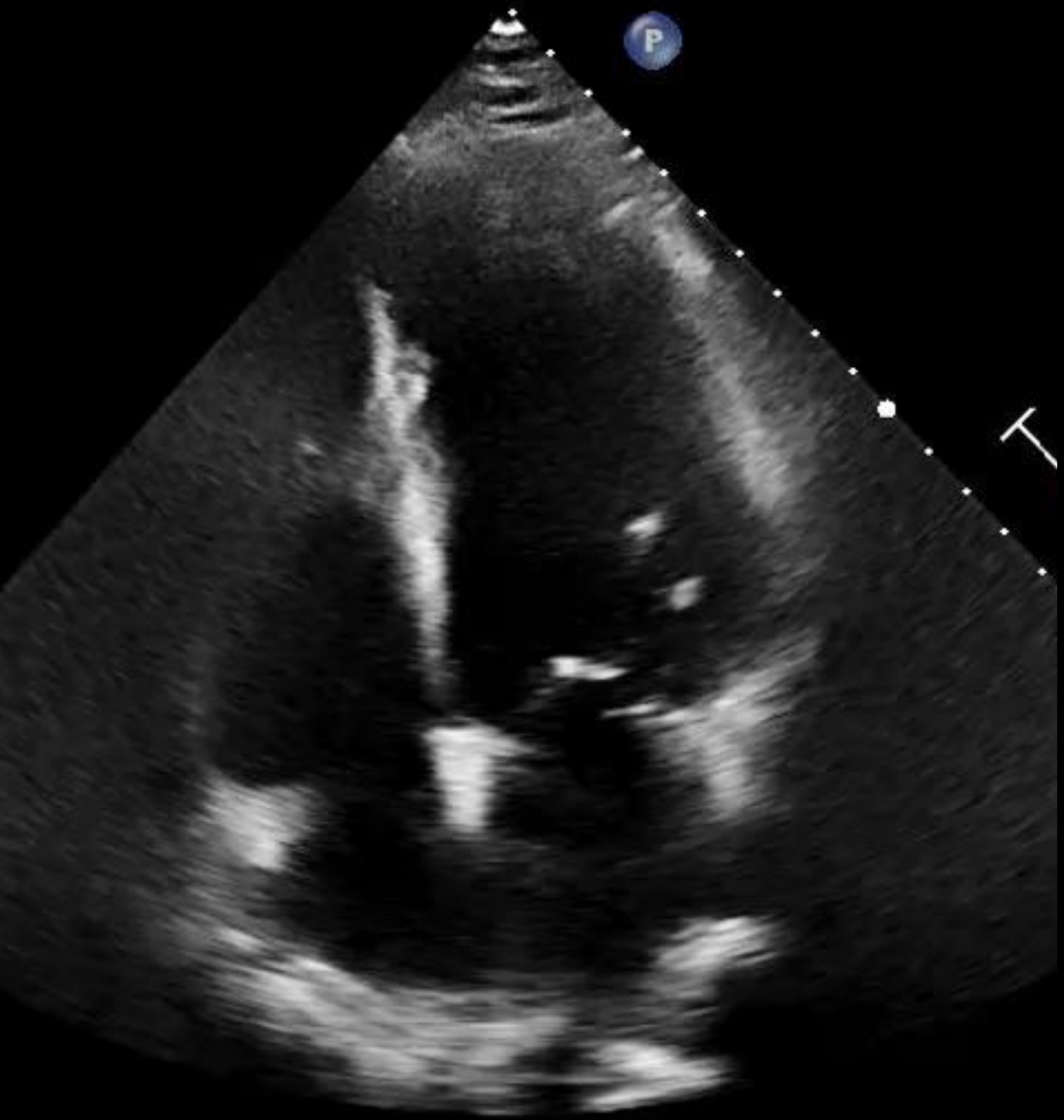

EF 10-20%,
mild TR
TRPG ：36mmHg
TAPSE 16mm
Diffuse-
hypokinesis
心嚢液貯留なし

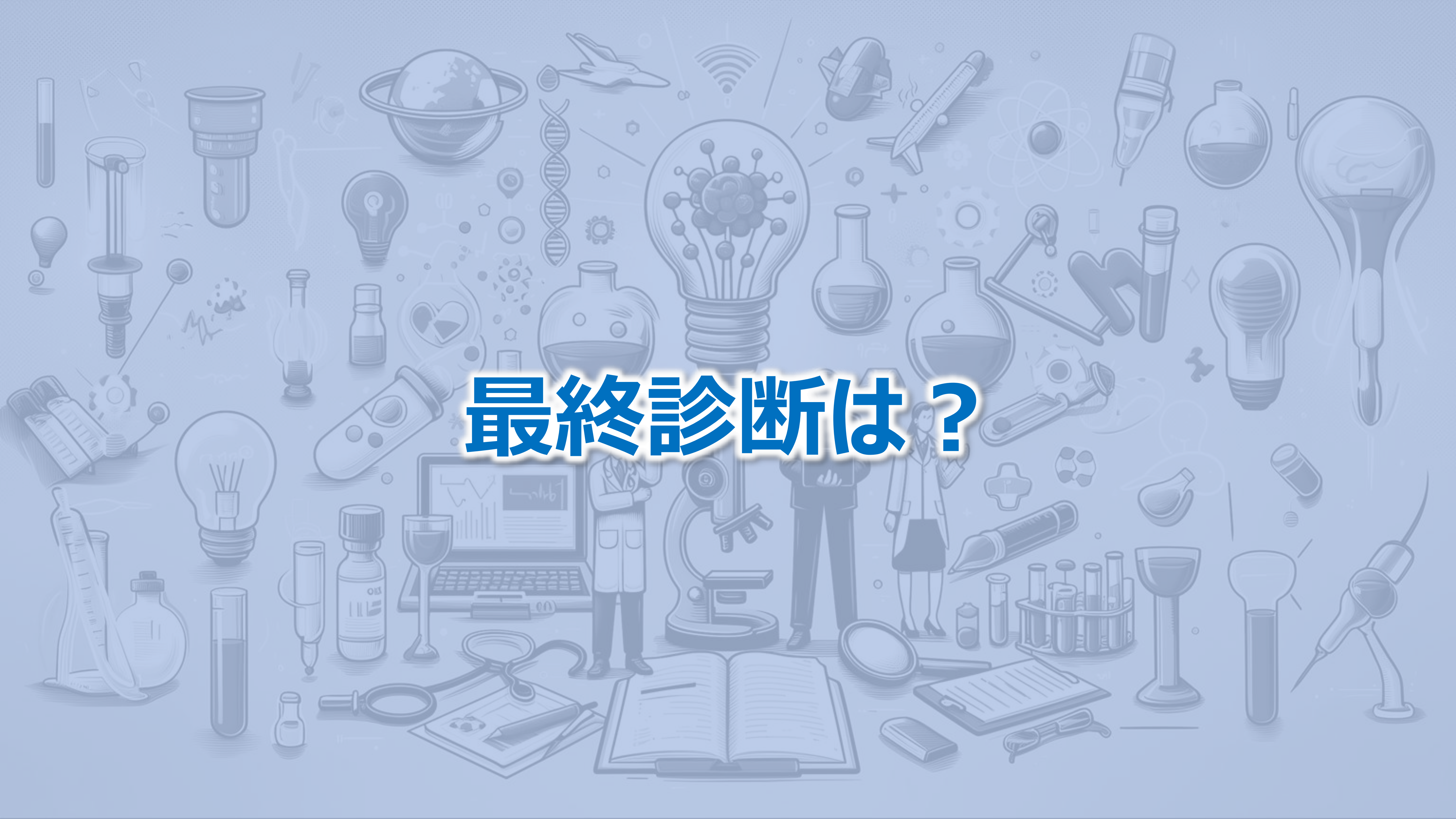

最終診断は？

What is 心不全？

「なんらかの心臓機能障害，すなわち心臓に器質的および/あるいは機能的異常が生じて，心ポンプ機能の代償機転が破綻した結果，呼吸困難・倦怠感や浮腫が出現し，それに伴い運動耐容能が低下する臨床症候群」

フラミンガム研究における心不全の診断基準

大基準	大または小基準	小基準
発作性夜間呼吸困難	治療に反応して5日間で4.5kg以上の体重減少（心不全治療による効果なら大基準1つ，それ以外ならば小基準1つとみなす）	下腿浮腫
頸静脈怒張		夜間咳嗽
肺ラ音		労作性呼吸困難
胸部X線での心拡大		肝腫大
急性肺水腫		胸水貯留
拡張早期性ギャロップ（III音）		肺活量減少（最大量の1/3以下）
中心静脈圧上昇（> 16 cmH_2O）		頻脈（≧120bpm）
循環時間延長（25 秒以上）		
肝・頸静脈逆流		
（剖検での肺水腫，内臓うっ血や心拡大）		

うっ血による自覚症状と身体所見		
左心不全	自覚症状	呼吸困難，息切れ，頻呼吸，起座呼吸
	身体所見	水泡音，喘鳴，ピンク色泡沫状痰，III音やIV音の聴取
右心不全	自覚症状	右季肋部痛，食思不振，腹満感，心窩部不快感
	身体所見	肝腫大，肝胆道系酵素の上昇，頸静脈怒張，右心不全が高度なときは肺うっ血所見が乏しい

低心拍出量による自覚症状と身体所見	
自覚症状	意識障害，不穏，記銘力低下
身体所見	冷汗，四肢冷感，チアノーゼ，低血圧，乏尿，身の置き場がない様相

※２つ以上の大基準，もしくは１つの大基準と２つ以上の小基準を満たす場合，心不全と診断する

What is 心不全？

定義	LVEF	説明
**LVEFの低下した心不全：HFrEF**<br>（heart failure with reduced ejection fraction）	40%未満	収縮不全が主体．現在の多くの研究では標準的心不全治療下での LVEF低下例がHFrEFとして組み入れられている．
**LVEFの保たれた心不全：HFpEF**<br>（heart failure with preserved ejection fraction）	50%以上	拡張不全が主体．診断は心不全と同様の症状をきたす他疾患の除外が必要である．有効な治療が十分には確立されていない．
**LVEFが軽度低下した心不全：HFmrEF**<br>（heart failure with mid- range ejection fraction）	40%以上<br>50% 未満	境界型心不全．臨床的特徴や予後は研究が不十分であり，治療選択は個々の病態に応じて判断する．
**LVEFが改善した心不全：HFrecEF**<br>（heart failure with preserved ejection fraction, improved; HFpEF improvedまたはheart failure with recovered EF; HFrecEF）	40% 以上	LVEFが 40% 未満であった患者が治療経過で改善した患者群．HFrEFとは予後が異なる可能性が示唆されているが，さらなる研究が必要である．

What is 心不全？

急性心不全の患者を診たら…

MR CHAMPH	
**M**yocarditis	心筋炎
**R**ight-sided heart failure	右心不全
Acute **C**oronary syndrome	急性冠症候群
**H**ypertensive emergency	高血圧緊急症
**A**rrhythmia	不整脈
Acute **M**echanical cause	機械的合併症<br>（自由壁破裂，心室中隔穿孔，乳頭筋断裂など）
Acute **P**ulmonary thromboembolism	急性肺血栓塞栓症
**H**igh output heart failure	高拍出性心不全<br>（敗血症，甲状腺中毒症，貧血，<br>短絡性心疾患，脚気心，パジェット病）

What is 心不全？

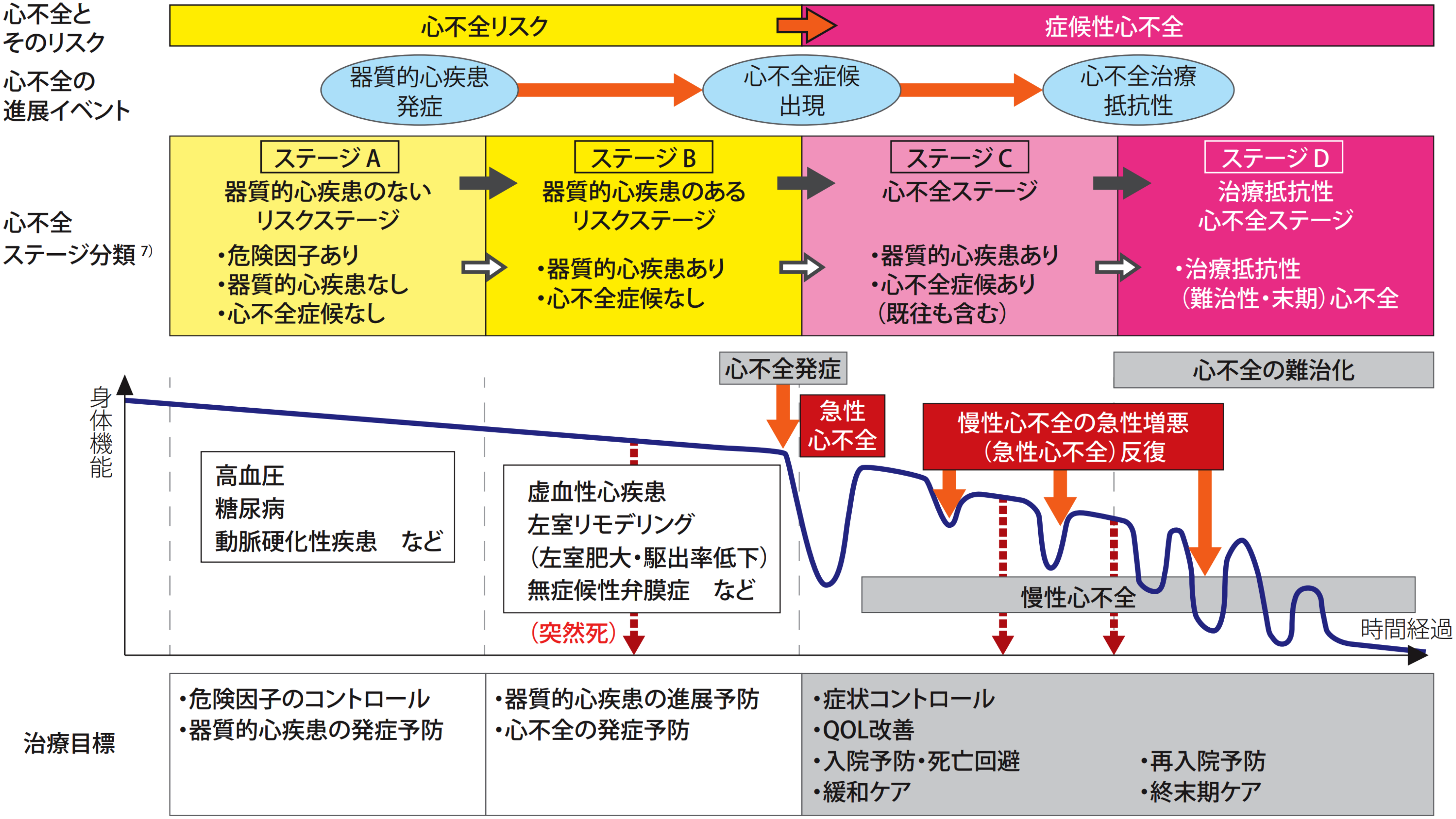

症状が顕在化する前からの早期治療介入の有用性が確認されている現在では，急性•慢性の分類の重要性は薄れている．

What is 心不全？

心不全の増悪因子		
F	Forgot meds	怠薬
A	Arrhythmia<br>Afterload<br>Anemia	不整脈<br>血圧高値<br>貧血
I	Infection<br>Ischemia<br>Infarction	**感染症**<br>狭心症<br>心筋梗塞など
L	Lifestyle	塩分過剰,<br>アルコール摂取,<br>ストレス
U	Upregulators	甲状腺疾患, 妊娠,<br>脚気心
R	Rheumatic valve<br>Regurgitation	リウマチ性弁<br>弁膜症
E	Embolism	肺塞栓症

低灌流所見の有無 ＼ うっ血所見の有無	なし	あり
なし	dry-warm<br>A	wet-warm<br>B
あり	dry-cold<br>L	wet-cold<br>C

低灌流：小さい脈圧, 四肢冷感, 傾眠傾向,
低Na血症, 腎機能悪化

うっ血所見：起座呼吸, 頸静脈圧の上昇, 浮腫
腹水, 肝頸静脈逆流

What is 心不全？

Clinical Scenario（CS）					
	CS1	CS2	CS3	CS4	CS5
分類	sBP **>140**mmHg	sBP **100~140**mmHg	sBP**<100**mmHg	**急性冠症候群**	**右心不全**
病態生理	・充満圧上昇による急性発症<br>・血管性要因が関与<br>・全身性浮腫は軽度<br>・体液量は正常もしくは低下	・慢性の充満圧/静脈圧/肺動脈圧上昇による緩徐な発症<br>・臓器障害/腎/肝障害/貧血/低アルブミン血症<br>・肺水腫は軽度	・発症様式は急性あるいは緩徐<br>・全身性浮腫/肺水腫は軽度	・急性心不全の症状,徴候<br>・トロポニン単独の上昇はCS4に分類しない	・発症様式は急性あるいは緩徐<br>・肺水腫はない<br>・全身性静脈の鬱血徴候
治療	**血管拡張薬**<br>**NPPV**<br>(体液過剰あれば)利尿薬	**利尿薬**<br>血管拡張薬<br>NPPV	**容量負荷**<br>**強心薬**<br>血管収縮薬	**再灌流療法**	**利尿薬**<br>**強心薬**<br>血管収縮薬<br>※容量負荷は行わない

What is 心不全？

本症例では…

もともと労作時呼吸困難があり心不全を有した可能性？

↓

蜂窩織炎(感染)や発作性心房細動(不整脈)を契機とした心不全増悪

↓

Wet-WarmのCS1心不全が疑わしい

EBMグランプリ開始

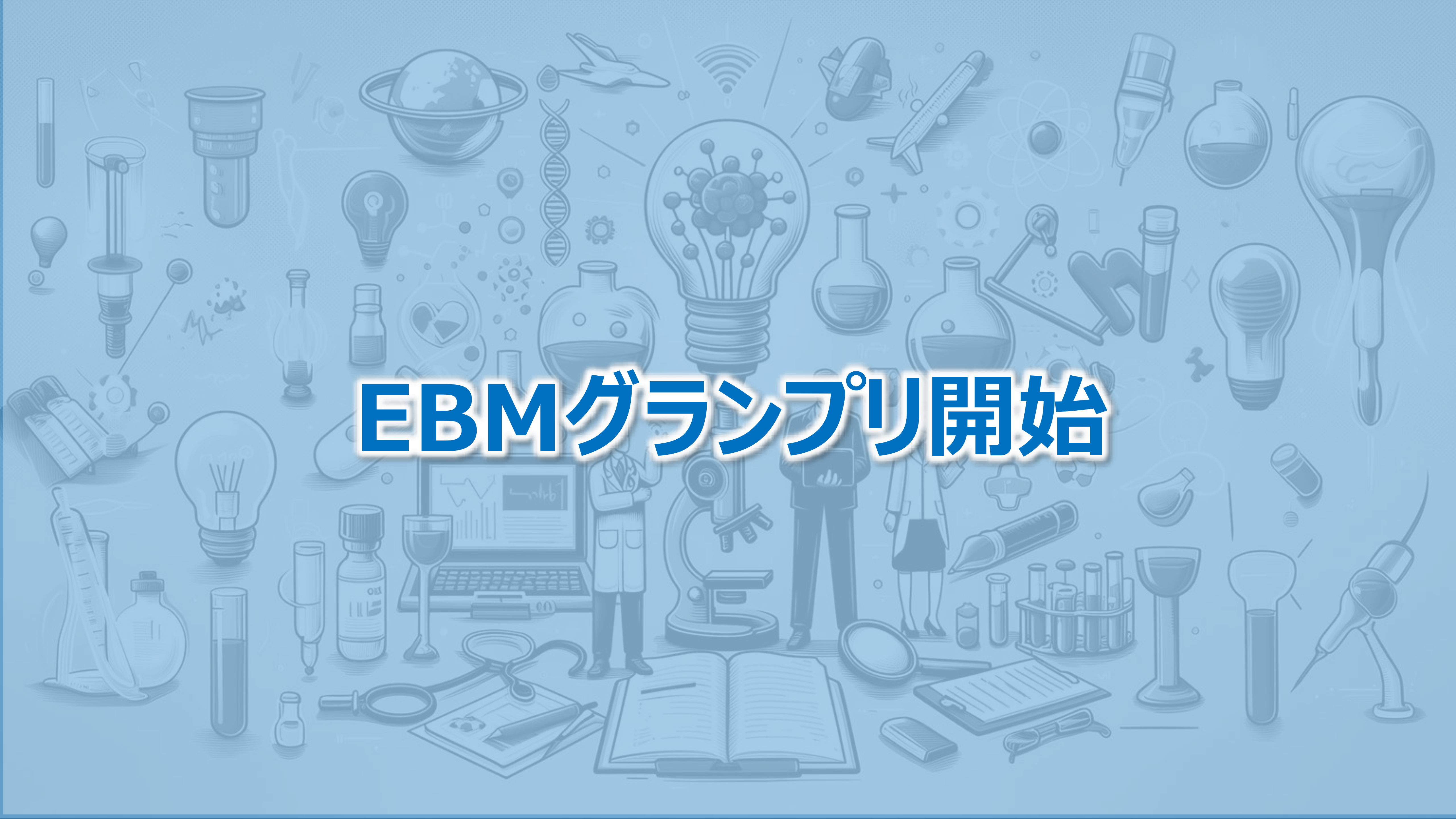

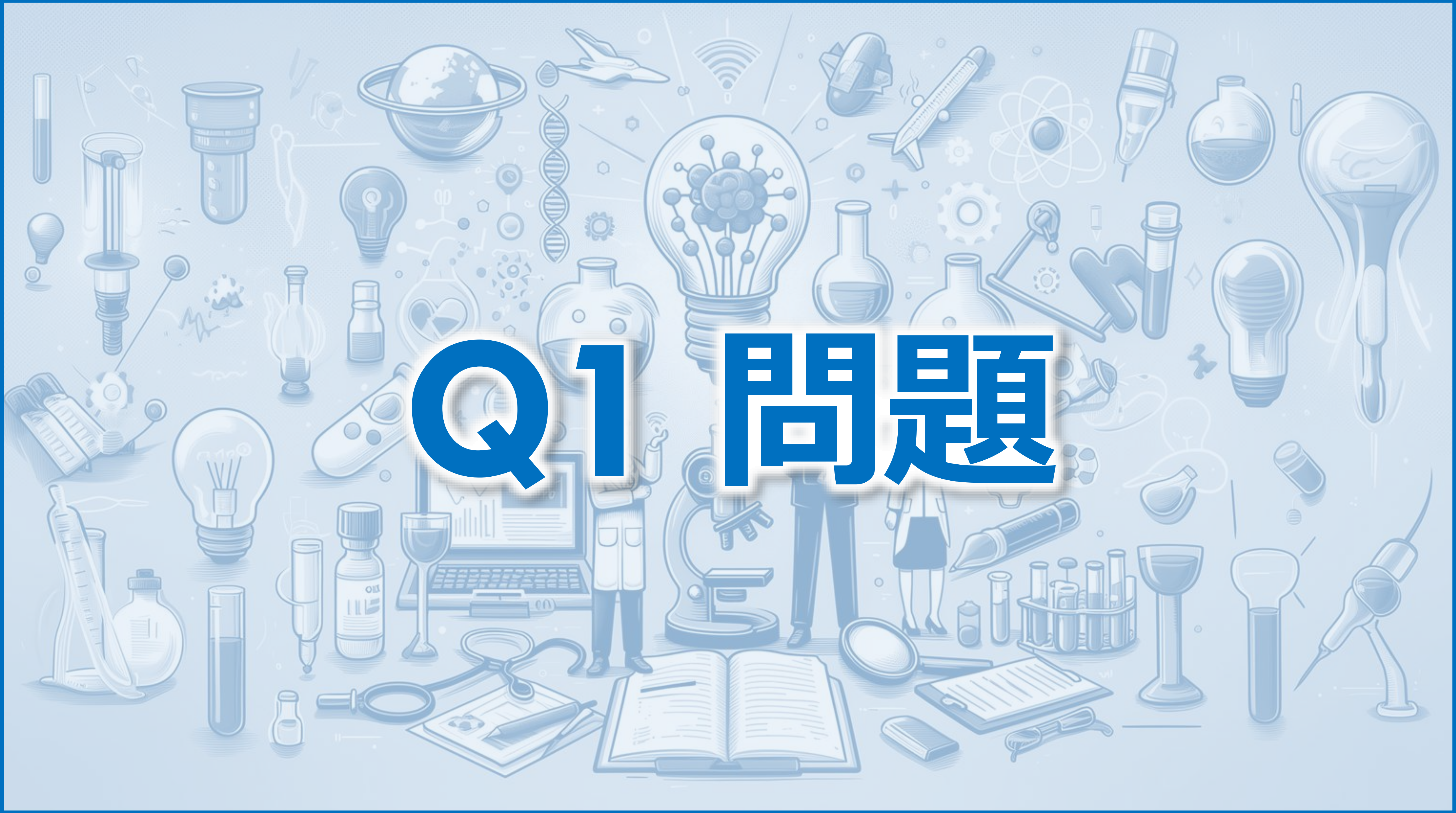
Q1 問題

Case 2 Q1 前半10 分

この患者は菌血症の可能性が高いから血液培養を採取しよう！と指導医より提案された。血液培養のエビデンスについて、下記の選択肢の中から正しいものを全て選びなさい

a. 悪寒戦慄は菌血症のリスクとなる

b. 血液培養は2セット以上採取する

c. 静脈カテーテルから血液培養を採取する

d. 滅菌手袋を着用して血液培養を採取する

e. 蜂窩織炎のある部位の大腿の血管から採取する

Case 2 Q1 後半5分

この患者は菌血症の可能性が高いから血液培養を採取しよう！
と指導医より提案された。血液培養のエビデンスについて、
下記の選択肢の中から正しいものを全て選びなさい

a. 悪寒戦慄は菌血症のリスクとなる

b. 血液培養は2セット以上採取する

c. 静脈カテーテルから血液培養を採取する

d. 滅菌手袋を着用して血液培養を採取する

e. 蜂窩織炎のある部位の大腿の血管から採取する

Q1 解説

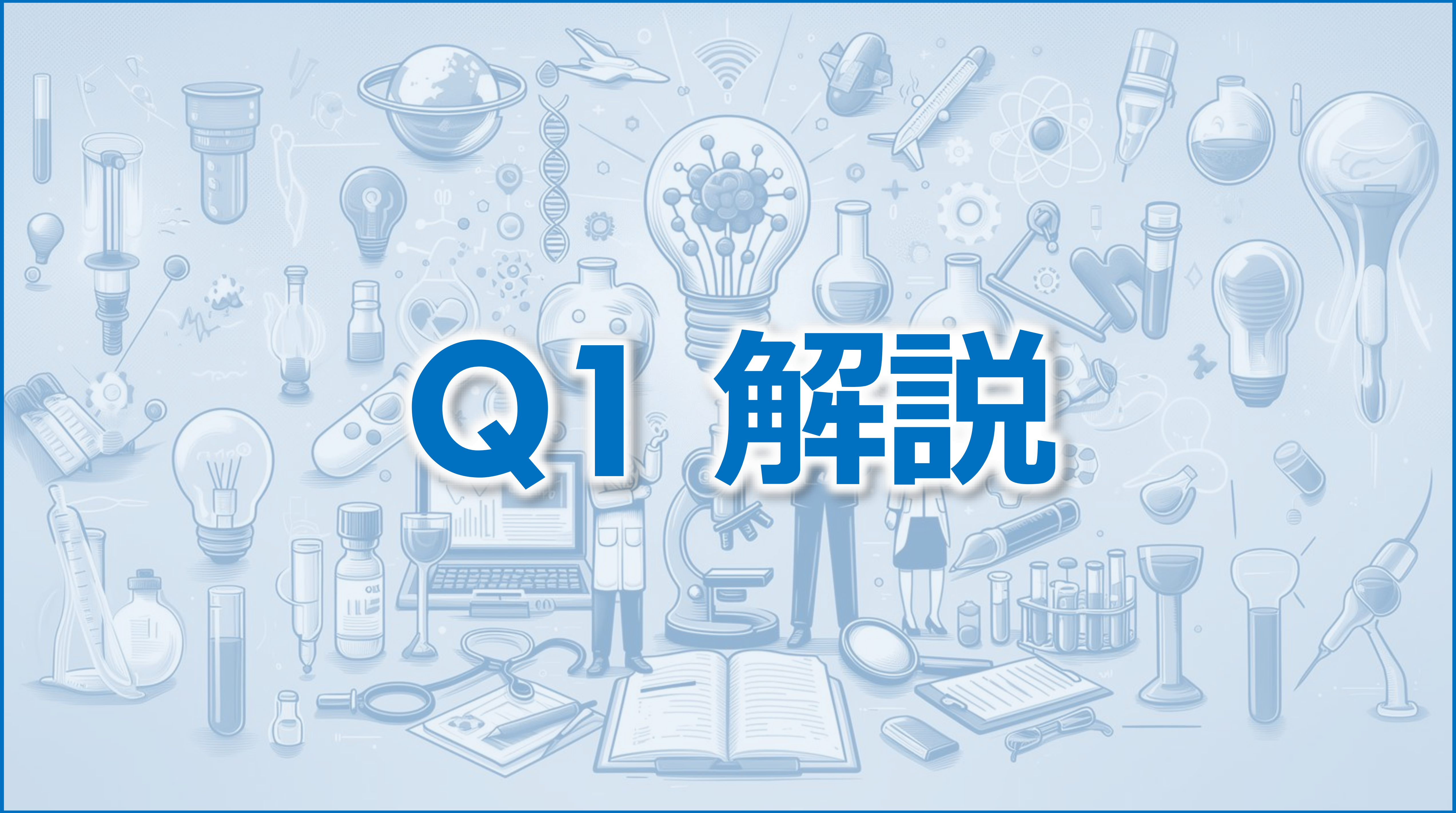

Case 2 Q1

P (患者)： 急性発熱疾患を有し、病院の救急部に来院した15歳以上の成人
I (介入)： 軽度、中等度、激しい悪寒を伴う患者
C (比較)： 悪寒が全くない患者
O (アウトカム)： 悪寒の重症度が細菌血症リスクに与える影響(リスク比,診断特性)

比較基準	感度 (%)	特異度 (%)	陽性的中率 (PPV: %)	陰性的中率 (NPV: %)	陽性尤度比 (PLR)	陰性尤度比 (NLR)
悪寒あり vs 悪寒なし	**87.5**	51.6	13	98.8	1.81	0.24
激しい悪寒 vs 他全て	45	**90.3**	27.7	95.2	4.65	0.61

1. **悪寒がない場合:** 細菌血症の可能性はほとんどない（見逃しの心配が少ない）。
2. **激しい悪寒がある場合:** 細菌血症である可能性が高いので、血液培養や抗菌薬治療が必要。
3. **悪寒の有無や重症度を聞き取ることは、簡単で信頼性の高い診断法の一つ。**

Tokuda Y et al. Am J Med 2005; 118(12): 1417

Case 2 Q1

蜂窩織炎、丹毒、皮膚膿瘍が疑われる患者、特に高齢者（65歳以上）において血液培養をルーチンで2セット採取することの有用性を明らかにした観察研究

Characteristic	Unadjusted OR (95% CI)	Adjusted OR (95% CI)	*P* Value
Age	1.03 (1.01–1.06)	1.04 (1.01–1.07)	.004[a]
Recent antibiotic use within 48 h	.07 (.01–.55)	.08 (.01–.60)	.014[a]
Shaking chills	1.96 (.97–3.96)	2.63 (1.12–6.15)	.025[a]
WBC count ≥13 000/µL	2.84 (1.45–5.57)	2.81 (1.34–5.92)	.006[a]
Severe infection[b]	3.78 (.74–19.4)	8.54 (1.26–58.0)	.028[a]
Comorbidities[c]	1.04 (.54–1.99)	1.07 (.53–2.14)	.86
Bacterial coinfection other than cellulitis	3.38 (1.42–8.03)	1.54 (.59–4.02)	.38

Taniguchi T et al. Open Forum Infect Dis 2022; 9(7): ofac317

- 血液培養陽性のリスク因子として**高齢（年齢増加）,悪寒戦慄, 白血球増加, 重症感染**が独立した因子である.
- **48時間以内の抗菌薬使用**は陽性率を大幅に低下させ, 抗菌薬投与前に血液培養を採取することが推奨される.

Detection of bacteremia: Blood cultures and other diagnostic tests

Site selection — When possible, blood cultures should be obtained via venipuncture, given the lower likelihood of contamination compared with blood cultures collected through vascular catheters (even if obtained at the time of catheter insertion). In one meta-analysis including nine studies and more than 13,000 blood cultures, the likelihood of contamination was higher for blood cultures collected through an intravascular catheter than blood cultures collected by venipuncture (odds ratio 2.69, 95% CI 2.03-3.57) [10].

Preferred venipuncture sites include the antecubital veins or other upper extremity blood vessels; these sites are less likely to be associated with blood culture contamination than femoral vessels or sites affected by dermatologic disease [4]. Venous and arterial blood cultures have comparable yield [11,12].

・静脈カテーテルからの培養採取は非推奨
・大腿の血管や皮膚疾患に罹患している部位より，
上肢の血管が好まれる

Detection of bacteremia: Blood cultures and other diagnostic tests

● If further vein palpation is necessary after skin preparation, a sterile glove should be worn [17,18].

Number of blood culture sets — A blood culture set, as noted above, usually consists of one aerobic bottle and one anaerobic bottle. At least two, preferably three, blood culture sets should be obtained [1,3-5]. In studies evaluating the yield of four or more blood cultures, the cumulative yield of true pathogens increased with the number of cultures collected (one culture; 73 to 80 percent, two cultures: 80 to 89 percent, three cultures: 95 to 98 percent, and four cultures: 99 to 100 percent) [36,37].

A total of two blood culture sets is usually adequate when continuous bacteremia is suspected, and the pretest probability of bacteremia is high (as in patients with suspected IE who have not received prior antimicrobial therapy). (See "Clinical manifestations and evaluation of adults with suspected left-sided native valve endocarditis".)

- 滅菌手袋の着用を推奨
- 2セット以上の血液培養の採取を推奨

Case 2 Q1

この患者は菌血症の可能性が高いから血液培養を採取しよう！
と指導医より提案された。血液培養のエビデンスについて、
下記の選択肢の中から正しいものを全て選びなさい

a. 悪寒戦慄は菌血症のリスクとなる

b. 血液培養は2セット以上採取する

c. 静脈カテーテルから血液培養を採取する

d. 滅菌手袋を着用して血液培養を採取する

e. 蜂窩織炎のある部位の大腿の血管から採取する

Q2 問題

Case 2 Q2 前半10 分

この患者は急性呼吸不全をきたしており、指導医と酸素デバイスを検討することになった。
この患者に対する酸素デバイスのエビデンスについて調べています。下記の選択肢で、正しい選択肢を全て答えなさい

a. HFNCはNIVと比較し、死亡率を低下せる
b. NIVは通常の酸素療法と比較し、挿管を回避させる
c. NIVは通常の酸素療法と比較し、死亡率を低下させる
d. HFNCは通常の酸素療法と比較し、挿管を回避させる
e. HFNCは通常の酸素療法と比較し、死亡率を低下させる

HFNC：高流量鼻腔カニュラ酸素療法(ネーザルハイフロー)、
NIV：非侵襲的換気(NPPVやCPAP)
通常の酸素療法：ネーザルカニュラ、フェイスマスク

Case 2 Q2

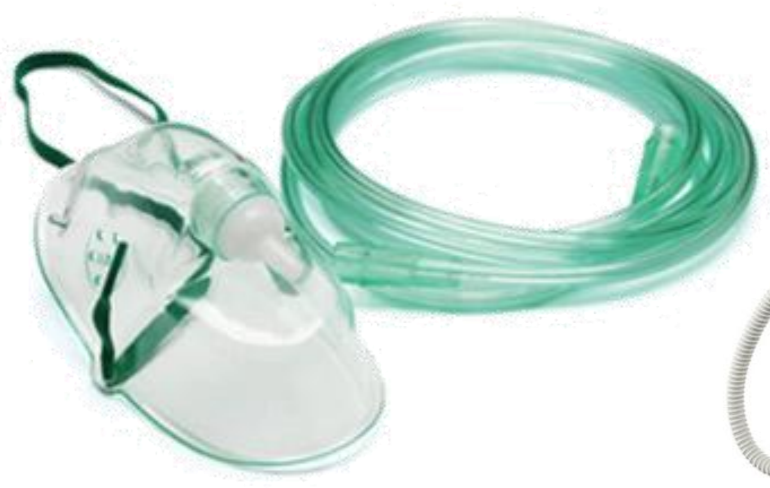

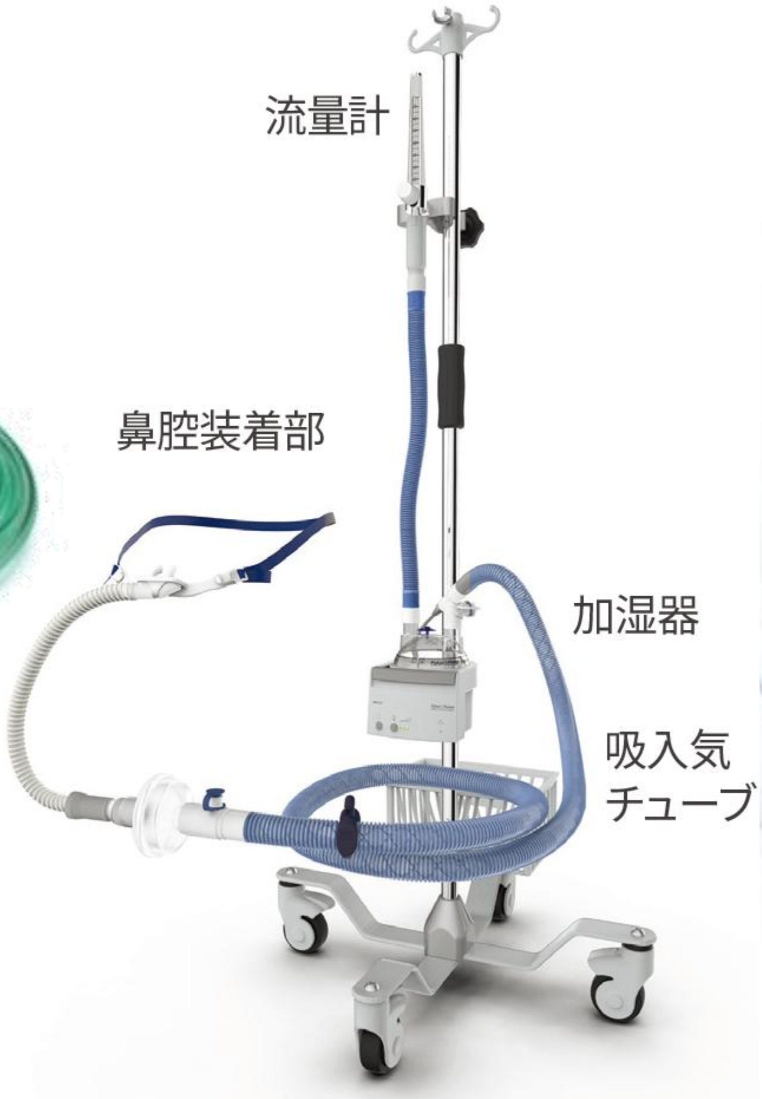

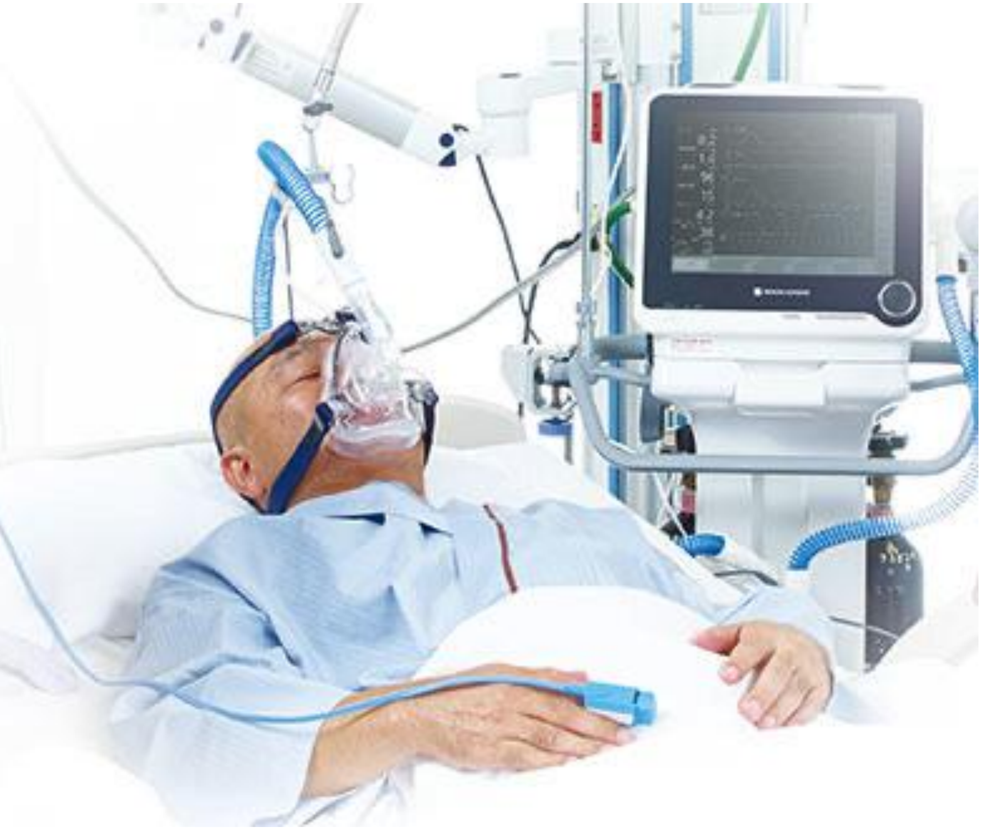

Face Mask **High Flow Nasal Cannula** **Non-Invasive Ventilation(NIV)**

Case 2 Q2 後半5分

この患者は急性呼吸不全をきたしており、指導医と酸素デバイスを検討することになった。
この患者に対する酸素デバイスのエビデンスについて調べています。下記の選択肢で、正しい選択肢を全て答えなさい

a. HFNCはNIVと比較し、死亡率を低下せる
b. NIVは通常の酸素療法と比較し、挿管を回避させる
c. NIVは通常の酸素療法と比較し、死亡率を低下させる
d. HFNCは通常の酸素療法と比較し、挿管を回避させる
e. HFNCは通常の酸素療法と比較し、死亡率を低下させる

HFNC：高流量鼻腔カニュラ酸素療法(ネーザルハイフロー)、
NIV：非侵襲的換気(NPPVやCPAP)
通常の酸素療法：ネーザルカニュラ、フェイスマスク

Q2 解説

Case 2 Q2

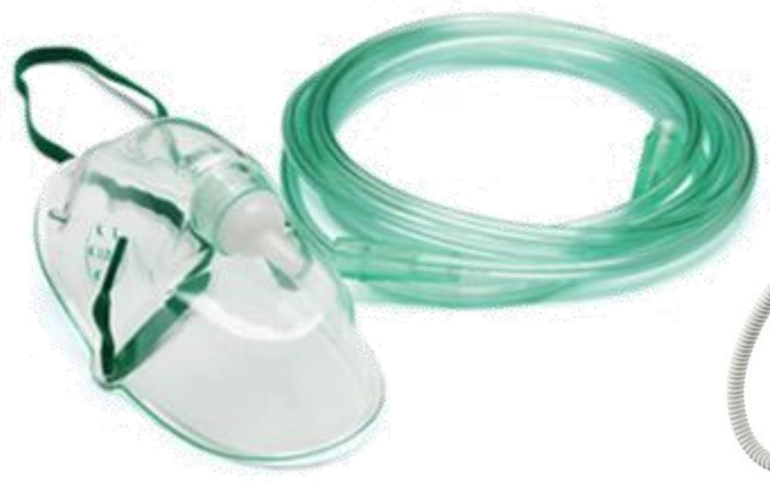

Face Mask

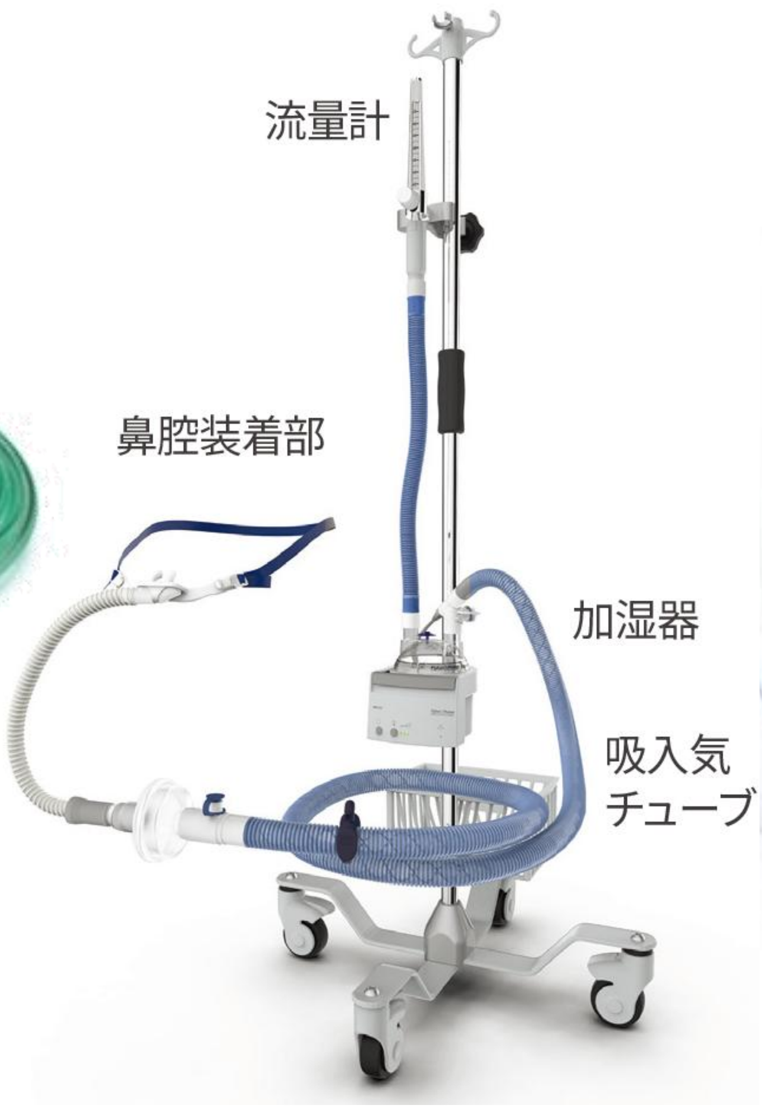

High Flow Nasal Cannula

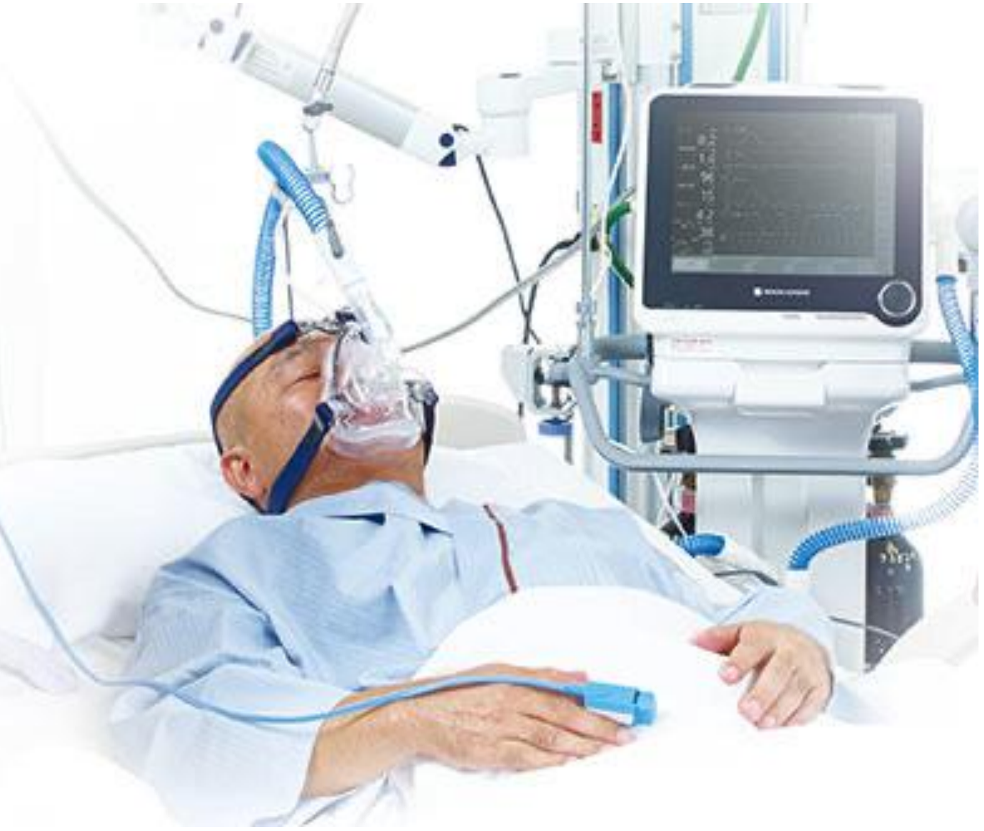

Non-Invasive Ventilation(NIV)

Case 2 Q2

P (患者)： 急性心原性肺水腫（ACPE）の成人患者
I (介入)： 非侵襲的陽圧換気（NPPV、CPAP）
C (比較)： 標準的医療（SMC）
O (アウトカム)： 主: 入院死亡率, 副: 気管挿管率、急性心筋梗塞の発生率など

Outcomes	Anticipated absolute effects* (95% CI)		Relative effect (95% CI)	№ of participants (studies)	Certainty of the evidence (GRADE)	Comments
	Risk with SMC	Risk with NPPV				
HOSPITAL MORTALITY follow-up: median 13 days; range 1 day - 41 days	Study population		RR 0.65 (0.51 to 0.82)	2484 (21 RCTs)	⊕⊕⊝⊝ LOW[a,b]	-
	176 per 1000	114 per 1000 (90 to 144)				
ETI RATE follow-up: median 1 day; range 0.1 day - 30 days	Study population		RR 0.49 (0.38 to 0.62)	2449 (20 RCTs)	⊕⊕⊕⊝ MODERATE[c]	-
	154 per 1000	75 per 1000 (58 to 95)				
ACUTE MI INCIDENCE follow-up: median 3 days; range 1 day - 41 days	Study population		RR 1.03 (0.91 to 1.16)	1313 (5 RCTs)	⊕⊕⊕⊝ MODERATE[d]	-
	421 per 1000	433 per 1000 (383 to 488)				

Berbenetz N et al. Cochrane Database Syst Rev 2019; 4(4): CD005351

Case 2 Q2

- **P (患者):** 急性心不全（Acute Heart Failure; AHF）患者
- **I (介入):** 高流量鼻カニュラ（High-Flow Nasal Cannula; HFNC）療法
- **C (比較対象):** 従来の酸素療法または非侵襲的人工呼吸（Non-Invasive Ventilation; NIV）
- **O (アウトカム):** 挿管率、呼吸数、心拍数

a

Study or Subgroup	HFNC Events	HFNC Total	COT Events	COT Total	Weight	Odds Ratio M-H, Fixed, 95% CI
Ko DR 2020	1	34	1	33	3.0%	0.97 [0.06, 16.17]
Li QH 2021	2	39	9	39	26.4%	0.18 [0.04, 0.90]
Li YF 2022	3	38	11	38	31.3%	0.21 [0.05, 0.83]
Makdee O 2017	1	63	0	65	1.5%	3.14 [0.13, 78.63]
Yang FJ 2021	4	64	13	64	37.7%	0.26 [0.08, 0.85]
**Total (95% CI)**		**238**		**239**	**100.0%**	**0.29 [0.14, 0.58]**
Total events	11		34			

Heterogeneity: Chi² = 3.39, df = 4 (P = 0.50); I² = 0%
Test for overall effect: Z = 3.49 (P = 0.0005)

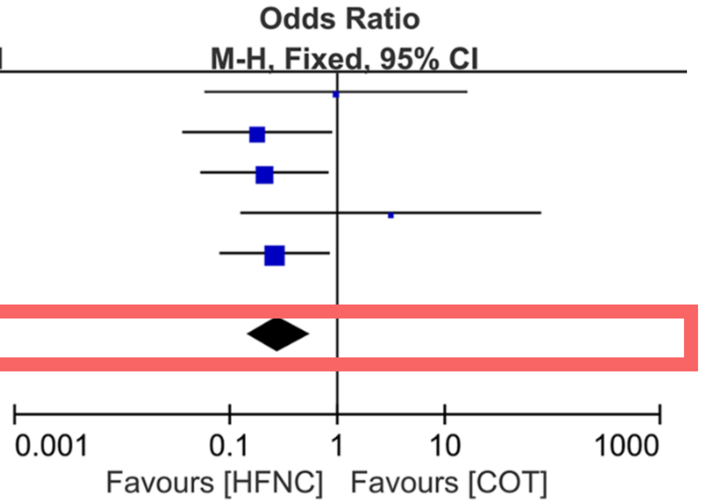

b

Study or Subgroup	HFNC Events	HFNC Total	NIV Events	NIV Total	Weight	Odds Ratio M-H, Fixed, 95% CI
Li F 2020	1	21	3	20	22.6%	0.28 [0.03, 2.98]
Liu SX 2022	4	63	4	60	29.6%	0.95 [0.23, 3.98]
Osman A 2021	11	94	7	94	47.7%	1.65 [0.61, 4.45]
**Total (95% CI)**		**178**		**174**	**100.0%**	**1.13 [0.54, 2.39]**
Total events	16		14			

Heterogeneity: Chi² = 1.94, df = 2 (P = 0.38); I² = 0%
Test for overall effect: Z = 0.32 (P = 0.75)

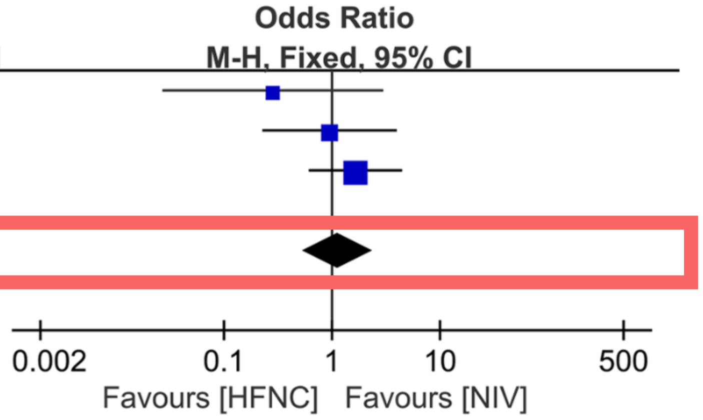

Yan L et al. BMC Pulm Med 2023; 23(1): 476

Case 2 Q2

この患者は急性呼吸不全をきたしており、指導医と酸素デバイスを検討することになった。
この患者に対する酸素デバイスのエビデンスについて調べています。下記の選択肢で、正しい選択肢を全て答えなさい

a. HFNCはNIVと比較し、死亡率を低下せる
b. NIVは通常の酸素療法と比較し、挿管を回避させる
c. NIVは通常の酸素療法と比較し、死亡率を低下させる
d. HFNCは通常の酸素療法と比較し、挿管を回避させる
e. HFNCは通常の酸素療法と比較し、死亡率を低下させる

HFNC：高流量鼻腔カニュラ酸素療法(ネーザルハイフロー)、
NIV：非侵襲的換気(NPPVやCPAP)
通常の酸素療法：ネーザルカニュラ、フェイスマスク

Noninvasive ventilation in adults with acute respiratory failure: Benefits and contraindications

Acute cardiogenic pulmonary edema (ACPE) — The mechanism by which NIV improves ACPE is thought to be due to preload reduction, the prevention of alveolar collapse at end expiration, and decreased left ventricular afterload. For patients with ACPE, we recommend a trial of NIV, typically with CPAP. Meta-analyses of small randomized trials in patients with ACPE, report that NIV decreases the need for intubation, improves clinical and laboratory indices of respiratory failure (eg, heart rate, dyspnea, hypercapnia, acidosis), and improves mortality [1,7,15-22]. As an example, a 2013 meta-analysis of 32 studies (2916 patients) that included both modalities of NIV (CPAP and bilevel), reported that NIV significantly reduced hospital mortality in patients with ACPE compared with standard medical care (RR 0.66, 95% CI 0.48-0.89) [21]. NIV also reduced rates of endotracheal intubation (RR 0.52, 95% CI 0.36 to 0.75) but did not have any impact on hospital length of stay. We prefer to use CPAP as initial therapy in patients with ACPE, since evidence using this mode of NIV is more robust in this population; however some patients may be initiated on bilevel NIV (eg, patients with acute hypercapnia due to ACPE).

The impact of NIV on mortality in patients with ACPE is variable and may relate to differences in the type of NIV used and the presence or absence of acute hypercapnia. As examples:

- A meta-analysis of 13 trials that included 1369 patients found that patients with ACPE who received CPAP plus standard care had a lower hospital mortality than those who received standard care alone (10.3 versus 15.8 percent; RR 0.64, 95% CI 0.44-0.92) [19]. However, in the same analysis, treatment with bilevel NIV (nine trials, 1091 patients) was only associated with a trend towards improved mortality that did not reach statistical significance (9.6 versus 11.9 percent; RR 0.82, 95% CI 0.58-1.15).

- Several studies found that patients with ACPE who present with acute hypercapnic respiratory failure derive the greatest mortality benefit, although this benefit has not been directly attributed to CPAP or bilevel NIV [22,23].

- A randomized trial that compared CPAP with PSV delivered noninvasively found no difference in either mortality or the frequency of intubations, although respiratory distress appeared to resolve sooner among patients who received PSV, probably because it is a more comfortable mode of ventilation [24].

The practical aspects of applying CPAP and the medical treatment of acute decompensated heart failure are discussed separately. (See "Noninvasive ventilation in adults with acute respiratory failure: Practical aspects of initiation", section on 'Continuous positive airway pressure (CPAP)' and "Treatment of acute decompensated heart failure: General considerations".)

UpToDateでもNIVの院内死亡率の低下や挿管のリスク低下について言及されている。

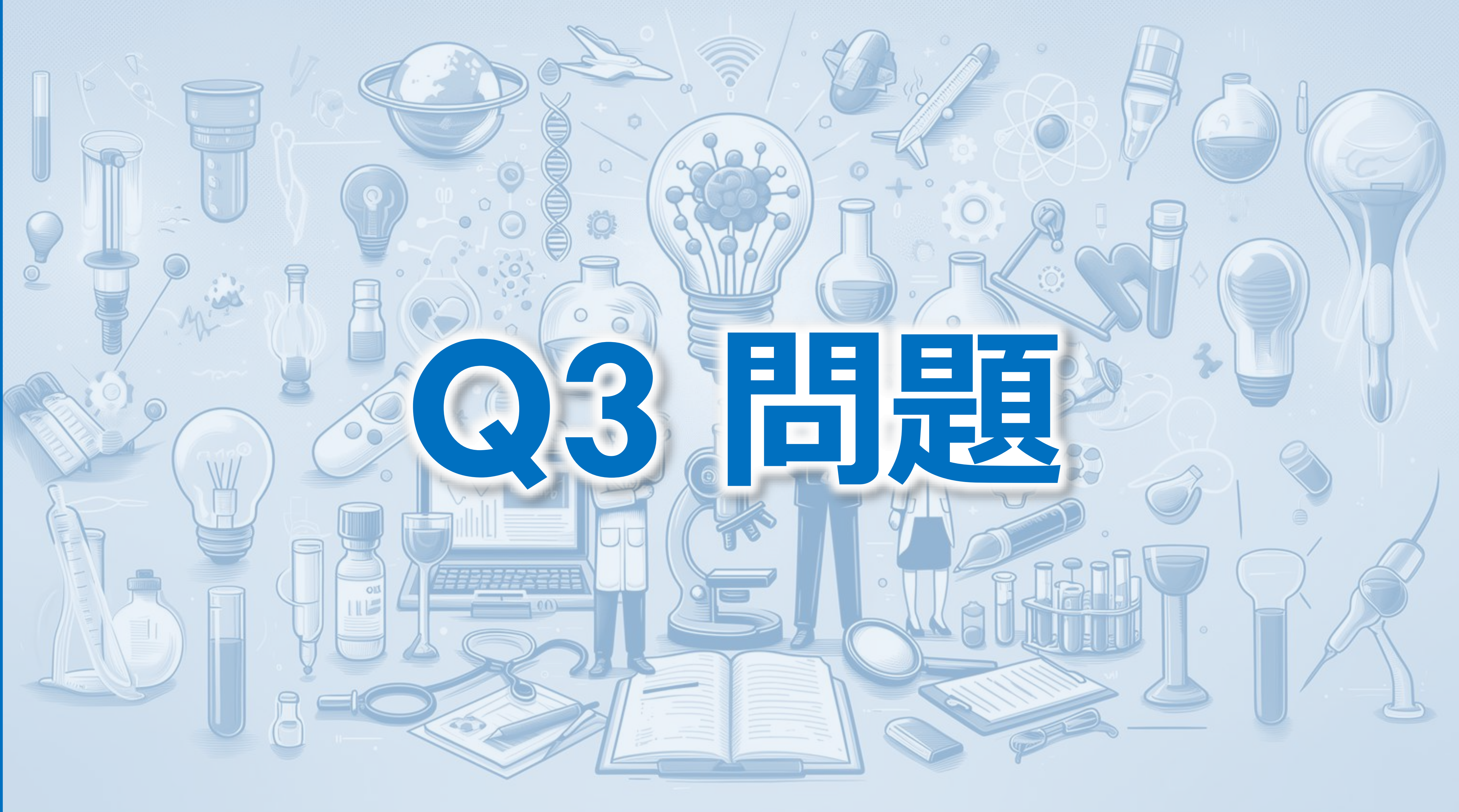

Q3 問題

Case 2 Q3 前半10 分

この患者に対してトルバプタンの使用を検討することになった。トルバプタンのランダム化比較試験のエビデンスについて調べています。下記の選択肢で、正しい選択肢を全て答えなさい

a. トルバプタンはQOLを改善した報告がある
b. トルバプタンは死亡率を低下させた報告がある
c. トルバプタンは入院期間を短縮させた報告がある
d. トルバプタンは他の利尿薬を減量できた報告がある
e. トルバプタンは血清ナトリウム濃度を上昇させた報告がある

Case 2 Q3 後半5分

この患者に対してトルバプタンの使用を検討することになった。トルバプタンのランダム化比較試験のエビデンスについて調べています。下記の選択肢で、正しい選択肢を全て答えなさい

a. トルバプタンはQOLを改善した報告がある
b. トルバプタンは死亡率を低下させた報告がある
c. トルバプタンは入院期間を短縮させた報告がある
d. トルバプタンは他の利尿薬を減量できた報告がある
e. トルバプタンは血清ナトリウム濃度を上昇させた報告がある

Q3 解説

Case 2 Q3

- **P (患者):** 入院を要する心不全悪化患者4133例
- **I (介入):** トルバプタン30 mg/日を標準治療に追加。
- **C (比較対象):** プラセボを標準治療に追加
- **O (アウトカム):** 全死亡率、心血管死, 心不全入院。体重、症状(呼吸困難、浮腫)、血清ナトリウム値

	No. (%) of Patients		Hazard Ratio (95% Confidence Interval)	*P* Value	
	Tolvaptan (n = 2072)	Placebo (n = 2061)		Superiority	Noninferiority
Primary end points					
All-cause mortality	537 (25.9)	543 (26.3)	0.98 (0.87-1.11)	.68*	<.001

- **心血管死または心不全入院: 両群で有意差なし。**
- **初日の呼吸困難スコア改善、体重減少、血清ナトリウム値上昇に有意な効果を示した。**

JAMA. 2007;297(12):1319-1331.

Case 2 Q3

- **P (患者):** 入院を要する難治性心不全患者
- **I (介入):** トルバプタンを従来の利尿薬に追加投与（3.75–7.5 mg/日）
- **C (比較対象):** 従来の利尿薬治療のみ
- **O (アウトカム):** QOL（MLHFQ score, 腎機能(血清クレアチニン、血清ナトリウム値)、心不全再入院率

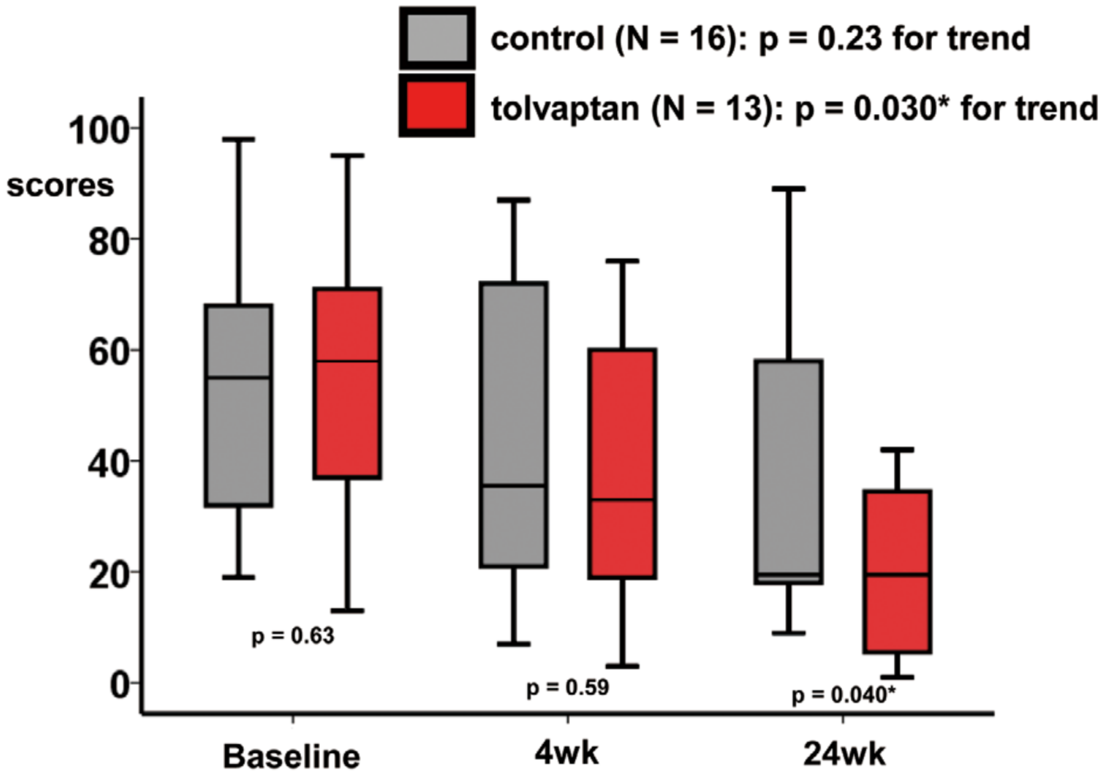

Circ Rep. 2019 Sep 26;1(10):431-437.

Figure 2. Primary endpoint: trends of quality of life quantified using the Minnesota Living with Heart Failure Questionnaire. *P<0.05. Intergroup comparisons between the tolvaptan group and the control group were performed with the Mann-Whitney U-test. Intra-group trends from baseline to 24 weeks were assessed with the Friedman test.

- **QOL（MLHFQスコア）：**
  - トルバプタン群でベースライン58から10へ有意に改善（P=0.030）コントロール群では改善なし。
  - 6か月後のスコア比較ではトルバプタン群が有意に優れていた（P=0.040）。

Treatment of acute decompensated heart failure: Specific therapies

Vasopressin receptor antagonists — Vasopressin receptor antagonists have been investigated as an adjunct to diuretics and other standard therapies in patients with ADHF as a means of countering arterial vasoconstriction, hyponatremia, and water retention. Tolvaptan is the most studied agent in this setting. However, such treatment is controversial since the long-term safety and benefit of this approach are unknown. (See "Hyponatremia in patients with heart failure", section on 'Efficacy'.)

For patients with HF with volume overload with persistent severe hyponatremia (ie, serum sodium ≤120 mEq/L) despite water restriction and maintenance of guideline-directed medical therapy, short-term use of a vasopressin receptor antagonist (either a V_2 receptor selective or nonselective vasopressin antagonist) is an option to improve serum sodium concentration [5]. Cautions include hepatotoxicity (with the US Food and Drug Administration determining that tolvaptan should **not** be used in any patient for longer than 30 days and should **not** be used at all in patients with liver disease due to risk of liver failure or death) and overly rapid correction of hyponatremia, which can lead to irreversible neurologic injury. These issues are further discussed separately. (See "Hyponatremia in patients with heart failure", section on 'Vasopressin receptor antagonists'.)

UpToDateでもTolvaptanは長期安全性とbenefitが確認されていないとの記載あり

suggest consideration of tolvaptan for HF patients with hyponatremia in an ungraded recommendation [illegible].

Case 2 Q3

この患者に対してトルバプタンの使用を検討することになった。トルバプタンのランダム化比較試験のエビデンスについて調べています。下記の選択肢で、正しい選択肢を全て答えなさい

a. トルバプタンはQOLを改善した報告がある
b. トルバプタンは死亡率を低下させた報告がある
c. トルバプタンは入院期間を短縮させた報告がある
d. トルバプタンは他の利尿薬を減量できた報告がある
e. トルバプタンは血清ナトリウム濃度を上昇させた報告がある

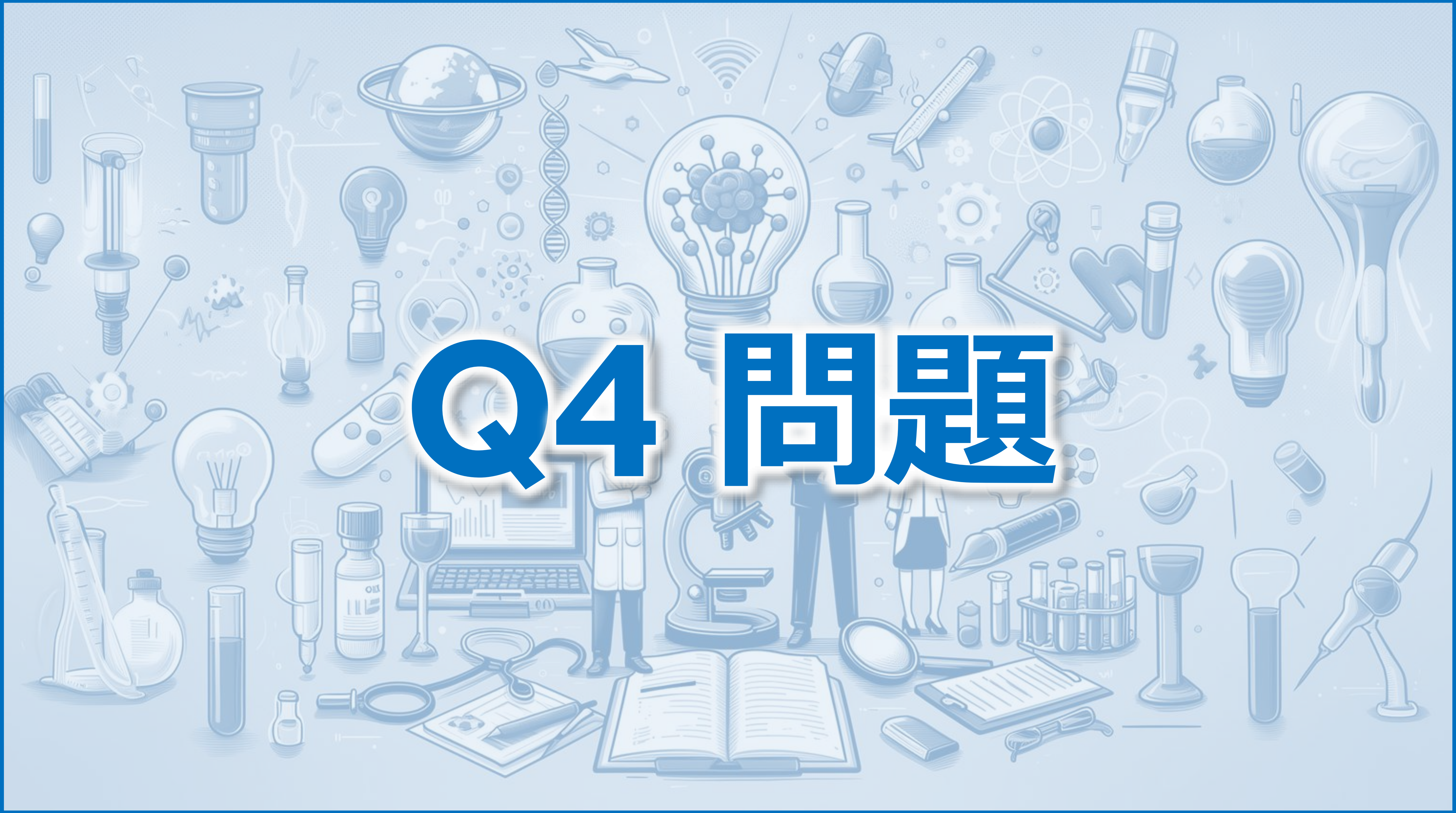

Q4 問題

Case 2 Q4 前半10 分

感染のコントロールがついたため再度、心臓超音波検査を施行したところEF23%と低下を認めた。心不全の慢性期管理を開始する事とした。
この患者において、下記薬剤を組み合わせて内服した際に最も平均生存期間の延長に寄与するものはどれか？

a. ACEi/ARB + ARNI + SGLT2i + βblocker
b. ACEi/ARB +フロセミド + SGLT2i + βblocker
c. ARNI + SGLT2i + MRA + βblocker
d. フロセミド + SGLT2i + MRA + βblocker
e. ACEi/ARB + ARNI +フロセミド + SGLT2i

ACEi ：ACE阻害薬、ARB：アンジオテンシン受容体拮抗薬、
ARNI:アンジオテンシン受容体ネプリリシン阻害薬、
SGLT2i：SGLT2阻害薬、MRA：ミネラルコルチコイド受容体拮抗薬、βblocker ：β遮断薬

Case 2 Q4 後半5 分

感染のコントロールがついたため再度、心臓超音波検査を施行したところEF23%と低下を認めた。心不全の慢性期管理を開始する事とした。
この患者において、下記薬剤を組み合わせて内服した際に最も平均生存期間の延長に寄与するものはどれか？

a. ACEi/ARB + ARNI + SGLT2i + βblocker
b. ACEi/ARB +フロセミド + SGLT2i + βblocker
c. ARNI + SGLT2i + MRA + βblocker
d. フロセミド + SGLT2i + MRA + βblocker
e. ACEi/ARB + ARNI +フロセミド + SGLT2i

ACEi ：ACE阻害薬、ARB：アンジオテンシン受容体拮抗薬、
ARNI:アンジオテンシン受容体ネプリリシン阻害薬、
SGLT2i：SGLT2阻害薬、MRA：ミネラルコルチコイド受容体拮抗薬、βblocker ：β遮断薬

Q4 解説

Case 2 Q4

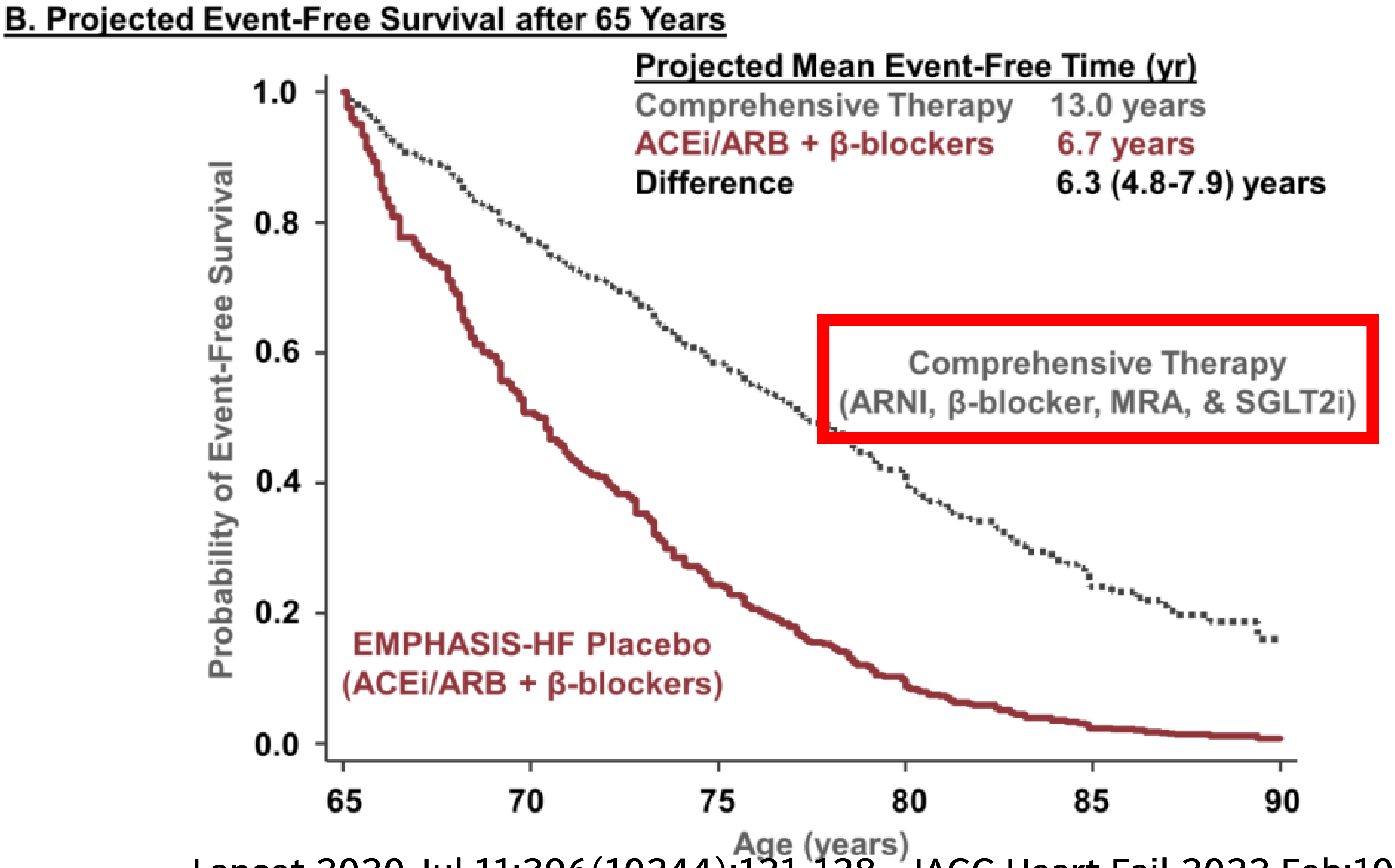

Lancet 2020 Jul 11;396(10244):121-128. JACC Heart Fail 2022 Feb;10(2):73-84.

Case 2 Q4

CENTRAL ILLUSTRATION: Relative Risk Reduction of Different Pharmacological Treatment Combinations for Heart Failure

A

Treatment	HR (95% CI) All-Cause Mortality
ARNI + BB + MRA + SGLT2	0.39 (0.31-0.49)
ARNI + BB + MRA + Vericiguat	0.41 (0.32-0.53)
ARNI + BB + MRA + Omecamtiv	0.44 (0.36-0.55)
ACEI + BB + Dig + H-ISDN	0.46 (0.35-0.61)
ACEI + BB + MRA + IVA	0.48 (0.39-0.58)
ACEI + BB + MRA + Vericiguat	0.49 (0.39-0.62)
ACEI + BB + MRA + Omecamtiv	0.52 (0.43-0.63)
ARNI + ARB + BB + Dig	0.65 (0.55-0.76)
ARNI + BB + MRA	0.44 (0.37-0.54)
ACEI + BB + MRA	0.52 (0.44-0.61)
ACEI + MRA + Dig	0.66 (0.56-0.78)
ACEI + BB + Dig	0.68 (0.59-0.78)
ARB + BB + Dig	0.73 (0.64-0.83)
ACEI + ARB + Dig	0.83 (0.72-0.96)
Dig + H-ISDN	0.67 (0.53-0.86)
ARNI + BB	0.58 (0.50-0.68)
ACEI + BB	0.69 (0.61-0.77)
ARB + BB	0.74 (0.66-0.82)
ACEI + Dig	0.87 (0.78-0.98)
ARB + Dig	0.94 (0.84-1.05)
BB	0.78 (0.72-0.84)
ACEI	0.89 (0.82-0.96)
ARB	0.95 (0.88-1.02)
Dig	0.99 (0.91-1.07)
PLBO	1.00

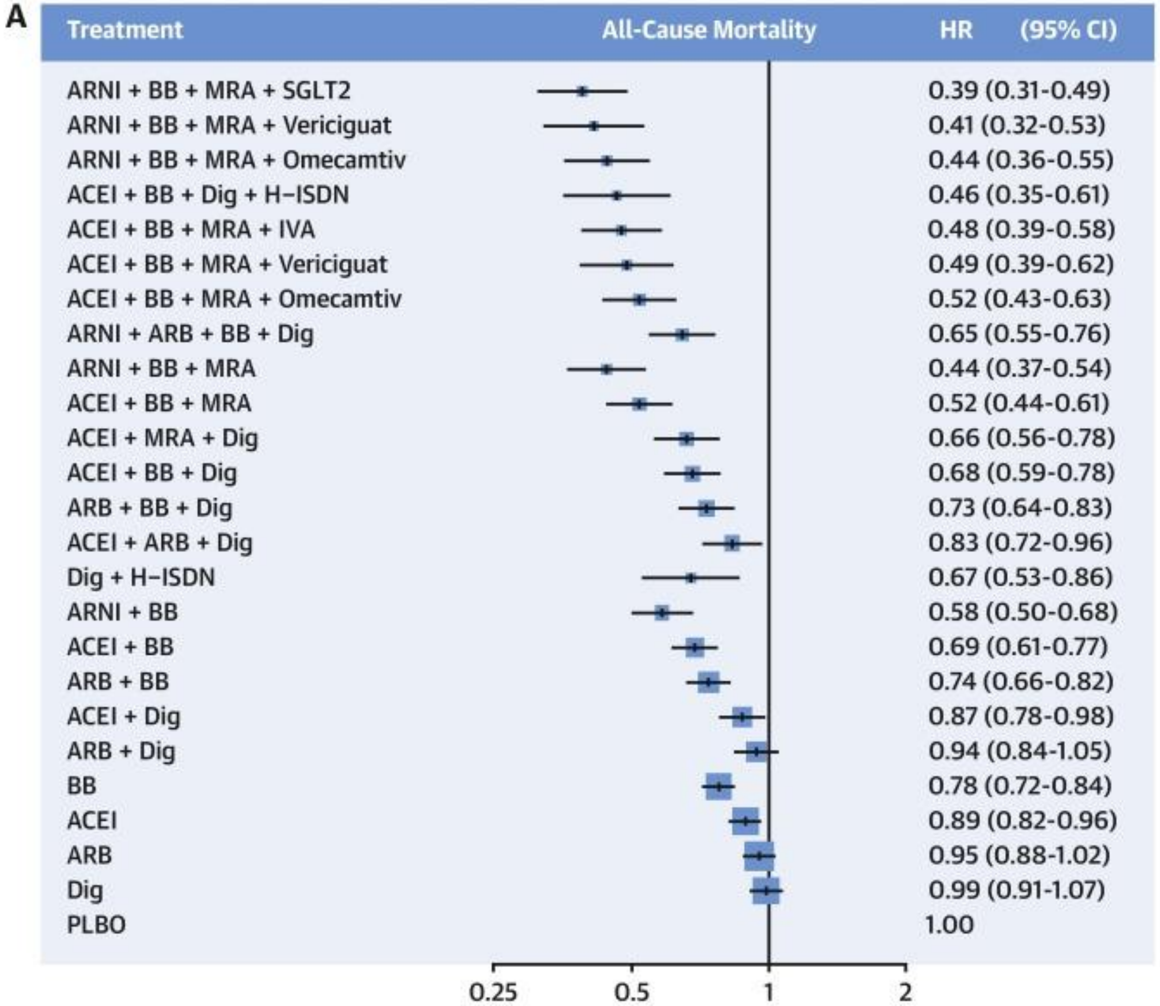

Tromp, J. et al. J Am Coll Cardiol HF. 2022;10(2):73-84.

CENTRAL ILLUSTRATION: Continued

B

Treatment	HR (95% CI) CV Mortality or HF Hospitalization
ARNI + BB + MRA + SGLT2	0.36 (0.29-0.46)
ARNI + BB + MRA + Vericiguat	0.43 (0.34-0.55)
ARNI + BB + MRA + Omecamtiv	0.44 (0.35-0.56)
ACEI + BB + MRA + IVA	0.49 (0.39-0.61)
ACEI + BB + MRA + Vericiguat	0.54 (0.43-0.67)
ACEI + ARB + BB + Dig	0.73 (0.62-0.85)
ARNI + BB + MRA	0.47 (0.38-0.58)
ACEI + BB + MRA	0.58 (0.47-0.71)
ARB + BB	0.65 (0.55-0.77)
ARNI + BB	0.68 (0.58-0.79)
ACEI + BB	0.84 (0.73-0.96)
ACEI + BB + Dig	0.84 (0.73-0.96)
ACEI + Dig	1.00
BB	0.75 (0.65-0.87)

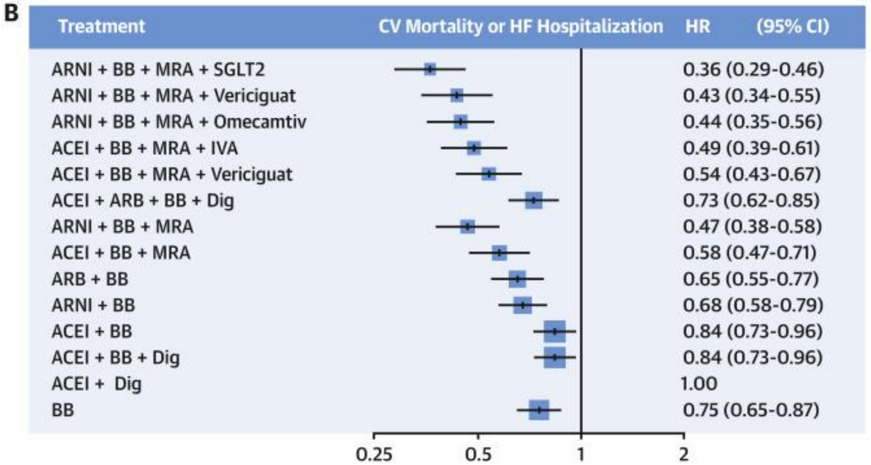

C

Treatment	HR (95% CI) CV Mortality
ARNI + BB + MRA + SGLT2	0.33 (0.26-0.43)
ARNI + BB + MRA + Vericiguat	0.35 (0.26-0.47)
ARNI + BB + MRA + Omecamtiv	0.36 (0.27-0.46)
ACEI + BB + MRA + IVA	0.43 (0.35-0.54)
ACEI + BB + MRA + Vericiguat	0.44 (0.33-0.57)
ACEI + BB + MRA + Omecamtiv	0.44 (0.35-0.56)
ACEI + BB + Dig + H-ISDN	0.57 (0.37-0.88)
ARNI + BB + MRA	0.38 (0.31-0.47)
ACEI + BB + MRA	0.47 (0.39-0.57)
ACEI + ARB + BB + Dig	0.57 (0.47-0.70)
ACEI + MRA + Dig	0.62 (0.52-0.74)
ACEI + BB + Dig	0.65 (0.56-0.76)
ACEI + BB	0.64 (0.56-0.73)
ARB + BB	0.68 (0.59-0.78)
ACEI + Dig	0.84 (0.75-0.96)
Dig + H-ISDN	0.88 (0.58-1.34)
ARB + Dig	0.89 (0.78-1.02)
BB	0.77 (0.70-0.85)
ACEI	0.83 (0.76-0.91)
ARB	0.88 (0.80-0.98)
PLBO	1.00
Dig	1.01 (0.93-1.10)

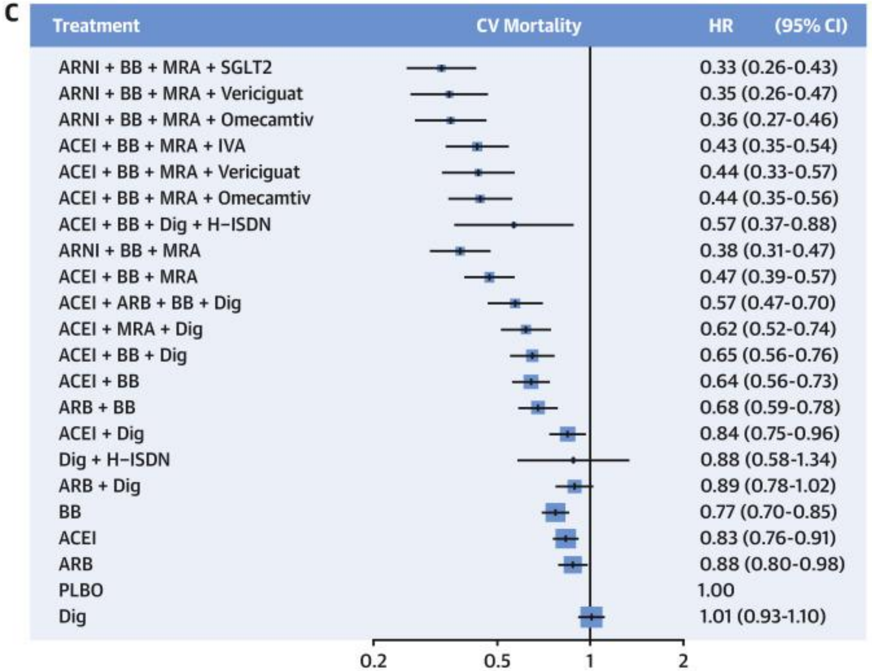

Tromp, J. et al. J Am Coll Cardiol HF. 2022;10(2):73-84.

JACC Heart Fail (IF: 12.04; Q1)2022 Feb;10(2):73-84.

Primary pharmacologic therapy for heart failure with reduced ejection fraction

- **Primary components of therapy** – In patients with HFrEF who have New York Heart Association (NYHA) class II to III symptoms (table 1), we suggest combination therapy with one agent from each of the following classes rather than other combinations (table 2) (**Grade 2C**) (see 'Primary components of therapy' above):
  - Angiotensin receptor blocker-neprilysin inhibitor (ARNI; ie, sacubitril-valsartan)
  - Beta blocker
  - Mineralocorticoid receptor antagonist (MRA)
  - Sodium-glucose co-transporter 2 (SGLT2) inhibitor (regardless of comorbid diabetes status

UpToDateでも同様の4剤を推奨する記載を認める

Case 2 Q4

感染のコントロールがついたため再度、心臓超音波検査を施行したところEF23%と低下を認めた。心不全の慢性期管理を開始する事とした。
この患者において、下記薬剤を組み合わせて内服した際に最も平均生存期間の延長に寄与するものはどれか？

a. ACEi/ARB + ARNI + SGLT2i + βblocker
b. ACEi/ARB +フロセミド + SGLT2i + βblocker
c. ARNI + SGLT2i + MRA + βblocker
d. フロセミド + SGLT2i + MRA + βblocker
e. ACEi/ARB + ARNI +フロセミド + SGLT2i

Q5 問題

Case 2 Q5 前半10 分

退院後、より良い心不全管理のために外来通院以外にも工夫することを検討している。次の選択肢の中で正しいものを全て答えなさい

a. 電話での健康教育･内服指導は、死亡率を改善させる
b. 電話での健康教育･内服指導は、再入院率を改善させる
c. 電話での健康教育･内服指導は、救急搬送される確率を下げる
d. テレモニタリング(患者の体重や血圧が医師に送信され、診療に利用すること)は、再入院率を改善させる
e. テレモニタリングは、全原因死亡率を低下させる

Case 2 Q5 後半5分

退院後、より良い心不全管理のために外来通院以外にも工夫することを検討している。次の選択肢の中で正しいものを全て答えなさい

a. 電話での健康教育・内服指導は、死亡率を改善させる
b. 電話での健康教育・内服指導は、再入院率を改善させる
c. 電話での健康教育・内服指導は、救急搬送される確率を下げる
d. テレモニタリング(患者の体重や血圧が医師に送信され、診療に利用すること)は、再入院率を改善させる
e. テレモニタリングは、全原因死亡率を低下させる

Q5 解説

Case 2 Q5

International Journal of
Environmental Research and Public Health

MDPI

Review

Effectiveness of Mobile Phone-Based Interventions for Improving Health Outcomes in Patients with Chronic Heart Failure: A Systematic Review and Meta-Analysis

Youn-Jung Son [1], Yaelim Lee [2] and Hyeon-Ju Lee [3,*]

1 Red Cross College of Nursing, Chung-Ang University, Seoul 06974, Korea; yjson@cau.ac.kr
2 College of Nursing, The Catholic University of Korea, Seoul 06591, Korea; ylcaregiver@gmail.com
3 Department of Nursing, Tongmyoung University, Busan 48520, Korea
* Correspondence: lhj209@tu.ac.kr; Tel.: +82-51-629-2687

Received: 13 January 2020; Accepted: 6 March 2020; Published: 7 March 2020

check for updates

Abstract: Mobile phone-based interventions are increasingly used to prevent adverse health outcomes in heart failure patients. However, the effects of mobile phone-based interventions on the health outcomes of heart failure patients remain unclear. Our review aims to synthesize the randomized controlled trials (RCT) of mobile phone-based interventions for heart failure patients and identify the intervention features that are most effective. Electronic searches of RCTs published from January 2000 to July 2019 were conducted. Primary outcomes included all-cause mortality, readmission, emergency department visits, length of hospital stays, and quality of life. Secondary outcomes were self-care behaviors, including medication adherence and other clinical outcomes. A total of eight studies with varying methodological quality met the inclusion criteria and were analyzed. Voice call intervention was more frequently used compared with telemonitoring and short message services. Our meta-analysis showed that voice call interventions had significant effects on the length of hospital stays. However, no significant effects on all-cause mortality, readmission, emergency department visits, or quality of life were found. Compared to other mobile phone-based interventions, voice calls were more effective in reducing the length of hospital stay. Future studies are needed to identify which features of mobile phone-based intervention most effectively improve health outcomes.

Keywords: heart failure; mobile phone; mortality; hospitalization; readmission; quality of life

1. All-cause mortality

Study	Country	Risk ratio	Lower limit	Upper limit	Z-value	P-value
Krum et al./ [30]	Australia	1.360	0.630	2.930	0.784	0.433
Chen et al.* [33]	China	0.958	0.578	1.587	-0.167	0.868
Chen et al.** [33]	China	0.801	0.471	1.362	-0.819	0.413
Total		0.954	0.685	1.327	-0.282	0.778

Risk Ratio and 95% CI

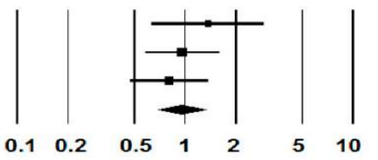

2. Readmission

Study	Country	SMD	Lower limit	Upper limit	Z-value	P-value
Seto et al. [29]	Canada	-0.474	-0.872	-0.077	-2.339	0.019
Hiter et al. [32]	Germany	-0.030	-0.176	0.117	-0.398	0.691
Total		-0.212	-0.641	0.217	-0.969	0.332

SMD and 95% CI

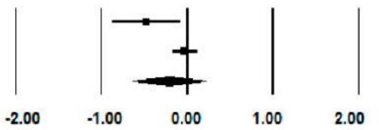

3. ED visits

Study	Country	SMD	Lower limit	Upper limit	Z-value	P-value
Reigel et al. [26]	USA	-0.078	-0.294	0.137	-0.712	0.477
Seto et al. [29]	Canada	-0.140	-0.575	0.294	-0.632	0.527
Total		-0.090	-0.283	0.103	-0.918	0.358

SMD and 95% CI

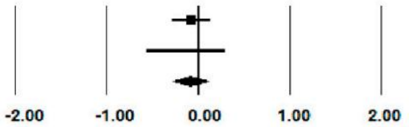

Int. J. Environ. Res. Public Health 2020, 17, 1749

Systems-based strategies to reduce hospitalizations in patients with heart failure

Telephone-based case management — Most disease management studies primarily involve telephone-based management, typically by a nurse; these studies have yielded mixed results. One meta-analysis found that of 47 RCTs of disease management, 40 involved some type of telephone follow-up. Among 26 low-quality RCTs involving community case management (largely but not entirely by telephone), the RR for mortality was 0.78 (95% CI 0.68-0.90) and for readmission the RR was 0.92 (95% CI 0.83-1.01). Meta-analyses of studies restricted to telephone support have largely found less benefit. For instance, one meta-analysis of nine RCTs of telephone support found no mortality benefit (RR 0.82, 95% CI 0.62-1.08) and no readmission benefit (RR 0.86, 95% CI 0.64-1.15). However, five of the six largest RCTs of disease management involved the telephone-based case management approach [49-53], some of which were successful:

- The largest study of chronic disease management reported to date evaluated eight Medicare Health Support pilot programs around the United States, which randomly assigned over 240,000 fee-for-service Medicare patients, most with HF, to usual care or participation in disease-management programs sponsored by Medicare [53]. These were commercial disease-management programs, not directly involved with the patient's clinical practice, and included nurse-led telephone-based chronic care services (symptom monitoring and management, medication counseling, health education, self-care coaching). Approximately 75 to 96 percent of patients in the intervention groups participated in the interventions. However, evaluators identified little improvement in self-care practices in the intervention group. After 18 months, evaluators found no significant difference between intervention and control groups in hospitalizations, readmissions, emergency department utilization, or mortality. Possible reasons for lack of success included the lack of integration of the disease-management program into the beneficiary's primary health care team, the infrequency of phone contact (on average 80 days between contact), contacts not coinciding with the time of disease flare or hospitalization, and the large number of comorbid illnesses in this patient population.

Case 2 Q5

Journal of Cardiac Failure Vol. 24 No. 1 2018

Clinical Investigation

Comparative Effectiveness of Telemonitoring Versus Usual Care for Heart Failure: A Systematic Review and Meta-analysis

JI EUN YUN, MPH, PhD,[1] JEONG-EUN PARK, MPH,[1] HYUN-YOUNG PARK, MD, PhD,[2] HAE-YOUNG LEE, MD, PhD,[3] AND DONG-AH PARK, MPH, PhD[1]

Seoul and Cheongju-si, Republic of Korea

ABSTRACT

Background: This study aimed to evaluate the effectiveness of telemonitoring (TM) in the management of patients with heart failure (HF).
Methods and Results: We searched Ovid-Medline, Ovid-Embase, and the Cochrane Library for randomized controlled trials published through May 2016. Outcomes of interest included clinical effectiveness (mortality, hospitalization, and emergency department visits) and patient-reported outcomes. TM was defined as the transmission of individual biologic data, such as weight, blood pressure, and heart rate. Thirty-seven randomized controlled trials (9582 patients) of TM met the inclusion criteria: 24 studies on all-cause mortality, 17 studies on all-cause hospitalization, 12 studies on HF-related hospitalization, and 5 studies on HF-related mortality. The risks of all-cause mortality (risk ratio [RR] 0.81, 95% confidence interval [CI] 0.70–0.94) and HF-related mortality (RR 0.68, 95% CI 0.50–0.91) were significantly lower in the TM group than in the usual care group. TM showed a significant benefit when ≥3 biologic data are transmitted or when transmission occurred daily. TM also reduced mortality risk in studies that monitored patients' symptoms, medication adherence, or prescription changes.
Conclusions: TM intervention reduces the mortality risk in patients with HF, and intensive monitoring with more frequent transmissions of patient data increases its effectiveness. (*J Cardiac Fail 2018;24:19–28*)
Key Words: Heart failure, telemonitoring, systematic review, meta-analysis.

Primary Outcome: All-Cause Mortality

Twenty-four studies reported all-cause mortality, and the pooled estimates showed a statistically significant reduction (19%) in all-cause mortality for TM, with low heterogeneity, compared with that of usual care (RR 0.81, 95% CI 0.70–0.94; $I^2 = 16\%$; Fig. 3). No significant difference between the

Table 3. Meta-analysis Results of All-Cause Hospitalization, HF Mortality, HF Hospitalization, and ED Visits

					Study Heterogeneity	
Outcome	No. of Studies	No. of Patients, TM/UC	RR*	95% CI	I^2 (%)	P Value
All-cause hospitalization						
Overall	17	3500/3482	0.94	0.85–1.03	67	<.0001
[illegible]						
Medium term	7	1958/1972	0.95	0.81–1.11	71	.002
Long term	8	1364/1332	0.91	0.77–1.06	74	.0004
HF mortality						
Overall	5	825/772	0.68	0.50–0.91	8	.36
Medium term	3	365/361	0.44	0.25–0.76	0	.86
Long term	2	460/411	0.80	0.58–1.10	0	.48
HF hospitalization						
Overall	12	2432/2388	0.86	0.74–1.00	36	.10
Short term	2	196/209	0.65	0.37–1.12	0	.32
Medium term	5	1190/1174	0.88	0.65–1.20	51	.09
Long term	5	1046/1005	0.85	0.70–1.02	19	.30
ED visit						
Overall	5	392/401	0.55	0.28–1.07	74	.004
Short term	1	101/116	0.96	0.43–2.12	NA	–
Medium term	2	227/221	0.35	0.03–4.70	85	.009
Long term	2	64/64	0.38	0.21–0.69	0	.79

Short term, follow-up <3 months; medium term, follow-up 3 to <12 months; long term, follow-up ≥12 months. CI, confidence interval; ED, emergency room; HF, heart failure; NA, not applicable; RR, risk ratio; TM, telemonitoring; UC, usual care.
*Mantel-Haenszel, random.

Int. J. Environ. Res. Public Health 2020, 17, 1749

Systems-based strategies to reduce hospitalizations in patients with heart failure

非侵襲的デバイスを使用した遠隔モニタリングは、全死亡率を低下させました（相対リスク0.81、95%信頼区間0

- A meta-analysis of 37 RCTs including 9582 patients reported on 24 studies with all-cause mortality and 17 studies with all-cause hospitalization. Telemonitoring with noninvasive devices reduced all-cause mortality (RR 0.81, 95% CI 0.70-0.94) but not all-cause hospitalizations (RR 0.94, 95% CI 0.85-1.03) [57].

UpToDateでも非侵襲的デバイスを使用した遠隔モニタリングは、
全死亡率を低下たが、全入院率には有意な影響を及ぼさない記載
